# Supplementary material for: Crude and adjusted comparisons of cesarean delivery rates using the Robson classification: A population-based cohort study in Canada and Sweden, 2004 to 2016
Source: PLoS Med. 2022 Aug 1;19(8):e1004077. doi: 10.1371/journal.pmed.1004077 (PMC9377587; doi:10.1371/journal.pmed.1004077)
Supplement: S1 Text — Protocol for “Epidemiologic evaluation of cesarean delivery trends in Canada and Sweden” study. (DOCX) [file pmed.1004077.s001.docx]

**Epidemiologic evaluation of cesarean delivery trends in Canada and Sweden**

**Background**

Over the past two decades, the rate of cesarean delivery (CD) in Canada has increased from 17.6% in 1995 to 27.9% in 2015.^1-3^ High rates of CD are potentially responsible for unnecessary utilization of health resources^4,5^ as well as maternal and neonatal harm.^6-11^ The reasons for the increase in CD rates are multifactorial and not well-understood. Changes in maternal characteristics and professional practice styles, increasing malpractice pressure, as well as economic, organizational, social and cultural factors have all been implicated in this trend.^12-15^ Recent data appears to suggest that CD rates below 20% are possible, safe, and compatible with optimal health outcomes for mothers and their newborns.^16,17^ For instance, Sweden has had a stable, relatively low CD rate for years (16.4% in 2003 and 17.4% in 2015),^18^ along with low rates of maternal and perinatal adverse outcomes.^19,20^ However, direct comparisons of CD rates between populations tends to be compromised by differences in obstetric profiles and risk factors for adverse perinatal outcomes.

In 2001, Robson proposed a classification system^21^ which provides a framework for evaluating geographic and temporal differences in rates of CD and permits the identification of subpopulations contributing to potential differences. This classification scheme stratifies deliveries into 10 mutually exclusive and all-inclusive categories based on obstetric characteristics such as parity, obstetric history (i.e., previous CD), type of labour onset and gestational age (Table 1). The groups created by such stratification are regarded as prognostically homogenous with regard to the risk of CD.^21^ Groups that make the greatest contribution to the overall rate of CD, and to population-level differences in CD rates, are therefore easy to identify. The Robson analysis thus offers maternity care providers and administrators information to guide prevention efforts directed at reducing CD rates.

Due to the simplicity and clarity of the Robson classification system, it is widely used in contemporary research on CD rates as well as in quality assurance and performance assessments.^22-33^ However, there are critical issues related to this classification system that have been neglected. Specifically, there are important extraneous determinants of spatial and temporal variations in CD rates that have not been integrated into the Robson analysis scheme.^34^ For example, maternal characteristics such as age, pre-pregnancy body mass index (BMI), and weight gain during pregnancy are three important determinants of CD rates that are not addressed by the Robson framework. Inferences about population-level differences in CD rates may be biased if they are based on patterns within the Robson’s groups but without appropriate consideration of these other extraneous factors that also influence CD rates.^34^ These issues has been addressed in the literature and hence represents a critical knowledge gap for women, clinicians, public health policy-makers, and related stakeholders.

**Table 1. Robson classification system^21^**

| 1 | Nulliparous women with a singleton fetus in vertex presentation at ≥37 wks gestation in spontaneous labour |
| --- | --- |
| 2 | Nulliparous women with a singleton fetus in vertex presentation at ≥37 wks gestation who either had labour induced or were delivered by cesarean section before labour |
| 3 | Multiparous women, without a previous uterine scar, with a singleton fetus in vertex presentation at ≥37 wks in spontaneous labour |
| 4 | Multiparous women, without a previous uterine scar, with a singleton fetus in vertex presentation at ≥37 wks gestation who either had labour induced or were delivered by cesarean section before labour |
| 5 | All multiparous women with at least one previous uterine scar and a singleton fetus in vertex presentation at ≥37 wks gestation |
| 6 | All nulliparous women with a singleton fetus in breech presentation |
| 7 | All multiparous women with a singleton fetus in breech presentation including, women with a previous uterine scar |
| 8 | All women with multifetal pregnancies, including women with a previous uterine scar |
| 9 | All women with a singleton fetus in a transverse or oblique presentation, including women with a previous uterine scar |
| 10 | All women with a singleton fetus in vertex presentation at ≤36 wks gestation, including women with a previous uterine scar |

**Objectives**

1. To identify subpopulations responsible for differences in CD rates between Canada and Sweden using the Robson classification *(Aim I)*;
2. To contrast the subpopulations identified in *Aim I* in Canada and Sweden using analyses that incorporate between-country differences in maternal age, pre-pregnancy BMI and weight gain in pregnancy *(Aim II)*;

**Hypotheses**

1. Differences in maternal characteristics in the Canadian and Swedish populations, such as age, pre-pregnancy BMI and weight gain in pregnancy, account for a significant proportion of the higher rates of CD observed in the Canadian population.

**Methods**

We propose to use a retrospective cohort study design including all live births and stillbirths ≥ 22 weeks gestational age between 2004 and 2016 in British Columbia (BC), Canada and Sweden. Data will be obtained from the population-based perinatal data registries in BC and Sweden. Fetuses and infants with congenital malformations will be excluded.

**Data for the Canadian cohort** of deliveries will be obtained from the British Columbia Perinatal Database Registry (BCPDR). The BCPDR contains information on approximately 99% of births in the province of British Columbia. Validation studies show that the BCPDR is an accurate and comprehensive source of perinatal information with accuracy rates of 97% over key data fields (such as CD) and data missing in less than 0.01% of records.^36-39^

**Data for the Swedish cohort** will be obtained from the national Swedish Medical Birth Register which provides information on pregnancy, delivery and the newborn and includes data on more than 98% of all births in Sweden since 1973.^40^ All residents of Sweden have a unique personal identification number, which allows for linkages across different data platforms. In order to account for all measurable determinants of CD and adverse outcomes, women within the Swedish Medical Birth Register will be linked to other nationwide registers, including the National Patient Register which provides information on inpatient and outpatient care, including diagnoses coded according to the International Classification of Diseases (1964 to 2016).^41^

*Aim I: Analysis of differences in CD rates in BC and Sweden using the Robson classification system*

The unit of analysis will be a delivery (i.e., a woman), the primary outcome of interest will be the CD rate and the analysis will compare Canada vs. Sweden. Data for all births in each country will be stratified into the 10 Robson categories. The overall CD rate, the relative size of each Robson group, and the relative contribution of each group to the overall CD rate will be compared between the Canadian and Swedish cohorts.

*Analysis plan for Aim I*: Comparisons of CD rates between populations overall and by Robson group will be carried out by using the Chi-square test for differences in proportions. Statistical significance of differences will be assessed based on two-sided p-values and a p-value <0.05 will be considered statistically significant. The results of this stratified analysis will be used to make inferences about the subpopulations contributing to differences in CD rates in BC and Sweden and provide insight about where prevention efforts could be targeted.

*Aim II: Evaluating the ability of the Robson classification system to identify factors responsible for increases in the CD rate in Canada and Sweden*

The unit of analysis will be a delivery (i.e., a woman) and the primary outcome of interest will be CD. Maternal, fetal and obstetric practice factors of interest that are not included in the Robson classification scheme (Table 2) will be examined as potential determinants of CD within Robson strata.

*Analysis plan for Aim II:* Univariate summary statistics will be calculated on all determinants of interest. Continuous variables will be summarized using means, standard deviations and percentiles and compared using the Students t-test or non-parametric tests. Categorical variables will be summarized using percentages and contrasted using the Chi-square or Fisher’s exact tests. To understand the contribution of maternal characteristics and obstetric practice factors to increases in CD rates, we will carry out individual-level regression analyses using logistic regression. Differences in CD rates between BC and Sweden will be examined after adjusting for maternal, fetal, and obstetric factors (Table 2) and quantified using odds ratios and 95% confidence intervals. Sequential models will be used to identify the effect of each factor and each group of factors on the relationship between calendar year and CD. This analysis will be carried out on all women and also within each Robson group. Log-binomial regression analysis will be used to obtain unadjusted and adjusted rate ratios for potential determinants of CD. Crude and adjusted effects will be contrasted to determine whether controlling for changes in maternal, fetal, and obstetric factors will attenuate the differences in CD rates. Confounding variables, mediators and effects modifiers will be distinguished in order to avoid bias. A two-sided p-value of <0.05 will be considered statistically significant. The results of this regression analysis will be used to make inferences about factors contributing to observed differences in CD rates between populations and within populations over time. The inferences made in Aim I will be contrasted with the inferences made in Aim II to determine whether the Robson classification scheme supports appropriate inferences or whether it needs to be supplemented with regression analysis that controls for all extraneous determinants of CD.

**Table 2. Determinants of cesarean delivery**

| **In Robson** | **Not in Robson** | |
| --- | --- | --- |
| **Maternal characteristics** | |  |
| Parity (nulliparous vs parous) | Age | Assisted reproductive technology |
| Previous CD | Body mass index | Preeclampsia/eclampsia |
|  | Parity (in parous women, 1, 2, 3-4, ≥5) | Pre-pregnancy Diabetes |
|  | Maternal weight gain | Chronic hypertension |
|  | Smoking status | Substance use |
| **Obstetric factors** | |  |
| Induction | Epidural anesthesia | Post-term delivery |
| Labour/no labour |  |  |
| **Fetal/infant characteristics** | |  |
| Singleton/Multiple (twin or higher order) | Position of the fetal head at delivery (for cephalic-presenting fetuses, OP vs OA) | Birth weight |
| Gestational age (term/preterm) | Congenital anomaly | Head circumference |
| Presentation |  |  |

**Study power:** We expect to have approximately 560,000 births in BC^42^ and 1,340,000 births in Sweden between 2004 and 2016.^18^ This study size will result in greater than 99% power to detect as significant a 5% or greater difference in CD rates while controlling for maternal, fetal, and obstetric practice factors.

**References**

1. Health Canada. Canadian perinatal health report, 2003 Edition. Ottawa, ON, 2003.
2. Public Health Agency of Canada. Canadian perinatal health report, 2008 Edition. Ottawa, ON, 2008.
3. Canadian Institute for Health Information (CIHI). Quick Stats: Childbirth indicators by place of residence. Report ID: CB1. Ottawa, ON, 2017. Accessed September 15, 2017 from https://apps.cihi.ca/mstrapp/asp/Main.aspx?Server=apmstrextprd_i&project= Quick%20Stats&uid=pce_pub_en&pwd=&evt=2048001&visualizationMode=0&documentID=029DB170438205AEBCC75B8673CCE822.
4. Gibbons L , Belizán JM, Lauer JA, Betrán AP, Merialdi M, Althabe F. The global numbers and costs of additionally needed and unnecessary caesarean sections performed per year: overuse as a barrier to universal coverage. World Health Report Background Paper: 2010. Accessed September 15, 2017 from http://www.who.int/ healthsystems/topics/ financing/healthreport/30C-sectioncosts.pdf.
5. CIHI. Giving birth in Canada: The costs. Ottawa, ON, 2006. Accessed September 15, 2017 from https://secure.cihi.ca/free_products/Costs_Report_06_Eng.pdf.
6. Gregory KD, Jackson S, Korst L, Fridman M. Cesarean versus vaginal delivery: whose risks? Whose benefits? Am J Perinatol. 2012;29(1):7–18. pmid:21833896
7. Huang X, Lei J, Tan H, Walker M, Zhou J, Wen SW. Cesarean delivery for first pregnancy and neonatal morbidity and mortality in second pregnancy. Eur J Obstet Gynecol Reprod Biol, 2011;158(2):204–8.
8. Timor-Tritsch IE, Monteagudo A. Unforeseen consequences of the increasing rate of cesarean deliveries: early placenta accreta and cesarean scar pregnancy: A review. Am J Obstet Gynecol, 2012;207(1):14–29.
9. Marshall NE, Fu R, Guise JM. Impact of multiple cesarean deliveries on maternal morbidity: a systematic review. Am J Obstet Gynecol, 2011;205(3):262 e1-8.
10. Lumbiganon P, Laopaiboon M, Gulmezoglu AM, Souza JP, Taneepanichskul S, Ruyan P, et al. Method of delivery and pregnancy outcomes in Asia: the WHO global survey on maternal and perinatal health 2007–08. Lancet, 2010;375(9713):490–9.
11. Souza JP, Gulmezoglu A, Lumbiganon P, Laopaiboon M, Carroli G, Fawole B, et al. Caesarean section without medical indications is associated with an increased risk of adverse short-term maternal outcomes: the 2004–2008 WHO Global Survey on Maternal and Perinatal Health. BMC medicine, 2010;8:71.
12. Lin HC, Xirasagar S. Institutional factors in cesarean delivery rates: policy and research implications. Obstet Gynecol, 2004;103(1):128–36.
13. Linton A, Peterson MR, Williams TV. Effects of maternal characteristics on cesarean delivery rates among U.S. Department of Defense healthcare beneficiaries, 1996–2002. Birth, 2004;31(1):3–11.
14. Zwecker P, Azoulay L, Abenhaim HA. Effect of fear of litigation on obstetric care: a nationwide analysis on obstetric practice. Am J Perinatol, 2011;28(4):277–84.
15. Mi J, Liu F. Rate of caesarean section is alarming in China. Lancet, 2014;383(9927): 1463–4.
16. Ye J, Betran AP, Guerrero Vela M, Souza JP, Zhang J. Searching for the optimal rate of medically necessary cesarean delivery. Birth, 2014;41:237-44.
17. Molina G, Weiser TG, Lipsitz SR, et al. Relationship between cesarean delivery rate and maternal and neonatal mortality. JAMA, 2015;314:2263-70.
18. The National Board of Health and Welfare, Sweden. Statistics on Pregnancies, Deliveries and Newborn Infants 2015. Article #: 2017-3-4. Stockholm, Sweden, 2017. Accessed on September 16, 2017 from http://www.socialstyrelsen.se/SiteCollection Documents/2017-3-3-tabeller.xls.
19. Zeitlin J, Mohangoo AD, Delnord M, Cuttini M and the EURO-PERISTAT Scientific Committee.The second European Perinatal Health Report: documenting changes over 6 years in the health of mothers and babies in Europe. J Epidemiol Community Health, 2013;67(12):983-5.
20. Zeitlin J, Mortensen L, Cuttini M and the EURO-PERISTAT Scientific Committee. Declines in stillbirth and neonatal mortality rates in Europe between 2004 and 2010: results from the Euro-Peristat project J Epidemiol Community Health 2016;70:609-615.
21. Robson MS. Can we reduce the caesarean section rate? Best Pract Res Clin Obstet Gynaecol, 2001;15:179-94.
22. Kelly S, Sprague A, Fell DB, Murphy P, Aelicks N, Guo Y, et al. Examining caesarean section rates in Canada using the Robson classification system. J Obstet Gynaecol Can, 2013;35:206-14.
23. Abdel-Aleem H, Darwish A, Abdelaleem AA, Mansur M. Usefulness of the WHO C-Model to optimize the cesarean delivery rate in a tertiary hospital setting. Int J Gynaecol Obstet, 2017;137(1):40-44.
24. Guida JP, Pacagnella RC, Costa ML, Ferreira EC, Cecatti JG. Evaluating vaginal-delivery rates after previous cesarean delivery using the Robson 10-group classification system at a tertiary center in Brazil. Int J Gynaecol Obstet, 2017;136(3):354-355.
25. Farine D, Shepherd D. Classification des césariennes au Canada : Les critères modifiés de Robson. J Obstet Gynaecol Can, 2016 ;38(12S):S153-S157.
26. Gerli S, Favilli A, Franchini D, De Giorgi M, Casucci P, Parazzini F. Is the Robson's classification system burdened by obstetric pathologies, maternal characteristics and assistential levels in comparing hospitals cesarean rates? A regional analysis of class 1 and 3. J Matern Fetal Neonatal Med, 2017;26:1-8.
27. Nakamura-Pereira M, do Carmo Leal M, Esteves-Pereira AP, Domingues RM, Torres JA, Dias MA, Moreira ME. Use of Robson classification to assess cesarean section rate in Brazil: the role of source of payment for childbirth. Reprod Health, 2016;13(Suppl 3):128.
28. Atnurkar KB, Mahale AR. Audit of Caesarean Section Births in Small Private Maternity Homes: Analysis of 15-Year Data Applying the Modified Robson Criteria, Canada. J Obstet Gynaecol India, 2016;66(Suppl 1):289-94.
29. Yadav RG, Maitra N. Examining Cesarean Delivery Rates Using the Robson's Ten-group Classification. J Obstet Gynaecol India, 2016;66(Suppl 1):1-6.
30. Ono T, Matsuda Y, Sasaki K, Satoh S, Tsuji S, Kimura F, Murakami T. Comparative analysis of cesarean section rates using Robson Ten-Group Classification System and Lorenz curve in the main institutions in Japan, J Obstet Gynaecol Res, 2016;42(10):1279-1285.
31. Fatusic J, Hudic I, Fatusic Z, Zildzic-Moralic A, Zivkovic M. Cesarean Section Rate Analysis in University Hospital Tuzla - According to Robson's Classification. Med Arch, 2016;70(3):213-6.
32. Tan JK, Tan EL, Kanagalingam D, Yu SL, Tan LK. Multiple pregnancy is the leading contributor to cesarean sections in in vitro fertilization pregnancies: An analysis using the Robson 10-group classification system. J Obstet Gynaecol Res, 2016;42(9):1141-5.
33. Jayot A, Nizard J. Evolution of cesarean categories in a modified Robson classification in a single center from 2002 to 2012 due to high rate of maternal pathology. J Obstet Gynaecol Res, 2016;42(6):648-54.
34. Joseph KS, Young DC, Dodds L, O'Connell CM, Allen VM, Chandra S, et al. Changes in maternal characteristics and obstetric practice and recent increases in primary cesarean delivery. Obstet Gynecol, 2003;102:791-800.
35. Dunn S, Bottomley J, Ali A, Walker M. 2008 Niday Perinatal Database quality audit: report of a quality assurance project. Chronic Dis Inj Can, 2011;32:32-42.
36. British Columbia Reproductive Care Program. British Columbia Perinatal Data Registry overview. Vancouver, BC: 2003.
37. Frosst G, Hutcheon J, Joseph KS, Kinniburgh B, Johnson C, Lee L. Validating the British Columbia Perinatal Data Registry: a chart re-abstraction study. BMC Pregnancy Childbirth, 2015;15:123.
38. Frosst G. Validity of pre-pregnancy body mass index (BMI) information derived from a population-based perinatal database. Healthy Mothers and Healthy Babies: New Research and Best Practice Conference 2014. Vancouver, British Columbia.
39. Joseph KS, Fahey J. Validation of perinatal data in the Discharge Abstract Database of the Canadian Institute for Health Information. Chronic Dis Can, 2009;29:96–100.
40. The National Board of Health and Welfare, Sweden. The Swedish Medical Birth Register. Accessed September 15, 2017 from http://www.socialstyrelsen.se/register/ halsodata register/medicinskafodelseregistret/inenglish.
41. The Swedish Centre for Epidemiology. The Swedish Medical Birth Register: A summary of content and quality. 2003. Accessed on September 15, 2017 from http://www.social styrelsen.se/Lists/Artikelkatalog/ Attachments/10655/2003-112-3_20031123.pdf.
42. Statistics Canada. Births, estimates, by province and territory. Accessed September 19, 2017 from http://www.statcan.gc.ca/tables-tableaux/sum-som/l01/cst01/demo04a-eng.htm.
43. Muraca GM, Sabr Y, Brant R, Cundiff GW, Joseph KS. Temporal and regional variations in operative vaginal delivery in Canada by pelvic station, 2004–2012. J Obstet Gynaecol Can, 2016;38:627–35.
44. Muraca GM, Sabr Y, Lisonkova S, Skoll A, Brant R, Cundiff GW, Joseph KS. Perinatal and maternal morbidity and mortality after attempted operative vaginal delivery at midpelvic station. CMAJ, 2017;189(22):E764-72.
45. Muraca GM, Skoll A, Lisonkova S, Sabr Y, Brant R, Cundiff GW, Joseph KS. BJOG. 2017; doi: 10.1111/1471-0528.14820.
46. Villar J, Valladares E, Wojdyla D, Zavaleta N, Carroli G, Velazco A, et al. Caesarean delivery rates and pregnancy outcomes: the 2005 WHO global survey on maternal and perinatal health in Latin America. Lancet, 2006;367:1819-29.
47. Althabe F, Sosa C, Belizán JM, Gibbons L, Jacquerioz F, Bergel E. Cesarean section rates and maternal and neonatal mortality in low, medium, and high-income countries: an ecological study. Birth, 2006;33:270-7.
48. Betrán AP, Merialdi M, Lauer JA, Bing-Shun W, Thomas J, Van Look P, et al. Rates of caesarean section: analysis of global, regional and national estimates. Paediatr Perinat Epidemiol, 2007;21:98-113.
49. Betrán AP, Ye J, Moller AB, Zhang J, Gülmezoglu AM, et al. The increasing trend in caesarean section rates: global, regional and national estimates: 1990-2014. PLOS ONE, 2016;11(2):e0148343.
