# Supplementary material for: Crude and adjusted comparisons of cesarean delivery rates using the Robson classification: A population-based cohort study in Canada and Sweden, 2004 to 2016
Source: PLoS Med. 2022 Aug 1;19(8):e1004077. doi: 10.1371/journal.pmed.1004077 (PMC9377587; doi:10.1371/journal.pmed.1004077)
Supplement: S1 Table — Steps defined by the World Health Organization to assess quality of data, type of population, and cesarean delivery rates using the Robson classification. (DOCX) [file pmed.1004077.s003.docx]

S1 Table. Assessment of quality of data, type of population, and cesarean delivery (CD) rates [1]

| **Step** | **Interpretation by Robson [1]** | **Swedish population** | **British Columbian population** | **WHO MCS reference population [2,3]** | **Interpretation** |
| --- | --- | --- | --- | --- | --- |
| Data quality | | | | | |
| Look at the size of Group 9 (singletons in transverse/ oblique lie) | It should be less than 1%. | 0.1% | 0.3% | 0.4% | As expected. |
| Look at the CD rate of Group 9 | It should be 100%. | 99.3 | 94.7 | 88.6% | Minor misclassification. |
| Type of population | | | | | |
| Look at the size of Groups 1 + 2 (nulliparous women ≥37 weeks gestation singleton cephalic) | This usually represents 35-42% of the obstetric population. | 39.2% | 39.2% | 38.1% | As expected. |
| Look at the size of Groups 3 + 4 (multiparous women ≥37 weeks gestation singleton cephalic, without previous CD) | This usually represents about 30% of women. | 43.0% | 34.3% | 46.5% | As expected. Higher in Sweden due to a high proportion of multiparas without a previous CD. |
| Look at the size of Group 5 (multiparous women ≥37 weeks gestation singleton cephalic with previous CD) | It is related to the overall CD rate. The size of Group 5 is usually about half of the total CD rate. In settings with low overall CD rates, it is usually under 10%. | 8.6% | 12.8% | 7.2% | As expected. Size of Group 5 in British Columbia similar to those reported in national US and Canadian analyses [4,5]. |
| Look at the size of Groups 6 + 7 (breeches in nulliparous & multiparous women) | It should be 3-4%. | 3.2% | 3.9% | 2.7% | As expected. |
| Look at the size of Groups 8 (multiple gestations) | It should be 1.5 -2%. | 1.4% | 1.6% | 0.9% | As expected. |
| Look at the size of Group 10 (preterm cephalic singletons) | It should be less than 5% in most normal risk settings. | 4.2% | 6.8% | 4.2% | Higher rates in British Columbia suggest higher rates of provider-initiated pre-labour CD. Similar to Group size reported in US and Canadian national analyses [4,5]. |
| Look at the ratio of the size of Group 1 vs Group 2 (nullipara term cephalic singletons spontaneous labour/nullipara term cephalic singletons induced or pre-labour CD) | It is usually 2:1 or higher | 3.8:1 | 2.2:1 | 3.3:1 | As expected. |
| Look at the ratio of the size of Group 3 vs Group 4 (multipara without previous CD, term cephalic singletons spontaneous labour/multipara without previous CD, term cephalic singletons induced or pre-labour CD) | It is always higher than the ratio of Group 1/Group 2 in the same population, i.e., larger than 2:1. | 5.1:1 | 3.7:1 | 6.3:1 | As expected. This is very reliable finding in confirming data quality and culture of the organization. [1] |
| Look at the ratio of the size of Group 6 vs Group 7 (nullipara breech /multipara breech) | It is usually a 2:1 because breeches are more frequent in nulliparous women than in multiparous women. | 1.7:1.0 | 1.4:1.0 | 0.8:1 | As expected. |
| Assessing CD rates | | | | | |
| Look at the CD rate for Group 1 | Rates under 10% are achievable | 8.1% | 20.4% | 9.8% | High rates of CD in British Columbia are consistent with previous reports using different data sources [5]. Decomposing this high rate is one of the foci of the present analysis. |
| Look at the CD rate for Group 2 | Approximately 20-35% | 37.3% | 45.9% | 39.9% | Since Group 2b is relatively small, the high rates of CD in Group 2 in both Sweden and British Columbia are a result of the high CD rate in Group 2a. This may indicate poor success rates for induction or poor choice of women to induce and consequently a high rate of CD in Group 2a. High rates of CD in British Columbia are consistent with previous reports using different data sources [5]. |
| Look at the CD rate for Group 3 | Normally, no higher than 3.0%. | 1.6% | 2.6% | 3.0% | As expected. |
| Look at the CD rate for Group 4 | It rarely should be higher than 15% | 21.5% | 13.1% | 23.7% | High in Sweden since the size of Group 4b (pre-labour CD) is large. May indicate a data issue whereby women with a previous CD are included (when they should be in Group 5). May also be due to high rates of CD on maternal request due to previously traumatic or prolonged labour. |
| Look at the CD rate for Group 5 | Rates of 50-60% are considered appropriate provided you have good maternal and perinatal outcome. | 51.6% | 81.3% | 74.4% | As expected. Higher rates of CD in British Columbia consistent with rates in North America where a policy of scheduling pre-labour CD for women with a previous scar without attempting a trial of labour is common [4,5]. |
| Look at the CD rate for Group 8 | It is usually around 60%. | 54.7% | 69.6% | 57.7% | As expected. Higher rates in British Columbia expected due to higher number of women with a previous CD. Similar to national estimates in the US and Canada [4,5]. |
| Look at the CD rate for Group 10 | In most populations it is usually around 30% | 29.4% | 30.8% | 25.1% | As expected. |
| Look at the relative contribution of Groups 1, 2 and 5 to the overall CD rate | These three groups combined normally contribute to 2/3 (66%) of all CD performed in most hospitals. | 57.8% | 69.0% | 63.7% | As expected. Higher relative contribution in British Columbia due to higher rates of CD in both Groups 1 and 2. These higher rates in previous years are responsible for the larger size of Group 5 in British Columbia. This larger size plus the and higher rates of CD in British Columbia in Group 5 drive the higher contribution of these three groups compared with Sweden (and the MCS population). |
| Look at the relative contribution of Group 5 to the overall CD rate |  | 25.7% | 33.4% | 28.9% | Appropriate. Higher in British Columbia since CD rates in Groups 1 and 2 have been high in previous years. |

WHO MCS, World Health Organization Multi-Country Survey; CD, cesarean delivery.

MCS reference population was the population of the MCS with relatively low CD rates and, at the same time, with good outcomes of labour and childbirth.

References

1. World Health Organization. Robson Classification: Implementation Manual. Licence: CCBY-NC-SA3.0IGO. Geneva: World Health Organization; 2017.
2. Souza JP, Betrán AP, Dumont A, de Muncio B, Gibbs Pickens C, Deneux-Tharaux C, et al. A global reference for caesarean section rates (C-Model): a multi-country cross-sectional study. BJOG. 2016;123:427-36.
3. Souza JP, Gülmezoglu AM, Vogel J, Carroli G, Lumbiganon P, Qureshi Z, et al. Moving beyond essential interventions for reduction of maternal mortality (the WHO Multi-country Survey on Maternal and Newborn Health): a cross-sectional study. Lancet. 2013;18:1747-55.
4. Hehir MP, Ananth CV, Siddiq Z, Flood K, Friedman AM, D’Alton ME. Cesarean delivery in the United States 2005 through 2014: a population-based analysis using the Robson 10-Group Classification System. Am J Obstet Gynecol. 2018;219:105.e1–11.
5. Gu J, Karmakar-Hore S, Hogan ME, Azzam HM, Barrett JF, Brown A, et al. Examining cesarean section rates in Canada using the modified Robson classification. J Obstet Gynaecol Can. 2020;42:757-65.
