# Supplementary material for: Crude and adjusted comparisons of cesarean delivery rates using the Robson classification: A population-based cohort study in Canada and Sweden, 2004 to 2016
Source: PLoS Med. 2022 Aug 1;19(8):e1004077. doi: 10.1371/journal.pmed.1004077 (PMC9377587; doi:10.1371/journal.pmed.1004077)
Supplement: S2 Table — To quantify comparisons of cesarean delivery rates in British Columbia vs. Sweden between Robson groups, we calculated odds ratios and 95% confidence intervals for Robson group-specific CD rates since the odds ratio (unlike the rate ratio) is not susceptible to artefactual constraints when the baseline CD rate is high. (DOCX) [file pmed.1004077.s004.docx]

| Robson Group |  | Crude cesarean delivery rate (%) | |  | Cesarean delivery  British Columbia vs Sweden | | | | |
| --- | --- | --- | --- | --- | --- | --- | --- | --- | --- |
|  |  | Sweden | British Columbia |  | OR (95% CI) | P-value* |  | AOR^†^ (95% CI) | P-value* |
| 1 |  | 8.1 | 20.4 |  | 2.91 (2.87-2.96) | <0.001 |  | 3.09 (3.04-3.16) | <0.001 |
| 2 |  | 37.3 | 45.9 |  | 1.43 (1.40-1.46) | <0.001 |  | 1.22 (1.19-1.24) | <0.001 |
| 3 |  | 1.6 | 2.6 |  | 1.61 (1.55-1.67) | <0.001 |  | 1.70 (1.63-1.78) | <0.001 |
| 4 |  | 21.5 | 13.1 |  | 0.55 (0.53-0.57) | <0.001 |  | 0.40 (0.39-0.42) | <0.001 |
| 5 |  | 51.6 | 81.3 |  | 4.09 (4.00-4.18) | <0.001 |  | 3.42 (3.34-3.50) | <0.001 |
| 6 |  | 93.8 | 95.5 |  | 1.40 (1.27-1.54) | <0.001 |  | 1.36 (1.22-1.51) | <0.001 |
| 7 |  | 88.5 | 90.2 |  | 1.19 (1.10-1.30) | <0.001 |  | 1.16 (1.06-1.28) | 0.001 |
| 8 |  | 54.7 | 69.6 |  | 1.89 (1.80-2.00) | <0.001 |  | 1.66 (1.56-1.75) | <0.001 |
| 9 |  | 99.3 | 94.7 |  | 0.12 (0.07-0.22) | <0.001 |  | 0.12 (0.06-0.21) | <0.001 |
| 10 |  | 29.4 | 30.8 |  | 1.07 (1.04-1.10) | <0.001 |  | 1.19 (1.15-1.23) | <0.001 |

S2 Table. Crude and adjusted rates and odds ratios for cesarean delivery in British Columbia vs Sweden by Robson group, 2004-2016

OR, odds ratio; CI, confidence interval; AOR, adjusted odds ratio.

*P-values represent significance of Wald chi-square test; the a priori level of statistical significance was set at a 2-sided p value<0.05.

†Adjusted models included maternal age, parity, early-pregnancy body mass index, smoking during pregnancy, chronic hypertension, pre-existing diabetes, in-vitro fertilization, preeclampsia/eclampsia, post-term delivery, position of the fetal head at delivery, infant birth weight, infant head circumference, congenital anomaly.

^a^Adjusted model also included epidural anaesthesia.

^b^Adjusted model excluded parity due to group restriction to nulliparas.

^c^Adjusted model excluded position of the fetal head at delivery due to group restriction to non-cephalic fetal presentation or multiple gestation.

^d^Adjusted model excluded post-term delivery due to group restriction to preterm deliveries.
