# Supplementary material for: Crude and adjusted comparisons of cesarean delivery rates using the Robson classification: A population-based cohort study in Canada and Sweden, 2004 to 2016
Source: PLoS Med. 2022 Aug 1;19(8):e1004077. doi: 10.1371/journal.pmed.1004077 (PMC9377587; doi:10.1371/journal.pmed.1004077)
Supplement: S3 Table — Robson group-specific cesarean delivery rates by year, percent change in cesarean delivery rates, and p-value for linear trend over the study period in Sweden. (DOCX) [file pmed.1004077.s005.docx]

S3 Table. Cesarean delivery rate by year of delivery and Robson Group, Sweden, 2004-2016

| Robson Group | 2004 | 2005 | 2006 | 2007 | 2008 | 2009 | 2010 | 2011 | 2012 | 2013 | 2014 | 2015 | 2016 | % change* | P-value† |
| --- | --- | --- | --- | --- | --- | --- | --- | --- | --- | --- | --- | --- | --- | --- | --- |
| 1 | 8.1 | 8.1 | 8.5 | 8.4 | 8.3 | 8.5 | 8.1 | 7.8 | 8.1 | 8.1 | 7.9 | 7.6 | 7.7 | -4.42 | <0.001 |
| 2a | 28.6 | 29.5 | 30.2 | 28.9 | 29.2 | 28.3 | 27.6 | 26.5 | 26.2 | 27.3 | 25.6 | 24.5 | 24.3 | -15.04 | <0.001 |
| 2b | 100.0 | 100.0 | 100.0 | 100.0 | 100.0 | 100.0 | 100.0 | 100.0 | 100.0 | 100.0 | 100.0 | 100.0 | 100.0 | 0.00 | NA^‡^ |
| 3 | 1.6 | 1.7 | 1.8 | 1.8 | 1.6 | 1.7 | 1.6 | 1.6 | 1.6 | 1.5 | 1.5 | 1.4 | 1.5 | -2.35 | <0.001 |
| 4a | 5.7 | 6.6 | 6.8 | 6.4 | 5.9 | 5.7 | 5.2 | 5.4 | 5.2 | 5.0 | 4.9 | 4.8 | 4.5 | -21.04 | <0.001 |
| 4b | 100.0 | 100.0 | 100.0 | 100.0 | 100.0 | 100.0 | 100.0 | 100.0 | 100.0 | 100.0 | 100.0 | 100.0 | 100.0 | 0.00 | NA^‡^ |
| 5 | 49.4 | 50.7 | 51.3 | 51.3 | 50.0 | 51.5 | 50.8 | 51.8 | 50.8 | 52.1 | 53.6 | 53.2 | 52.8 | 6.86 | <0.001 |
| 6 | 94.1 | 94.9 | 94.8 | 94.7 | 93.2 | 93.5 | 93.4 | 93.1 | 93.5 | 93.8 | 93.7 | 93.5 | 93.6 | -0.59 | 0.02 |
| 7 | 89.6 | 90.4 | 90.4 | 90.4 | 87.4 | 89.5 | 87.5 | 87.8 | 88.0 | 87.7 | 88.3 | 85.6 | 87.8 | -1.91 | <0.001 |
| 8 | 55.8 | 56.2 | 53.9 | 52.7 | 52.7 | 54.3 | 54.7 | 55.1 | 55.6 | 53.7 | 57.4 | 54.9 | 54.2 | -2.83 | 0.6 |
| 9 | 98.0 | 98.6 | 100.0 | 100.0 | 99.2 | 99.3 | 98.1 | 100.0 | 100.0 | 99.3 | 98.7 | 100.0 | 100.0 | 2.08 | 0.1 |
| 10 | 29.1 | 28.9 | 30.6 | 30.0 | 28.3 | 27.2 | 28.3 | 30.0 | 29.4 | 29.1 | 30.4 | 29.9 | 30.5 | 4.80 | 0.1 |
| Unknown | 51.5 | 53.4 | 51.3 | 39.2 | 53.9 | 50.6 | 34.9 | 51.8 | 55.1 | 63.5 | 71.1 | 48.7 | 52.5 | 2.08 | 0.01 |
| All groups | 16.8 | 17.2 | 17.8 | 17.6 | 17.2 | 17.5 | 17.0 | 17.0 | 17.1 | 17.3 | 17.7 | 17.4 | 17.6 | 4.75 | 0.1 |

*Percent change in cesarean delivery rate in 2016 vs 2004.

†P-value of Cochran-Armitage test for linear trend in cesarean delivery rate by year of delivery.

^‡^Groups 2b and 4b are restricted to women with a cesarean delivery.
