# Supplementary material for: Crude and adjusted comparisons of cesarean delivery rates using the Robson classification: A population-based cohort study in Canada and Sweden, 2004 to 2016
Source: PLoS Med. 2022 Aug 1;19(8):e1004077. doi: 10.1371/journal.pmed.1004077 (PMC9377587; doi:10.1371/journal.pmed.1004077)
Supplement: S4 Table — Robson group-specific cesarean delivery rates by year, percent change in cesarean delivery rates, and p-value for linear trend over the study period in British Columbia. (DOCX) [file pmed.1004077.s006.docx]

S4 Table. Cesarean delivery rate by year of delivery and Robson Group, British Columbia, Canada, 2004-2016

| Robson Group | 2004 | 2005 | 2006 | 2007 | 2008 | 2009 | 2010 | 2011 | 2012 | 2013 | 2014 | 2015 | 2016 | % change* | P-value† |
| --- | --- | --- | --- | --- | --- | --- | --- | --- | --- | --- | --- | --- | --- | --- | --- |
| 1 | 19.7 | 19.3 | 19.3 | 19.9 | 19.2 | 19.8 | 19.7 | 20.8 | 20.8 | 21.0 | 21.5 | 21.9 | 22.6 | 15.18 | <0.001 |
| 2a | 37.5 | 36.4 | 36.5 | 37.9 | 37.9 | 36.2 | 39.4 | 41.0 | 41.7 | 41.4 | 41.9 | 44.0 | 45.4 | 21.08 | <0.001 |
| 2b | 100.0 | 100.0 | 100.0 | 100.0 | 100.0 | 100.0 | 100.0 | 100.0 | 100.0 | 100.0 | 100.0 | 100.0 | 100.0 | 0.00 | NA^‡^ |
| 3 | 2.7 | 2.5 | 2.4 | 2.6 | 2.8 | 2.5 | 2.6 | 2.3 | 2.2 | 2.9 | 2.8 | 2.6 | 2.9 | 9.36 | 0.1 |
| 4a | 6.9 | 6.2 | 6.5 | 5.5 | 7.1 | 6.5 | 6.3 | 7.1 | 6.8 | 6.8 | 8.4 | 8.4 | 8.2 | 20.10 | <0.001 |
| 4b | 100.0 | 100.0 | 100.0 | 100.0 | 100.0 | 100.0 | 100.0 | 100.0 | 100.0 | 100.0 | 100.0 | 100.0 | 100.0 | 0.00 | NA^‡^ |
| 5 | 81.7 | 82.2 | 83.2 | 82.8 | 80.5 | 82.0 | 81.2 | 81.5 | 79.9 | 80.5 | 80.8 | 80.2 | 81.3 | -0.46 | <0.001 |
| 6 | 96.5 | 96.4 | 94.8 | 96.0 | 94.4 | 95.8 | 95.8 | 94.4 | 94.9 | 95.0 | 95.9 | 95.2 | 96.4 | -0.08 | 0.6 |
| 7 | 92.6 | 91.8 | 89.8 | 89.3 | 88.8 | 90.3 | 88.3 | 89.2 | 90.2 | 89.0 | 90.3 | 91.6 | 91.4 | -1.37 | 0.8 |
| 8 | 68.5 | 65.7 | 65.5 | 68.6 | 66.6 | 70.2 | 71.0 | 69.6 | 72.0 | 71.8 | 71.4 | 72.0 | 70.7 | 3.09 | <0.001 |
| 9 | 95.7 | 92.9 | 93.2 | 97.0 | 95.9 | 94.9 | 97.0 | 97.9 | 95.6 | 94.3 | 91.6 | 91.0 | 95.5 | -0.21 | 0.2 |
| 10 | 26.7 | 28.0 | 27.7 | 27.8 | 26.7 | 26.3 | 29.3 | 30.5 | 33.2 | 32.6 | 34.5 | 35.9 | 37.6 | 40.88 | <0.001 |
| Unknown | 82.1 | 79.6 | 81.1 | 77.4 | 84.0 | 86.1 | 85.8 | 71.7 | 82.9 | 82.3 | 83.0 | 61.2 | 66.9 | -18.46 | 0.003 |
| All groups | 29.4 | 29.8 | 29.9 | 30.8 | 30.4 | 30.4 | 31.0 | 31.0 | 31.5 | 31.8 | 32.5 | 32.8 | 34.0 | 15.42 | <0.001 |

*Percent change in cesarean delivery rate in 2016 vs 2004.

†P-value of Cochran-Armitage test for linear trend in cesarean delivery rate by year of delivery.

^‡^Groups 2b and 4b are restricted to women with a cesarean delivery.
