# Supplementary material for: Crude and adjusted comparisons of cesarean delivery rates using the Robson classification: A population-based cohort study in Canada and Sweden, 2004 to 2016
Source: PLoS Med. 2022 Aug 1;19(8):e1004077. doi: 10.1371/journal.pmed.1004077 (PMC9377587; doi:10.1371/journal.pmed.1004077)
Supplement: S5 Table — Distribution of determinants of cesarean delivery in Robson Group 1. (DOCX) [file pmed.1004077.s007.docx]

S5 Table. Maternal, obstetric practice, and fetal/infant characteristics in deliveries among women in **Robson group 1**, Sweden and British Columbia, Canada, 2004-2016

| Maternal, obstetric practice or fetal/infant characteristic | Sweden (N=431199)  No. (%) | British Columbia (N=151106)  No. (%) | Standardized difference* |
| --- | --- | --- | --- |
| Maternal age (year) |  |  | 0.38 |
| <20 | 13839 (3.2) | 5614 (3.7) |  |
| 20-24 | 91975 (21.3) | 24628 (16.3) |  |
| 25-29 | 161070 (37.4) | 44005 (29.1) |  |
| 30-34 | 122605 (28.4) | 50717 (33.6) |  |
| 35-39 | 36386 (8.4) | 22217 (14.7) |  |
| 40-44 | 5162 (1.2) | 3747 (2.5) |  |
| ≥45 | 162 (0.0) | 178 (0.1) |  |
| Maternal body mass index (kg/m^2^) |  |  | 0.58 |
| Underweight (<18.5) | 11402 (2.6) | 8219 (5.4) |  |
| Normal weight (18.5-24.9) | 262959 (61.0) | 76615 (50.7) |  |
| Overweight (25.0-29.9) | 89246 (20.7) | 20383 (13.5) |  |
| Obese class I (30.0-34.9) | 24948 (5.8) | 6051 (4.0) |  |
| Obese class II (35.0-39.9) | 6981 (1.6) | 1934 (1.3) |  |
| Obese class III (≥40.0) | 2278 (0.5) | 847 (0.6) |  |
| Missing | 33385 (7.7) | 37057 (24.5) |  |
| Smoking during pregnancy | 28637 (6.6) | 12580 (8.3) | 0.06 |
| Pre-existing diabetes | 642 (0.1) | 156 (0.1) | -0.01 |
| Preeclampsia/eclampsia | 4740 (1.1) | 666 (0.4) | -0.07 |
| Chronic hypertension | 1692 (0.4) | 272 (0.2) | -0.04 |
| In-vitro fertilization | 15113 (3.5) | 2363 (1.6) | -0.12 |
| Post-term delivery (≥42 completed weeks) | 21205 (4.9) | 1885 (1.2) | 0.24 |
| Epidural anaesthesia | 208952 (48.5) | 70976 (47.0) | -0.03 |
| Vacuum | 58782 (13.6) | 19116 (12.7) | -0.03 |
| Forceps | 1471 (0.3) | 9484 (6.3) | 0.34 |
| Infant birth weight (g) |  |  | 0.21 |
| <2500 | 3644 (0.8) | 1666 (1.1) |  |
| 2500-2999 | 47651 (11.1) | 22588 (14.9) |  |
| 3000-3499 | 164626 (38.2) | 64558 (42.7) |  |
| 3500-3999 | 154568 (35.8) | 47969 (31.7) |  |
| 4000-4499 | 51725 (12.0) | 12617 (8.3) |  |
| ≥4500 | 8649 (2.0) | 1668 (1.1) |  |
| Missing | 336 (0.1) | 40 (0.0) |  |
| Infant head circumference at birth (cm) |  |  | 0.16 |
| <33 | 20032 (4.6) | 10651 (7.0) |  |
| 33-34 | 151191 (35.1) | 60908 (40.3) |  |
| 35-36 | 200692 (46.5) | 65280 (43.2) |  |
| ≥37 | 51283 (11.9) | 13177 (8.7) |  |
| Missing | 8001 (1.9) | 1090 (0.7) |  |
| Fetal head in occiput posterior position at delivery | 21301 (4.9) | 12984 (8.6) | 0.15 |
| Congenital anomaly | 13289 (3.1) | 6395 (4.2) | 0.06 |

*Standardized difference values > 0.1 are considered indicative of an imbalance between groups.
