# Supplementary material for: Crude and adjusted comparisons of cesarean delivery rates using the Robson classification: A population-based cohort study in Canada and Sweden, 2004 to 2016
Source: PLoS Med. 2022 Aug 1;19(8):e1004077. doi: 10.1371/journal.pmed.1004077 (PMC9377587; doi:10.1371/journal.pmed.1004077)
Supplement: S6 Table — Distribution of determinants of cesarean delivery in Robson Group 2a. (DOCX) [file pmed.1004077.s008.docx]

S6 Table. Maternal, obstetric practice, and fetal/infant characteristics in deliveries among women in **Robson group 2a**, Sweden and British Columbia, Canada, 2004-2016

| Maternal, obstetric practice or fetal/infant characteristic | Sweden (N=98076)  No. (%) | British Columbia (N=61320)  No. (%) | Standardized difference* |
| --- | --- | --- | --- |
| Maternal age (year) |  |  | 0.18 |
| <20 | 2226 (2.3) | 1632 (2.7) |  |
| 20-24 | 16800 (17.1) | 8802 (14.4) |  |
| 25-29 | 32342 (33.0) | 17067 (27.8) |  |
| 30-34 | 30147 (30.7) | 20211 (33.0) |  |
| 35-39 | 12934 (13.2) | 10501 (17.1) |  |
| 40-44 | 3446 (3.5) | 2859 (4.7) |  |
| ≥45 | 181 (0.2) | 248 (0.4) |  |
| Maternal body mass index (kg/m^2^) |  |  | 0.48 |
| Underweight (<18.5) | 1699 (1.7) | 2244 (3.7) |  |
| Normal weight (18.5-24.9) | 49264 (50.2) | 26461 (43.2) |  |
| Overweight (25.0-29.9) | 25284 (25.8) | 10783 (17.6) |  |
| Obese class I (30.0-34.9) | 9778 (10.0) | 4643 (7.6) |  |
| Obese class II (35.0-39.9) | 3560 (3.6) | 2104 (3.4) |  |
| Obese class III (≥40.0) | 1393 (1.4) | 1213 (2.0) |  |
| Missing | 7098 (7.2) | 13872 (22.6) |  |
| Smoking during pregnancy | 6331 (6.5) | 5304 (8.6) | 0.01 |
| Pre-existing diabetes | 1115 (1.1) | 557 (0.9) | -0.02 |
| Preeclampsia/eclampsia | 13586 (13.9) | 2514 (4.1) | -0.35 |
| Chronic hypertension | 1223 (1.2) | 791 (1.3) | 0.00 |
| In-vitro fertilization | 5715 (5.8) | 1752 (2.9) | -0.15 |
| Post-term delivery (≥42 completed weeks) | 30832 (31.4) | 1841 (3.0) | 0.80 |
| Epidural anaesthesia | 64845 (66.1) | 38257 (62.4) | -0.10 |
| Vacuum | 15684 (16.0) | 7159 (11.7) | -0.13 |
| Forceps | 323 (0.3) | 3918 (6.4) | 0.34 |
| Infant birth weight (g) |  |  | 0.17 |
| <2500 | 2518 (2.6) | 1493 (2.4) |  |
| 2500-2999 | 11783 (12.0) | 8613 (14.0) |  |
| 3000-3499 | 28943 (29.5) | 21668 (35.3) |  |
| 3500-3999 | 33089 (33.7) | 20410 (33.3) |  |
| 4000-4499 | 16928 (17.3) | 7672 (12.5) |  |
| ≥4500 | 4691 (4.8) | 1443 (2.4) |  |
| Missing | 124 (0.1) | 21 (0.0) |  |
| Infant head circumference at birth (cm) |  |  | 0.20 |
| <33 | 4061 (4.1) | 3144 (5.1) |  |
| 33-34 | 25589 (26.1) | 19334 (31.5) |  |
| 35-36 | 45753 (46.7) | 28996 (47.3) |  |
| ≥37 | 20600 (21.0) | 9360 (15.3) |  |
| Missing | 2073 (2.1) | 486 (0.8) |  |
| Fetal head in occiput posterior position at delivery | 6054 (6.2) | 6455 (10.5) | 0.16 |
| Congenital anomaly | 3813 (3.9) | 3315 (5.4) | 0.07 |

*Standardized difference values > 0.1 are considered indicative of an imbalance between groups.
