# Supplementary material for: Crude and adjusted comparisons of cesarean delivery rates using the Robson classification: A population-based cohort study in Canada and Sweden, 2004 to 2016
Source: PLoS Med. 2022 Aug 1;19(8):e1004077. doi: 10.1371/journal.pmed.1004077 (PMC9377587; doi:10.1371/journal.pmed.1004077)
Supplement: S7 Table — Distribution of determinants of cesarean delivery in Robson Group 2b. (DOCX) [file pmed.1004077.s009.docx]

S7 Table. Maternal, obstetric practice, and fetal/infant characteristics in deliveries among women in **Robson group 2b**, Sweden and British Columbia, Canada, 2004-2016

| Maternal, obstetric practice or fetal/infant characteristic | Sweden (N=15851)  No. (%) | British Columbia (N=6764)  No. (%) | Standardized difference* |
| --- | --- | --- | --- |
| Maternal age (year) |  |  | 0.39 |
| <20 | 264 (1.7) | 72 (1.1) |  |
| 20-24 | 1828 (11.5) | 505 (7.5) |  |
| 25-29 | 3901 (24.6) | 1250 (18.5) |  |
| 30-34 | 4980 (31.4) | 2113 (31.2) |  |
| 35-39 | 3409 (21.5) | 1839 (27.2) |  |
| 40-44 | 1271 (8.0) | 851 (12.6) |  |
| ≥45 | 198 (1.2) | 134 (2.0) |  |
| Maternal body mass index (kg/m^2^) |  |  | 0.63 |
| Underweight (<18.5) | 419 (2.6) | 220 (3.3) |  |
| Normal weight (18.5-24.9) | 8628 (54.4) | 2652 (39.2) |  |
| Overweight (25.0-29.9) | 3574 (22.5) | 1000 (14.8) |  |
| Obese class I (30.0-34.9) | 1222 (7.7) | 447 (6.6) |  |
| Obese class II (35.0-39.9) | 425 (2.7) | 214 (3.2) |  |
| Obese class III (≥40.0) | 170 (1.1) | 171 (2.5) |  |
| Missing | 1413 (8.9) | 2060 (30.5) |  |
| Smoking during pregnancy | 1132 (7.1) | 451 (6.7) | -0.02 |
| Pre-existing diabetes | 237 (1.5) | 93 (1.4) | -0.01 |
| Preeclampsia/eclampsia | 922 (5.8) | 197 (2.9) | -0.14 |
| Chronic hypertension | 204 (1.3) | 92 (1.4) | 0.01 |
| In-vitro fertilization | 1600 (10.1) | 463 (6.8) | -0.12 |
| Post-term delivery (≥42 completed weeks) | 422 (2.7) | 66 (1.0) | 0.14 |
| Epidural anaesthesia | 426 (2.7) | 320 (4.7) | 0.11 |
| Infant birth weight (g) |  |  | 0.15 |
| <2500 | 596 (3.8) | 236 (3.5) |  |
| 2500-2999 | 2561 (16.2) | 1110 (16.4) |  |
| 3000-3499 | 6373 (40.2) | 2555 (37.8) |  |
| 3500-3999 | 4214 (26.6) | 1811 (26.8) |  |
| 4000-4499 | 1294 (8.2) | 781 (11.5) |  |
| ≥4500 | 782 (4.9) | 268 (4.0) |  |
| Missing | 31 (0.2) | <5 (<0.1) |  |
| Infant head circumference at birth (cm) |  |  | 0.19 |
| <33 | 518 (3.3) | 212 (3.1) |  |
| 33-34 | 4617 (29.1) | 1785 (26.4) |  |
| 35-36 | 7724 (48.7) | 3281 (48.5) |  |
| ≥37 | 2576 (16.3) | 1428 (21.1) |  |
| Missing | 416 (2.6) | 58 (0.9) |  |
| Fetal head in occiput posterior position at delivery | 420 (2.6) | 221 (3.3) | 0.04 |
| Congenital anomaly | 794 (5.0) | 469 (6.9) | 0.08 |

*Standardized difference values > 0.1 are considered indicative of an imbalance between groups.
