# Supplementary material for: Crude and adjusted comparisons of cesarean delivery rates using the Robson classification: A population-based cohort study in Canada and Sweden, 2004 to 2016
Source: PLoS Med. 2022 Aug 1;19(8):e1004077. doi: 10.1371/journal.pmed.1004077 (PMC9377587; doi:10.1371/journal.pmed.1004077)
Supplement: S8 Table — Distribution of determinants of cesarean delivery in Robson Group 3. (DOCX) [file pmed.1004077.s010.docx]

S8 Table. Maternal, obstetric practice, and fetal/infant characteristics in deliveries among women in **Robson group 3**, Sweden and British Columbia, Canada, 2004-2016

| Maternal, obstetric practice or fetal/infant characteristic | Sweden (N=500236)  No. (%) | British Columbia (N=151071)  No. (%) | Standardized difference* |
| --- | --- | --- | --- |
| Maternal age (year) |  |  | 0.06 |
| <20 | 1316 (0.3) | 525 (0.3) |  |
| 20-24 | 38938 (7.8) | 11345 (7.5) |  |
| 25-29 | 132834 (26.6) | 35054 (23.2) |  |
| 30-34 | 197870 (39.6) | 55537 (36.8) |  |
| 35-39 | 108818 (21.8) | 39196 (25.9) |  |
| 40-44 | 19730 (3.9) | 8919 (5.9) |  |
| ≥45 | 730 (0.1) | 495 (0.3) |  |
| Maternal body mass index (kg/m^2^) |  |  | 0.66 |
| Underweight (<18.5) | 9330 (1.9) | 5558 (3.7) |  |
| Normal weight (18.5-24.9) | 274634 (54.9) | 65147 (43.1) |  |
| Overweight (25.0-29.9) | 123945 (24.8) | 22125 (14.6) |  |
| Obese class I (30.0-34.9) | 39543 (7.9) | 7638 (5.1) |  |
| Obese class II (35.0-39.9) | 11459 (2.3) | 2673 (1.8) |  |
| Obese class III (≥40.0) | 3689 (0.7) | 1127 (0.7) |  |
| Missing | 37636 (7.5) | 46803 (31.0) |  |
| Parity |  |  | 0.09 |
| 1 | 340814 (68.1) | 101895 (67.4) |  |
| 2 | 114241 (22.8) | 33581 (22.2) |  |
| 3-4 | 38386 (7.7) | 13148 (8.7) |  |
| ≥5 | 6795 (1.4) | 2398 (1.6) |  |
| Missing | 0 (0.0) | 49 (0.0) |  |
| Smoking during pregnancy | 33400 (6.7) | 12453 (8.2) | 0.06 |
| Pre-existing diabetes | 657 (0.1) | 145 (0.1) | -0.01 |
| Preeclampsia/eclampsia | 1608 (0.3) | 162 (0.1) | -0.05 |
| Chronic hypertension | 2183 (0.4) | 284 (0.2) | -0.04 |
| In-vitro fertilization | 6127 (1.2) | 1023 (0.7) | -0.06 |
| Post-term delivery (≥42 completed weeks) | 16320 (3.3) | 1326 (0.9) | 0.14 |
| Epidural anaesthesia | 73273 (14.6) | 26646 (17.6) | 0.08 |
| Vacuum | 9500 (1.9) | 6137 (4.1) | 0.13 |
| Forceps | 200 (0.0) | 958 (0.6) | 0.10 |
| Infant birth weight (g) |  |  | 0.34 |
| <2500 | 1978 (0.4) | 1002 (0.7) |  |
| 2500-2999 | 31621 (6.3) | 15471 (10.2) |  |
| 3000-3499 | 149689 (29.9) | 56326 (37.3) |  |
| 3500-3999 | 200244 (40.0) | 55372 (36.7) |  |
| 4000-4499 | 94539 (18.9) | 19549 (12.9) |  |
| ≥4500 | 21849 (4.4) | 3300 (2.2) |  |
| Missing | 316 (0.1) | 51 (0.0) |  |
| Infant head circumference at birth (cm) |  |  | 0.23 |
| <33 | 11228 (2.2) | 6031 (4.0) |  |
| 33-34 | 147297 (29.4) | 54564 (36.1) |  |
| 35-36 | 263233 (52.6) | 73840 (48.9) |  |
| ≥37 | 70528 (14.1) | 15503 (10.3) |  |
| Missing | 7950 (1.6) | 1133 (0.7) |  |
| Fetal head in occiput posterior position at delivery | 19566 (3.9) | 6018 (4.0) | 0.00 |
| Congenital anomaly | 14321 (2.9) | 4956 (3.3) | 0.02 |

*Standardized difference values > 0.1 are considered indicative of an imbalance between groups.
