# Supplementary material for: Crude and adjusted comparisons of cesarean delivery rates using the Robson classification: A population-based cohort study in Canada and Sweden, 2004 to 2016
Source: PLoS Med. 2022 Aug 1;19(8):e1004077. doi: 10.1371/journal.pmed.1004077 (PMC9377587; doi:10.1371/journal.pmed.1004077)
Supplement: S9 Table — Distribution of determinants of cesarean delivery in Robson Group 4a. (DOCX) [file pmed.1004077.s011.docx]

S9 Table. Maternal, obstetric practice, and fetal/infant characteristics in deliveries among women in **Robson group 4a**, Sweden and British Columbia, Canada, 2004-2016

| Maternal, obstetric practice or fetal/infant characteristic | Sweden (N=82066)  No. (%) | British Columbia (N=38068)  No. (%) | Standardized difference* |
| --- | --- | --- | --- |
| Maternal age (year) |  |  | 0.09 |
| <20 | 148 (0.2) | 79 (0.2) |  |
| 20-24 | 5269 (6.4) | 2500 (6.6) |  |
| 25-29 | 19219 (23.4) | 8501 (22.3) |  |
| 30-34 | 29969 (36.5) | 13380 (35.1) |  |
| 35-39 | 21117 (25.7) | 9906 (26.0) |  |
| 40-44 | 5983 (7.3) | 3423 (9.0) |  |
| ≥45 | 361 (0.4) | 279 (0.7) |  |
| Maternal body mass index (kg/m^2^) |  |  | 0.58 |
| Underweight (<18.5) | 982 (1.2) | 965 (2.5) |  |
| Normal weight (18.5-24.9) | 35616 (43.4) | 13874 (36.4) |  |
| Overweight (25.0-29.9) | 23320 (28.4) | 6953 (18.3) |  |
| Obese class I (30.0-34.9) | 10438 (12.7) | 3256 (8.6) |  |
| Obese class II (35.0-39.9) | 4025 (4.9) | 1505 (4.0) |  |
| Obese class III (≥40.0) | 1626 (2.0) | 903 (2.4) |  |
| Missing | 6059 (7.4) | 10612 (27.9) |  |
| Parity |  |  | 0.13 |
| 1 | 46237 (56.3) | 23590 (62.0) |  |
| 2 | 23121 (28.2) | 9456 (24.8) |  |
| 3-4 | 10347 (12.6) | 4304 (11.3) |  |
| ≥5 | 2361 (2.9) | 707 (1.9) |  |
| Missing | 0 (0.0) | 11 (0.0) |  |
| Smoking during pregnancy | 6830 (8.3) | 3849 (10.1) | 0.06 |
| Pre-existing diabetes | 1030 (1.3) | 386 (1.0) | -0.02 |
| Preeclampsia/eclampsia | 4701 (5.7) | 657 (1.7) | -0.21 |
| Chronic hypertension | 1349 (1.6) | 501 (1.3) | -0.03 |
| In-vitro fertilization | 1612 (2.0) | 487 (1.3) | -0.05 |
| Post-term delivery (≥42 completed weeks) | 18098 (22.1) | 700 (1.8) | 0.65 |
| Epidural anaesthesia | 25813 (31.5) | 12700 (33.4) | 0.04 |
| Vacuum delivery | 2416 (2.9) | 2291 (6.0) | 0.15 |
| Forceps delivery | 52 (0.1) | 436 (1.1) | 0.14 |
| Infant birth weight (g) |  |  | 0.24 |
| <2500 | 1307 (1.6) | 677 (1.8) |  |
| 2500-2999 | 7277 (8.9) | 4159 (10.9) |  |
| 3000-3499 | 21612 (26.3) | 12358 (32.5) |  |
| 3500-3999 | 27896 (34.0) | 13328 (35.0) |  |
| 4000-4499 | 17477 (21.3) | 6124 (16.1) |  |
| ≥4500 | 6443 (7.9) | 1404 (3.7) |  |
| Missing | 54 (0.1) | 18 (0.0) |  |
| Infant head circumference at birth (cm) |  |  | 0.21 |
| <33 | 1991 (2.4) | 1272 (3.3) |  |
| 33-34 | 18032 (22.0) | 10459 (27.5) |  |
| 35-36 | 40532 (49.4) | 19400 (51.0) |  |
| ≥37 | 20092 (24.5) | 6634 (17.4) |  |
| Missing | 1419 (1.7) | 303 (0.8) |  |
| Fetal head in occiput posterior position at delivery | 3127 (3.8) | 1749 (4.6) | 0.04 |
| Congenital anomaly | 2839 (3.5) | 1746 (4.6) | 0.06 |

*Standardized difference values > 0.1 are considered indicative of an imbalance between groups.
