# Supplementary material for: Crude and adjusted comparisons of cesarean delivery rates using the Robson classification: A population-based cohort study in Canada and Sweden, 2004 to 2016
Source: PLoS Med. 2022 Aug 1;19(8):e1004077. doi: 10.1371/journal.pmed.1004077 (PMC9377587; doi:10.1371/journal.pmed.1004077)
Supplement: S10 Table — Distribution of determinants of cesarean delivery in Robson Group 4b. (DOCX) [file pmed.1004077.s012.docx]

S10 Table. Maternal, obstetric practice, and fetal/infant characteristics in deliveries among women in **Robson group 4b**, Sweden and British Columbia, Canada, 2004-2016

| Maternal, obstetric practice or fetal/infant characteristic | Sweden (N=16835) | British Columbia (N=2672) | Standardized difference* |
| --- | --- | --- | --- |
| Maternal age (year) |  |  | 0.28 |
| <20 | 15 (0.1) | <5 (<0.2) |  |
| 20-24 | 587 (3.5) | 111 (4.2) |  |
| 25-29 | 3267 (19.4) | 397 (14.9) |  |
| 30-34 | 6534 (38.8) | 882 (33.0) |  |
| 35-39 | 5086 (30.2) | 879 (32.9) |  |
| 40-44 | 1253 (7.4) | 363 (13.6) |  |
| ≥45 | 93 (0.6) | 37 (1.4) |  |
| Maternal body mass index (kg/m^2^) |  |  | 0.71 |
| Underweight (<18.5) | 236 (1.4) | 59 (2.2) |  |
| Normal weight (18.5-24.9) | 8709 (51.7) | 936 (35.0) |  |
| Overweight (25.0-29.9) | 4408 (26.2) | 442 (16.5) |  |
| Obese class I (30.0-34.9) | 1462 (8.7) | 188 (7.0) |  |
| Obese class II (35.0-39.9) | 439 (2.6) | 78 (2.9) |  |
| Obese class III (≥40.0) | 153 (0.9) | 50 (1.9) |  |
| Missing | 1428 (8.5) | 919 (34.4) |  |
| Parity |  |  | 0.15 |
| 1 | 12416 (73.8) | 1945 (72.8) |  |
| 2 | 3337 (19.8) | 518 (19.4) |  |
| 3-4 | 953 (5.7) | 183 (6.8) |  |
| ≥5 | 129 (0.8) | 26 (1.0) |  |
| Smoking during pregnancy | 992 (5.9) | 230 (8.6) | 0.10 |
| Pre-existing diabetes | 129 (0.8) | 23 (0.9) | 0.01 |
| Preeclampsia/eclampsia | 186 (1.1) | 22 (0.8) | -0.03 |
| Chronic hypertension | 147 (0.9) | 28 (1.0) | 0.02 |
| In-vitro fertilization | 475 (2.8) | 68 (2.5) | -0.02 |
| Post-term delivery (≥42 completed weeks) | 131 (0.8) | 16 (0.6) | 0.0 |
| Epidural anaesthesia | 532 (3.2) | 99 (3.7) | 0.03 |
| Infant birth weight (g) |  |  |  |
| <2500 | 202 (1.2) | 57 (2.1) | 0.08 |
| 2500-2999 | 1443 (8.6) | 322 (12.1) |  |
| 3000-3499 | 5996 (35.6) | 1001 (37.5) |  |
| 3500-3999 | 6201 (36.8) | 825 (30.9) |  |
| 4000-4499 | 2178 (12.9) | 337 (12.6) |  |
| ≥4500 | 788 (4.7) | 129 (4.8) |  |
| Missing | 27 (0.2) | <5 (<0.2) |  |
| Infant head circumference at birth (cm) |  |  | 0.30 |
| <33 | 212 (1.3) | 73 (2.7) |  |
| 33-34 | 3429 (20.4) | 608 (22.8) |  |
| 35-36 | 9219 (54.8) | 1392 (52.1) |  |
| ≥37 | 3584 (21.3) | 580 (21.7) |  |
| Missing | 391 (2.3) | 19 (0.7) |  |
| Fetal head in occiput posterior position at delivery | 367 (2.2) | 60 (2.2) | 0.00 |
| Congenital anomaly | 733 (4.4) | 154 (5.8) | 0.06 |

*Standardized difference values > 0.1 are considered indicative of an imbalance between groups.
