# Supplementary material for: Crude and adjusted comparisons of cesarean delivery rates using the Robson classification: A population-based cohort study in Canada and Sweden, 2004 to 2016
Source: PLoS Med. 2022 Aug 1;19(8):e1004077. doi: 10.1371/journal.pmed.1004077 (PMC9377587; doi:10.1371/journal.pmed.1004077)
Supplement: S11 Table — Distribution of determinants of cesarean delivery in Robson Group 5. (DOCX) [file pmed.1004077.s013.docx]

S11 Table. Maternal, obstetric practice, and fetal/infant characteristics in deliveries among women in **Robson group 5**, Sweden and British Columbia, Canada, 2004-2016

| Maternal, obstetric practice or fetal/infant characteristic | Sweden (N=120104)  No. (%) | British Columbia (N=71665)  No. (%) | Standardized difference* |
| --- | --- | --- | --- |
| Maternal age (year) |  |  | 0.20 |
| <20 | 169 (0.1) | 84 (0.1) |  |
| 20-24 | 5378 (4.5) | 3017 (4.2) |  |
| 25-29 | 23888 (19.9) | 12683 (17.7) |  |
| 30-34 | 46763 (38.9) | 25783 (36.0) |  |
| 35-39 | 35060 (29.2) | 22767 (31.8) |  |
| 40-44 | 8439 (7.0) | 6860 (9.6) |  |
| ≥45 | 407 (0.3) | 471 (0.7) |  |
| Maternal body mass index (kg/m^2^) |  |  | 0.70 |
| Underweight (<18.5) | 1440 (1.2) | 1561 (2.2) |  |
| Normal weight (18.5-24.9) | 54514 (45.4) | 24932 (34.8) |  |
| Overweight (25.0-29.9) | 33870 (28.2) | 12215 (17.0) |  |
| Obese class I (30.0-34.9) | 14179 (11.8) | 5351 (7.5) |  |
| Obese class II (35.0-39.9) | 4978 (4.1) | 2344 (3.3) |  |
| Obese class III (≥40.0) | 2045 (1.7) | 1462 (2.0) |  |
| Missing | 9078 (7.6) | 23800 (33.2) |  |
| Parity |  |  | 0.25 |
| 1 | 75879 (63.2) | 51238 (71.5) |  |
| 2 | 30517 (25.4) | 15103 (21.1) |  |
| 3-4 | 11611 (9.7) | 4567 (6.4) |  |
| ≥5 | 2097 (1.7) | 724 (1.0) |  |
| Missing | 0 (0.0) | 33 (0.0) |  |
| Smoking during pregnancy | 7943 (6.6) | 5043 (7.0) | 0.02 |
| Pre-existing diabetes | 1247 (1.0) | 530 (0.7) | -0.03 |
| Preeclampsia/eclampsia | 2380 (2.0) | 443 (0.6) | -0.12 |
| Chronic hypertension | 1356 (1.1) | 565 (0.8) | -0.03 |
| In-vitro fertilization | 2284 (1.9) | 886 (1.2) | -0.05 |
| Onset of labour |  |  | 0.63 |
| Spontaneous | 59580 (49.6) | 23188 (32.4) |  |
| Induced | 16036 (13.4) | 2781 (3.9) |  |
| Cesarean delivery before labour | 42926 (35.7) | 45693 (63.8) |  |
| Unknown | 1562 (1.3) | <5 (<0.0) |  |
| Post-term delivery (≥42 completed weeks) | 6952 (5.8) | 442 (0.6) | 0.31 |
| Epidural anaesthesia | 32913 (27.4) | 8467 (11.8) | -0.40 |
| Vacuum | 7642 (6.4) | 1892 (2.6) | -0.18 |
| Forceps | 183 (0.2) | 786 (1.1) | 0.12 |
| Infant birth weight (g) |  |  | 0.31 |
| <2500 | 1124 (0.9) | 918 (1.3) |  |
| 2500-2999 | 10103 (8.4) | 8401 (11.7) |  |
| 3000-3499 | 37826 (31.5) | 28051 (39.1) |  |
| 3500-3999 | 43810 (36.5) | 24619 (34.4) |  |
| 4000-4499 | 20969 (17.5) | 8068 (11.3) |  |
| ≥4500 | 6137 (5.1) | 1590 (2.2) |  |
| Missing | 135 (0.1) | 18 (0.0) |  |
| Infant head circumference at birth (cm) |  |  | 0.24 |
| <33 | 2487 (2.1) | 1844 (2.6) |  |
| 33-34 | 28372 (23.6) | 18378 (25.6) |  |
| 35-36 | 61755 (51.4) | 38503 (53.7) |  |
| ≥37 | 25299 (21.1) | 12495 (17.4) |  |
| Missing | 2191 (1.8) | 445 (0.6) |  |
| Fetal head in occiput posterior position at delivery | 5143 (4.3) | 2721 (3.8) | -0.02 |
| Congenital anomaly | 3866 (3.2) | 3242 (4.5) | 0.07 |

*Standardized difference values > 0.1 are considered indicative of an imbalance between groups.
