# Supplementary material for: Crude and adjusted comparisons of cesarean delivery rates using the Robson classification: A population-based cohort study in Canada and Sweden, 2004 to 2016
Source: PLoS Med. 2022 Aug 1;19(8):e1004077. doi: 10.1371/journal.pmed.1004077 (PMC9377587; doi:10.1371/journal.pmed.1004077)
Supplement: S12 Table — Distribution of determinants of cesarean delivery in Robson Group 6. (DOCX) [file pmed.1004077.s014.docx]

S12 Table. Distribution of maternal, obstetric practice, and fetal/infant characteristics in deliveries among women in **Robson group 6**, Sweden and British Columbia, Canada, 2004-2016

| Maternal, obstetric practice or fetal/infant characteristic | Sweden (N=27503)  No. (%) | British Columbia (N=12932)  No. (%) | Standardized difference* |
| --- | --- | --- | --- |
| Maternal age (year) |  |  |  |
| <20 | 411 (1.5) | 213 (1.6) | 0.33 |
| 20-24 | 3767 (13.7) | 1328 (10.3) |  |
| 25-29 | 9348 (34.0) | 3179 (24.6) |  |
| 30-34 | 9473 (34.4) | 4684 (36.2) |  |
| 35-39 | 3715 (13.5) | 2739 (21.2) |  |
| 40-44 | 740 (2.7) | 709 (5.5) |  |
| ≥45 | 49 (0.2) | 80 (0.6) |  |
| Maternal body mass index (kg/m^2^) |  |  | 0.54 |
| Underweight (<18.5) | 658 (2.4) | 592 (4.6) |  |
| Normal weight (18.5-24.9) | 16104 (58.6) | 5996 (46.4) |  |
| Overweight (25.0-29.9) | 5779 (21.0) | 1830 (14.2) |  |
| Obese class I (30.0-34.9) | 1716 (6.2) | 625 (4.8) |  |
| Obese class II (35.0-39.9) | 505 (1.8) | 244 (1.9) |  |
| Obese class III (≥40.0) | 216 (0.8) | 138 (1.1) |  |
| Missing | 2525 (9.2) | 3507 (27.1) |  |
| Smoking during pregnancy | 1641 (6.0) | 976 (7.5) | 0.06 |
| Pre-existing diabetes | 161 (0.6) | 95 (0.7) | 0.02 |
| Preeclampsia/eclampsia | 1185 (4.3) | 343 (2.7) | -0.09 |
| Chronic hypertension | 221 (0.8) | 131 (1.0) | 0.02 |
| In-vitro fertilization | 1510 (5.5) | 426 (3.3) | -0.11 |
| Onset of labour |  |  | 0.23 |
| Spontaneous | 8657 (31.5) | 3865 (29.9) |  |
| Induced | 535 (1.9) | 533 (4.1) |  |
| Cesarean delivery before labour | 17874 (65.0) | 8534 (66.0) |  |
| Unknown | 437 (1.6) | 0 (0.0) |  |
| Gestational age  (completed weeks) |  |  | 0.22 |
| Very early preterm (22-27) | 841 (3.1) | 341 (2.6) |  |
| Early preterm (28-31) | 796 (2.9) | 322 (2.5) |  |
| Late preterm (32-36) | 3385 (12.3) | 1846 (14.3) |  |
| Term (37-41) | 22197 (80.7) | 10348 (80.0) |  |
| Post-term (≥42) | 279 (1.0) | 59 (0.5) |  |
| Missing | 5 (0.0) | 16 (0.1) |  |
| Epidural anaesthesia | 1392 (5.1) | 825 (6.4) | 0.06 |
| Vacuum | 9 (0.0) | 0 (0.0) | -0.03 |
| Forceps | 25 (0.1) | 36 (0.3) | 0.04 |
| Infant birth weight (g) |  |  | 0.04 |
| <2500 | 4006 (14.6) | 1749 (13.5) |  |
| 2500-2999 | 5699 (20.7) | 2884 (22.3) |  |
| 3000-3499 | 10209 (37.1) | 4875 (37.7) |  |
| 3500-3999 | 5941 (21.6) | 2704 (20.9) |  |
| 4000-4499 | 1379 (5.0) | 602 (4.7) |  |
| ≥4500 | 226 (0.8) | 87 (0.7) |  |
| Missing | 43 (0.2) | 31 (0.2) |  |
| Infant head circumference at birth (cm) |  |  | 0.17 |
| <33 | 2980 (10.8) | 1321 (10.2) |  |
| 33-34 | 5896 (21.4) | 2958 (22.9) |  |
| 35-36 | 11939 (43.4) | 5769 (44.6) |  |
| ≥37 | 5636 (20.5) | 2629 (20.3) |  |
| Missing | 1052 (3.8) | 255 (2.0) |  |
| Congenital anomaly | 2578 (9.4) | 2094 (16.2) | 0.21 |

*Standardized difference values > 0.1 are considered indicative of an imbalance between groups.
