# Supplementary material for: Crude and adjusted comparisons of cesarean delivery rates using the Robson classification: A population-based cohort study in Canada and Sweden, 2004 to 2016
Source: PLoS Med. 2022 Aug 1;19(8):e1004077. doi: 10.1371/journal.pmed.1004077 (PMC9377587; doi:10.1371/journal.pmed.1004077)
Supplement: S13 Table — Distribution of determinants of cesarean delivery in Robson Group 7. (DOCX) [file pmed.1004077.s015.docx]

S13 Table. Maternal, obstetric practice, and fetal/infant characteristics in deliveries among women in **Robson group 7**, Sweden and British Columbia, Canada, 2004-2016

| Maternal, obstetric practice or fetal/infant characteristic | Sweden (N=16572)  No. (%) | British Columbia (N=8771)  No. (%) | Standardized difference* |
| --- | --- | --- | --- |
| Maternal age (year) |  |  | 0.21 |
| <20 | 37 (0.2) | 9 (0.1) |  |
| 20-24 | 787 (4.7) | 408 (4.7) |  |
| 25-29 | 3337 (20.1) | 1512 (17.2) |  |
| 30-34 | 6262 (37.8) | 2998 (34.2) |  |
| 35-39 | 4786 (28.9) | 2795 (31.9) |  |
| 40-44 | 1293 (7.8) | 948 (10.8) |  |
| ≥45 | 70 (0.4) | 101 (1.2) |  |
| Maternal body mass index (kg/m^2^) |  |  | 0.66 |
| Underweight (<18.5) | 325 (2.0) | 288 (3.3) |  |
| Normal weight (18.5-24.9) | 7962 (48.0) | 3171 (36.2) |  |
| Overweight (25.0-29.9) | 4206 (25.4) | 1318 (15.0) |  |
| Obese class I (30.0-34.9) | 1633 (9.9) | 583 (6.6) |  |
| Obese class II (35.0-39.9) | 634 (3.8) | 285 (3.2) |  |
| Obese class III (≥40.0) | 274 (1.7) | 184 (2.1) |  |
| Missing | 1538 (9.3) | 2942 (33.5) |  |
| Parity |  |  | 0.07 |
| 1 | 11140 (67.2) | 6128 (69.9) |  |
| 2 | 3686 (22.2) | 1780 (20.3) |  |
| 3-4 | 1472 (8.9) | 708 (8.1) |  |
| ≥5 | 274 (1.7) | 147 (1.7) |  |
| Smoking during pregnancy | 1304 (7.9) | 840 (9.6) | 0.06 |
| Pre-existing diabetes | 154 (0.9) | 90 (1.0) | 0.01 |
| Preeclampsia/eclampsia | 494 (3.0) | 131 (1.5) | -0.10 |
| Chronic hypertension | 236 (1.4) | 118 (1.3) | -0.01 |
| In-vitro fertilization | 358 (2.2) | 114 (1.3) | -0.07 |
| Onset of labour |  |  | 0.21 |
| Spontaneous | 5498 (33.2) | 3107 (35.4) |  |
| Induced | 623 (3.8) | 399 (4.5) |  |
| Cesarean delivery before labour | 10161 (61.3) | 5264 (60.0) |  |
| Unknown | 290 (1.7) | <5 (<0.1) |  |
| Gestational age (completed weeks) |  |  | 0.17 |
| Very early preterm (22-27) | 657 (4.0) | 412 (4.7) |  |
| Early preterm (28-31) | 730 (4.4) | 362 (4.1) |  |
| Late preterm (32-36) | 2311 (13.9) | 1469 (16.7) |  |
| Term (37-41) | 12727 (76.8) | 6482 (73.9) |  |
| Post-term (≥42) | 140 (0.8) | 31 (0.4) |  |
| Missing | 7 (0.0) | 15 (0.2) |  |
| Epidural anaesthesia | 762 (4.6) | 505 (5.8) | 0.05 |
| Infant birth weight (g) |  |  | 0.17 |
| <2500 | 2825 (17.0) | 1467 (16.7) |  |
| 2500-2999 | 2717 (16.4) | 1630 (18.6) |  |
| 3000-3499 | 5410 (32.6) | 2997 (34.2) |  |
| 3500-3999 | 3849 (23.2) | 1912 (21.8) |  |
| 4000-4499 | 1389 (8.4) | 605 (6.9) |  |
| ≥4500 | 333 (2.0) | 138 (1.6) |  |
| Missing | 49 (0.3) | 22 (0.3) |  |
| Infant head circumference at birth (cm) |  |  | 0.17 |
| <33 | 2146 (12.9) | 1127 (12.8) |  |
| 33-34 | 3584 (21.6) | 2072 (23.6) |  |
| 35-36 | 6916 (41.7) | 3761 (42.9) |  |
| ≥37 | 3049 (18.4) | 1545 (17.6) |  |
| Missing | 877 (5.3) | 266 (3.0) |  |
| Congenital anomaly | 1223 (7.4) | 1030 (11.7) | 0.15 |

*Standardized difference values > 0.1 are considered indicative of an imbalance between groups.
