# Supplementary material for: Crude and adjusted comparisons of cesarean delivery rates using the Robson classification: A population-based cohort study in Canada and Sweden, 2004 to 2016
Source: PLoS Med. 2022 Aug 1;19(8):e1004077. doi: 10.1371/journal.pmed.1004077 (PMC9377587; doi:10.1371/journal.pmed.1004077)
Supplement: S14 Table — Distribution of determinants of cesarean delivery in Robson Group 8. (DOCX) [file pmed.1004077.s016.docx]

S14 Table. Maternal, obstetric practice, and fetal/infant characteristics in deliveries among women in **Robson group 8**, Sweden and British Columbia, Canada, 2004-2016

| Maternal, obstetric practice or fetal/infant characteristic | Sweden (N=19701)  No. (%) | British Columbia (N=8972)  No. (%) | Standardized difference* |
| --- | --- | --- | --- |
| Maternal age (year) |  |  | 0.28 |
| <20 | 129 (0.7) | 51 (0.6) |  |
| 20-24 | 1583 (8.0) | 568 (6.3) |  |
| 25-29 | 4924 (25.0) | 1649 (18.4) |  |
| 30-34 | 7118 (36.1) | 2939 (32.8) |  |
| 35-39 | 4586 (23.3) | 2682 (29.9) |  |
| 40-44 | 1142 (5.8) | 901 (10.0) |  |
| ≥45 | 219 (1.1) | 182 (2.0) |  |
| Maternal body mass index (kg/m^2^) |  |  | 0.58 |
| Underweight (<18.5) | 285 (1.4) | 261 (2.9) |  |
| Normal weight (18.5-24.9) | 10000 (50.8) | 3681 (41.0) |  |
| Overweight (25.0-29.9) | 4988 (25.3) | 1461 (16.3) |  |
| Obese class I (30.0-34.9) | 1699 (8.6) | 544 (6.1) |  |
| Obese class II (35.0-39.9) | 552 (2.8) | 209 (2.3) |  |
| Obese class III (≥40.0) | 202 (1.0) | 123 (1.4) |  |
| Missing | 1975 (10.0) | 2693 (30.0) |  |
| Parity |  |  | 0.21 |
| 0 | 8694 (44.1) | 4607 (51.3) |  |
| 1 | 7077 (35.9) | 2801 (31.2) |  |
| 2 | 2671 (13.6) | 1010 (11.3) |  |
| 3-4 | 1060 (5.4) | 477 (5.3) |  |
| ≥5 | 199 (1.0) | 75 (0.8) |  |
| Smoking during pregnancy | 1226 (6.2) | 658 (7.3) | 0.04 |
| Pre-existing diabetes | 67 (0.3) | 58 (0.6) | 0.04 |
| Preeclampsia/eclampsia | 2285 (11.6) | 659 (7.3) | -0.15 |
| Chronic hypertension | 148 (0.8) | 119 (1.3) | 0.06 |
| In-vitro fertilization | 2360 (12.0) | 1879 (20.9) | 0.24 |
| Onset of labour |  |  | 0.21 |
| Spontaneous | 8198 (41.6) | 3198 (35.6) |  |
| Induced | 5170 (26.2) | 2042 (22.8) |  |
| Cesarean delivery before labour | 6027 (30.6) | 3731 (41.6) |  |
| Unknown | 306 (1.6) | <5 (<0.1) |  |
| Gestational age (completed weeks) |  |  | 0.48 |
| Very early preterm (22-27) | 597 (3.0) | 294 (3.3) |  |
| Early preterm (28-31) | 1194 (6.1) | 640 (7.1) |  |
| Late preterm (32-36) | 6733 (34.2) | 4850 (54.1) |  |
| Term (37-41) | 11150 (56.6) | 3170 (35.3) |  |
| Post-term (≥42) | 15 (0.1) | 12 (0.1) |  |
| Missing | 12 (0.1) | 6 (0.1) |  |
| Epidural anaesthesia | 4220 (21.4) | 3260 (36.3) | 0.33 |
| Vacuum | 1192 (6.1) | 655 (7.3) | 0.05 |
| Forceps | 47 (0.2) | 291 (3.2) | 0.23 |
| Infant birth weight (g) |  |  | 0.48 |
| <2500 | 7958 (40.4) | 4370 (48.7) |  |
| 2500-2999 | 7100 (36.0) | 3197 (35.6) |  |
| 3000-3499 | 3840 (19.5) | 1235 (13.8) |  |
| 3500-3999 | 675 (3.4) | 146 (1.6) |  |
| 4000-4499 | 43 (0.2) | 11 (0.1) |  |
| ≥4500 | <5 (0.0) | 0 (0.0) |  |
| Missing | 83 (0.4) | 13 (0.1) |  |
| Infant head circumference at birth (cm) |  |  | 0.18 |
| <33 | 5820 (29.5) | 3176 (35.4) |  |
| 33-34 | 8331 (42.3) | 3844 (42.8) |  |
| 35-36 | 3958 (20.1) | 1542 (17.2) |  |
| ≥37 | 317 (1.6) | 137 (1.5) |  |
| Missing | 1275 (6.5) | 273 (3.0) |  |
| Congenital anomaly | 976 (5.0) | 876 (9.8) | 0.18 |

*Standardized difference values > 0.1 are considered indicative of an imbalance between groups.
