# Supplementary material for: Crude and adjusted comparisons of cesarean delivery rates using the Robson classification: A population-based cohort study in Canada and Sweden, 2004 to 2016
Source: PLoS Med. 2022 Aug 1;19(8):e1004077. doi: 10.1371/journal.pmed.1004077 (PMC9377587; doi:10.1371/journal.pmed.1004077)
Supplement: S15 Table — Distribution of determinants of cesarean delivery in Robson Group 9. (DOCX) [file pmed.1004077.s017.docx]

S15 Table. Maternal, obstetric practice, and fetal/infant characteristics in deliveries among women in **Robson group 9**, Sweden and British Columbia, Canada, 2004-2016

| Maternal, obstetric practice or fetal/infant characteristic | Sweden (N=1905)  No. (%) | British Columbia (N=1828)  No. (%) | Standardized difference* |
| --- | --- | --- | --- |
| Maternal age (year) |  |  | 0.23 |
| <20 | 11 (0.6) | 12 (0.7) |  |
| 20-24 | 79 (4.1) | 131 (7.2) |  |
| 25-29 | 322 (16.9) | 375 (20.5) |  |
| 30-34 | 609 (32.0) | 560 (30.6) |  |
| 35-39 | 608 (31.9) | 522 (28.6) |  |
| 40-44 | 253 (13.3) | 208 (11.4) |  |
| ≥45 | 23 (1.2) | 20 (1.1) |  |
| Maternal body mass index (kg/m^2^) |  |  | 0.56 |
| Underweight (<18.5) | 21 (1.1) | 53 (2.9) |  |
| Normal weight (18.5-24.9) | 736 (38.6) | 609 (33.3) |  |
| Overweight (25.0-29.9) | 550 (28.9) | 348 (19.0) |  |
| Obese class I (30.0-34.9) | 251 (13.2) | 166 (9.1) |  |
| Obese class II (35.0-39.9) | 104 (5.5) | 69 (3.8) |  |
| Obese class III (≥40.0) | 49 (2.6) | 30 (1.6) |  |
| Missing | 194 (10.2) | 553 (30.3) |  |
| Parity |  |  | 0.44 |
| 0 | 487 (25.6) | 799 (43.7) |  |
| 1 | 771 (40.5) | 674 (36.9) |  |
| 2 | 375 (19.7) | 216 (11.8) |  |
| 3-4 | 219 (11.5) | 115 (6.3) |  |
| ≥5 | 53 (2.8) | 23 (1.3) |  |
| Smoking during pregnancy | 138 (7.2) | 163 (8.9) | 0.06 |
| Pre-existing diabetes | 12 (0.6) | 26 (1.4) | 0.08 |
| Preeclampsia/eclampsia | 62 (3.3) | 39 (2.1) | -0.07 |
| Chronic hypertension | 35 (1.8) | 24 (1.3) | -0.04 |
| In-vitro fertilization | 84 (4.4) | 49 (2.7) | -0.09 |
| Onset of labour |  |  | 0.42 |
| Spontaneous | 682 (35.8) | 636 (34.8) |  |
| Induced | 226 (11.9) | 266 (14.6) |  |
| Cesarean delivery before labour | 941 (49.4) | 926 (50.7) |  |
| Unknown | 56 (2.9) | 0 (0.0) |  |
| Gestational age (completed weeks) |  |  | 0.15 |
| Very early preterm (22-27) | 67 (3.5) | 38 (2.1) |  |
| Early preterm (28-31) | 90 (4.7) | 64 (3.5) |  |
| Late preterm (32-36) | 297 (15.6) | 258 (14.1) |  |
| Term (37-41) | 1396 (73.3) | 1440 (78.8) |  |
| Post-term (≥42) | 55 (2.9) | 26 (1.4) |  |
| Epidural anaesthesia | 130 (6.8) | 412 (22.5) | 0.46 |
| Infant birth weight (g) |  |  | 0.27 |
| <2500 | 314 (16.5) | 235 (12.9) |  |
| 2500-2999 | 305 (16.0) | 257 (14.1) |  |
| 3000-3499 | 501 (26.3) | 584 (31.9) |  |
| 3500-3999 | 476 (25.0) | 510 (27.9) |  |
| 4000-4499 | 211 (11.1) | 195 (10.7) |  |
| ≥4500 | 92 (4.8) | 43 (2.4) |  |
| Infant head circumference at birth (cm) |  |  | 0.20 |
| <33 | 257 (13.5) | 191 (10.4) |  |
| 33-34 | 432 (22.7) | 467 (25.5) |  |
| 35-36 | 765 (40.2) | 803 (43.9) |  |
| ≥37 | 365 (19.2) | 331 (18.1) |  |
| Missing | 86 (4.5) | 36 (2.0) |  |
| Congenital anomaly | 120 (6.3) | 142 (7.8) | 0.06 |

*Standardized difference values > 0.1 are considered indicative of an imbalance between groups.
