# Supplementary material for: Crude and adjusted comparisons of cesarean delivery rates using the Robson classification: A population-based cohort study in Canada and Sweden, 2004 to 2016
Source: PLoS Med. 2022 Aug 1;19(8):e1004077. doi: 10.1371/journal.pmed.1004077 (PMC9377587; doi:10.1371/journal.pmed.1004077)
Supplement: S16 Table — Distribution of determinants of cesarean delivery in Robson Group 10. (DOCX) [file pmed.1004077.s018.docx]

S16 Table. Maternal, obstetric practice, and fetal/infant characteristics in deliveries among women in **Robson group 10**, Sweden and British Columbia, Canada, 2004-2016

| Maternal, obstetric practice or fetal/infant characteristic | Sweden (N=58500)  No. (%) | British Columbia (N=37968)  No. (%) | Standardized difference* |
| --- | --- | --- | --- |
| Maternal age (year) |  |  |  |
| <20 | 1234 (2.1) | 877 (2.3) | 0.25 |
| 20-24 | 8367 (14.3) | 4260 (11.2) |  |
| 25-29 | 17203 (29.4) | 8915 (23.5) |  |
| 30-34 | 18506 (31.6) | 12350 (32.5) |  |
| 35-39 | 10352 (17.7) | 8744 (23.0) |  |
| 40-44 | 2674 (4.6) | 2606 (6.9) |  |
| ≥45 | 164 (0.3) | 216 (0.6) |  |
| Maternal body mass index (kg/m^2^) |  |  | 0.51 |
| Underweight (<18.5) | 1530 (2.6) | 1612 (4.2) |  |
| Normal weight (18.5-24.9) | 28411 (48.6) | 14851 (39.1) |  |
| Overweight (25.0-29.9) | 13587 (23.2) | 5789 (15.2) |  |
| Obese class I (30.0-34.9) | 5265 (9.0) | 2422 (6.4) |  |
| Obese class II (35.0-39.9) | 1967 (3.4) | 1059 (2.8) |  |
| Obese class III (≥40.0) | 757 (1.3) | 662 (1.7) |  |
| Missing | 6983 (11.9) | 11573 (30.5) |  |
| Parity |  |  | 0.15 |
| 0 | 31174 (53.3) | 17591 (46.3) |  |
| 1 | 16654 (28.5) | 12365 (32.6) |  |
| 2 | 6660 (11.4) | 5005 (13.2) |  |
| 3-4 | 3307 (5.7) | 2449 (6.5) |  |
| ≥5 | 705 (1.2) | 529 (1.4) |  |
| Missing | 0 (0.0) | 29 (0.1) |  |
| Smoking during pregnancy | 5429 (9.3) | 4655 (12.3) | 0.10 |
| Pre-existing diabetes | 1202 (2.1) | 730 (1.9) | -0.01 |
| Preeclampsia/eclampsia | 7048 (12.0) | 2406 (6.3) | -0.20 |
| Chronic hypertension | 1150 (2.0) | 753 (2.0) | 0.00 |
| In-vitro fertilization | 2198 (3.8) | 777 (2.0) | -0.10 |
| Onset of labour |  |  | 0.30 |
| Spontaneous | 35565 (60.8) | 21875 (57.6) |  |
| Induced | 10727 (18.3) | 10360 (27.3) |  |
| Cesarean delivery before labour | 11317 (19.3) | 5732 (15.1) |  |
| Unknown | 891 (1.5) | <5 (<0.0) |  |
| Gestational age (completed weeks) |  |  | 0.17 |
| Very early preterm (22-27) | 2478 (4.2) | 1087 (2.9) |  |
| Early preterm (28-31) | 4873 (8.3) | 2358 (6.2) |  |
| Late preterm (32-36) | 50739 (86.7) | 34523 (90.9) |  |
| Epidural anaesthesia | 12219 (20.9) | 10651 (28.1) | 0.17 |
| Vacuum | 2640 (4.5) | 1792 (4.7) | 0.01 |
| Forceps | 129 (0.2) | 1101 (2.9) | 0.22 |
| Infant birth weight (g) |  |  | 0.25 |
| <2500 | 25906 (44.3) | 13584 (35.8) |  |
| 2500-2999 | 19878 (34.0) | 13446 (35.4) |  |
| 3000-3499 | 9562 (16.3) | 8274 (21.8) |  |
| 3500-3999 | 2111 (3.6) | 2193 (5.8) |  |
| 4000-4499 | 492 (0.8) | 276 (0.7) |  |
| ≥4500 | 172 (0.3) | 73 (0.2) |  |
| Missing | 379 (0.6) | 122 (0.3) |  |
| Infant head circumference at birth (cm) |  |  | 0.30 |
| <33 | 25207 (43.1) | 13770 (36.3) |  |
| 33-34 | 20619 (35.2) | 15226 (40.1) |  |
| 35-36 | 6791 (11.6) | 6924 (18.2) |  |
| ≥37 | 673 (1.2) | 833 (2.2) |  |
| Missing | 5210 (8.9) | 1215 (3.2) |  |
| Fetal head in occiput posterior position at delivery | 2452 (4.2) | 1752 (4.6) | 0.02 |
| Congenital anomaly | 3977 (6.8) | 3510 (9.2) | 0.09 |

*Standardized difference values > 0.1 are considered indicative of an imbalance between groups.
