# Supplementary material for: Crude and adjusted comparisons of cesarean delivery rates using the Robson classification: A population-based cohort study in Canada and Sweden, 2004 to 2016
Source: PLoS Med. 2022 Aug 1;19(8):e1004077. doi: 10.1371/journal.pmed.1004077 (PMC9377587; doi:10.1371/journal.pmed.1004077)
Supplement: S17 Table — Estimates of temporal trends in determinants of cesarean delivery in Robson Group 1. (DOCX) [file pmed.1004077.s019.docx]

S17 Table. Frequency, proportion and rate ratio of maternal, obstetric practice, and fetal/infant characteristics among deliveries to women in **Robson group 1** in 2014-2016 vs 2004-2007, Sweden and British Columbia

| Maternal, obstetric practice or fetal/infant characteristic | Sweden | | | |  | British Columbia | | | |  |
| --- | --- | --- | --- | --- | --- | --- | --- | --- | --- | --- |
|  | 2004-2007 (N=128964)  No. (%) | 2014-2016 (N=97919)  No. (%) | Rate ratio (95% CI)  2014-16 vs 2004-07 | P value* |  | 2004-2007 (N=44906)  No. (%) | 2014-2016 (N=34495)  No. (%) | Rate ratio (95% CI)  2014-16 vs 2004-07 | P value* | |
| Advanced maternal age (≥35 years) | 12059 (9.4) | 9446 (9.7) | 1.03 (1.01-1.06) | <0.001 |  | 7451 (16.6) | 6475 (18.8) | 1.13 (1.10- 1.17) | <0.001 | |
|  |  |  |  |  |  |  |  |  |  | |
| Pre-pregnancy overweight/obesity (≥25 kg/m^2^) | 34200 (26.5) | 29555 (30.2) | 1.14 (1.12-1.15) | <0.001 |  | 7835 (17.5) | 7412 (21.5) | 1.23 (1.20-1.27) | <0.001 | |
|  |  |  |  |  |  |  |  |  |  | |
| Smoking during pregnancy | 9808 (7.6) | 4947 (5.1) | 0.66 (0.64-0.69) | <0.001 |  | 4673 (10.4) | 2074 (6.0) | 0.58 (0.55-0.61) | <0.001 | |
|  |  |  |  |  |  |  |  |  |  | |
| Pre-existing diabetes | 187 (0.2) | 147 (0.2) | 1.04 (0.83-1.28) | 0.63 |  | 57 (0.1) | 29 (0.1) | 0.66 (0.42-1.04) | 0.05 | |
|  |  |  |  |  |  |  |  |  |  | |
| Preeclampsia | 1475 (1.1) | 974 (1.0) | 0.87 (0.80-0.94) | 0.03 |  | 184 (0.4) | 201 (0.6) | 1.42 (1.16-1.74) | <0.001 | |
|  |  |  |  |  |  |  |  |  |  | |
| Chronic hypertension | 530 (0.4) | 324 (0.3) | 0.81 (0.70-0.92) | 0.005 |  | 89 (0.2) | 50 (0.1) | 0.73 (0.52-1.03) | 0.03 | |
|  |  |  |  |  |  |  |  |  |  | |
| Post-term delivery (≥42 weeks) | 7302 (5.7) | 4206 (4.3) | 0.76 (0.73-0.79) | <0.001 |  | 601 (1.3) | 399 (1.2) | 0.86 (0.76-0.98) | 0.008 | |
|  |  |  |  |  |  |  |  |  |  | |
| Epidural anesthesia | 13322 (45.3) | 51095 (52.2) | 1.15 (1.14-1.16) | <0.001 |  | 19962 (44.5) | 17741 (51.4) | 1.16 (1.14-1.17) | <0.001 | |
|  |  |  |  |  |  |  |  |  |  | |
| Vacuum | 19228 (14.9) | 11157 (11.4) | 0.76 (0.75-0.78) | <0.001 |  | 6136 (13.7) | 3679 (10.7) | 0.78 (0.75-0.81) | <0.001 | |
|  |  |  |  |  |  |  |  |  |  | |
| Forceps | 702 (0.5) | 157 (0.2) | 0.29 (0.25-0.35) | <0.001 |  | 2938 (6.5) | 2303 (6.7) | 1.02 (0.97-1.08) | 0.17 | |
|  |  |  |  |  |  |  |  |  |  | |
| Macrosomic infant (≥4000 g) | 11317 (14.4) | 13322 (13.6) | 0.94 (0.92-0.96) | <0.001 |  | 4514 (10.1) | 3006 (8.7) | 0.87 (0.83-0.91) | <0.001 | |
|  |  |  |  |  |  |  |  |  |  | |
| Head circumference ≥37 cm | 15792 (12.3) | 11317 (11.6) | 0.94 (0.92-0.97) | <0.001 |  | 3643 (8.1) | 3221 (9.3) | 1.15 (1.10-1.20) | <0.001 | |
|  |  |  |  |  |  |  |  |  |  | |
| Fetal head in occiput posterior position at delivery | 6139 (4.8) | 4886 (5.0) | 1.05 (1.01-1.09) | 0.01 |  | 3554 (7.9) | 2973 (8.6) | 1.09 (1.04-1.14) | 0.002 | |
|  |  |  |  |  |  |  |  |  |  | |
| Congenital anomaly | 4129 (3.2) | 3014 (3.1) | 0.96 (0.92-1.01) | 0.02 |  | 1842 (4.1) | 1585 (4.6) | 1.12 (1.05-1.20) | <0.001 | |

*P-value represents significance of 2-sided Cochran-Armitage test for linear trend in proportion by year (2004-2016); the a priori level of statistical significance was set at a 2-sided p value<0.05.
