# Supplementary material for: Crude and adjusted comparisons of cesarean delivery rates using the Robson classification: A population-based cohort study in Canada and Sweden, 2004 to 2016
Source: PLoS Med. 2022 Aug 1;19(8):e1004077. doi: 10.1371/journal.pmed.1004077 (PMC9377587; doi:10.1371/journal.pmed.1004077)
Supplement: S18 Table — Estimates of temporal trends in determinants of cesarean delivery in Robson Group 2a. (DOCX) [file pmed.1004077.s020.docx]

S18 Table. Frequency, proportion and rate ratio of maternal, obstetric practice, and fetal/infant characteristics among deliveries to women in **Robson group 2a** in 2014-2016 vs 2004-2007, Sweden and British Columbia

| Maternal, obstetric practice or fetal/infant characteristic | Sweden | | | | |  | | British Columbia | | | | |
| --- | --- | --- | --- | --- | --- | --- | --- | --- | --- | --- | --- | --- |
|  | 2004-2007 (N=24234)  No. (%) | 2014-2016 (N=27789)  No. (%) | Rate ratio (95% CI)  2014-16 vs 2004-07 | P value* |  | | 2004-2007 (N=17409)  No. (%) | | 2014-2016 (N=15403)  No. (%) | Rate ratio (95% CI)  2014-16 vs 2004-07 | P value* |  |
| Advanced maternal age (≥35 years) | 3897 (16.1) | 4622 (16.6) | 1.03 (0.99-1.08) | 0.08 |  | | 3374 (19.4) | | 3976 (25.8) | 1.33 (1.28-1.39) | <0.001 |  |
|  |  |  |  |  |  | |  | |  |  |  |  |
| Pre-pregnancy overweight/obesity (≥25 kg/m^2^) | 9064 (37.4) | 11841 (42.6) | 1.14 (1.12-1.16) | <0.001 |  | | 4819 (27.7) | | 5173 (33.6) | 1.21 (1.17-1.25) | <0.001 |  |
|  |  |  |  |  |  | |  | |  |  |  |  |
| Smoking during pregnancy | 1849 (7.6) | 1507 (5.4) | 0.71 (0.67-0.76) | <0.001 |  | | 1888 (10.8) | | 996 (6.5) | 0.60 (0.55-0.64) | <0.001 |  |
|  |  |  |  |  |  | |  | |  |  |  |  |
| Pre-existing diabetes | 294 (1.2) | 315 (1.1) | 0.93 (0.80-1.09) | 0.54 |  | | 114 (0.7) | | 176 (1.1) | 1.74 (1.38-2.21) | <0.001 |  |
|  |  |  |  |  |  | |  | |  |  |  |  |
| Preeclampsia | 3671 (15.1) | 3458 (12.4) | 0.82 (0.79-0.86) | <0.001 |  | | 586 (3.4) | | 872 (5.7) | 1.68 (1.52-1.86) | <0.001 |  |
|  |  |  |  |  |  | |  | |  |  |  |  |
| Chronic hypertension | 343 (1.4) | 297 (1.1) | 0.76 (0.65-0.88) | <0.001 |  | | 234 (1.3) | | 215 (1.4) | 1.04 (0.86-1.25) | 0.82 |  |
|  |  |  |  |  |  | |  | |  |  |  |  |
| Post-term delivery (≥42 weeks) | 7467 (30.8) | 9194 (33.1) | 1.07 (1.05-1.10) | <0.001 |  | | 638 (3.7) | | 340 (2.2) | 0.60 (0.53-0.69) | <0.001 |  |
|  |  |  |  |  |  | |  | |  |  |  |  |
| Epidural anesthesia | 14909 (61.5) | 19227 (69.2) | 1.12 (1.11-1.14) | <0.001 |  | | 10135 (58.2) | | 10476 (68.0) | 1.17 (1.15-1.19) | <0.001 |  |
|  |  |  |  |  |  | |  | |  |  |  |  |
| Vacuum | 4227 (17.4) | 3939 (14.2) | 0.81 (0.78-0.85) | <0.001 |  | | 2241 (12.9) | | 1415 (9.2) | 0.71 (0.67-0.76) | <0.001 |  |
|  |  |  |  |  |  | |  | |  |  |  |  |
| Forceps | 141 (0.6) | 41 (0.1) | 0.25 (0.18-0.36) | <0.001 |  | | 1118 (6.4) | | 1039 (6.7) | 1.05 (0.97-1.14) | 0.08 |  |
|  |  |  |  |  |  | |  | |  |  |  |  |
| Macrosomic infant (≥4000 g) | 5418 (22.4) | 6211 (22.4) | 0.99 (0.97-1.03) | 0.71 |  | | 2819 (16.2) | | 2047 (13.3) | 0.82 (0.78-0.87) | <0.001 |  |
|  |  |  |  |  |  | |  | |  |  |  |  |
| Head circumference ≥37 cm | 5036 (20.8) | 5964 (21.5) | 1.03 (0.99-1.07) | 0.02 |  | | 2537 (14.6) | | 2379 (15.5) | 1.06 (1.01-1.12) | 0.01 |  |
|  |  |  |  |  |  | |  | |  |  |  |  |
| Fetal head in occiput posterior position at delivery | 1508 (6.2) | 1681 (6.0) | 0.97 (0.91-1.04) | 0.30 |  | | 1647 (9.5) | | 1598 (10.4) | 1.10 (1.03-1.17) | 0.05 |  |
|  |  |  |  |  |  | |  | |  |  |  |  |
| Congenital anomaly | 976 (4.0) | 1033 (3.7) | 0.92 (0.85-1.01) | 0.07 |  | | 858 (4.9) | | 891 (5.8) | 1.17 (1.07-1.29) | <0.001 |  |

*P-value represents significance of 2-sided Cochran-Armitage test for linear trend in proportion by year (2004-2016); the a priori level of statistical significance was set at a 2-sided p value<0.05.
