# Supplementary material for: Crude and adjusted comparisons of cesarean delivery rates using the Robson classification: A population-based cohort study in Canada and Sweden, 2004 to 2016
Source: PLoS Med. 2022 Aug 1;19(8):e1004077. doi: 10.1371/journal.pmed.1004077 (PMC9377587; doi:10.1371/journal.pmed.1004077)
Supplement: S19 Table — Estimates of temporal trends in determinants of cesarean delivery in Robson Group 5. (DOCX) [file pmed.1004077.s021.docx]

S19 Table. Frequency, proportion and rate ratio of maternal, obstetric practice, and fetal/infant characteristics among deliveries to women in **Robson group 5** in 2014-2016 vs 2004-2007, Sweden and British Columbia

| Maternal, obstetric practice or fetal/infant characteristic | Sweden | | | | |  | | British Columbia | | | | |
| --- | --- | --- | --- | --- | --- | --- | --- | --- | --- | --- | --- | --- |
|  | 2004-2007 (N=32534)  No. (%) | 2014-2016 (N=31035)  No. (%) | Rate ratio (95% CI)  2014-16 vs 2004-07 | P value* |  | | 2004-2007 (N=20003)  No. (%) | | 2014-2016 (N=17870)  No. (%) | Rate ratio (95% CI)  2014-16 vs 2004-07 | P value* |  |
| Advanced maternal age (≥35 years) | 11200 (34.4) | 11378 (36.7) | 1.07 (1.04-1.09) | <0.001 |  | | 7953 (39.8) | | 7975 (44.6) | 1.12 (1.10-1.15) | <0.001 |  |
|  |  |  |  |  |  | |  | |  |  |  |  |
| Pre-pregnancy overweight/obesity (≥25 kg/m^2^) | 13734 (42.2) | 15078 (48.6) | 1.15 (1.13-1.17) | <0.001 |  | | 5549 (27.7) | | 5764 (32.3) | 1.16 (1.13-1.20) | <0.001 |  |
|  |  |  |  |  |  | |  | |  |  |  |  |
| Smoking during pregnancy | 2635 (8.1) | 1705 (5.5) | 0.68 (0.64-0.72) | <0.001 |  | | 1716 (8.6) | | 944 (5.3) | 0.62 (0.57-0.66) | <0.001 |  |
|  |  |  |  |  |  | |  | |  |  |  |  |
| Pre-existing diabetes | 337 (1.0) | 314 (1.0) | 0.98 (0.84-1.14) | 0.62 |  | | 126 (0.6) | | 138 (0.8) | 1.23 (0.96-1.56) | 0.01 |  |
|  |  |  |  |  |  | |  | |  |  |  |  |
| Preeclampsia | 670 (2.1) | 570 (1.8) | 0.89 (0.80-0.99) | 0.009 |  | | 98 (0.5) | | 156 (0.9) | 1.78 (1.39-2.29) | <0.001 |  |
|  |  |  |  |  |  | |  | |  |  |  |  |
| Chronic hypertension | 355 (1.1) | 345 (1.1) | 1.02 (0.88-1.18) | 0.83 |  | | 144 (0.7) | | 141 (0.8) | 1.10 (0.87-1.38) | 0.34 |  |
|  |  |  |  |  |  | |  | |  |  |  |  |
| Post-term delivery (≥42 weeks) | 1913 (5.9) | 1811 (5.8) | 0.99 (0.93-1.06) | 0.70 |  | | 137 (0.7) | | 96 (0.5) | 0.78 (0.60-1.02) | 0.03 |  |
|  |  |  |  |  |  | |  | |  |  |  |  |
| Epidural anesthesia | 8161 (25.1) | 8878 (28.6) | 1.14 (1.11-1.17) | <0.001 |  | | 2098 (10.5) | | 2446 (13.7) | 1.31 (1.24-1.38) | <0.001 |  |
|  |  |  |  |  |  | |  | |  |  |  |  |
| Vacuum | 2325 (7.2) | 1571 (5.1) | 0.71 (0.67-0.75) | <0.001 |  | | 461 (2.3) | | 476 (2.7) | 1.16 (1.02-1.31) | 0.02 |  |
|  |  |  |  |  |  | |  | |  |  |  |  |
| Forceps | 74 (0.2) | 23 (0.1) | 0.33 (0.20-0.52) | <0.001 |  | | 188 (0.9) | | 237 (1.3) | 1.41 (1.17-1.71) | <0.001 |  |
|  |  |  |  |  |  | |  | |  |  |  |  |
| Macrosomic infant (≥4000 g) | 7482 (23.0) | 6704 (21.6) | 0.94 (0.91-0.97) | <0.001 |  | | 2705 (13.5) | | 2298 (12.9) | 0.95 (0.90-1.00) | 0.03 |  |
|  |  |  |  |  |  | |  | |  |  |  |  |
| Head circumference ≥37 cm | 6937 (21.3) | 6349 (20.5) | 0.96 (0.93-0.99) | 0.03 |  | | 3435 (17.2) | | 3131 (17.5) | 1.02 (0.98-1.07) | 0.20 |  |
|  |  |  |  |  |  | |  | |  |  |  |  |
| Fetal head in occiput posterior position at delivery | 1319 (4.1) | 1368 (4.4) | 1.09 (1.01-1.17) | 0.01 |  | | 676 (3.4) | | 671 (3.8) | 1.11 (1.00-1.23) | 0.05 |  |
|  |  |  |  |  |  | |  | |  |  |  |  |
| Congenital anomaly | 1095 (3.4) | 1020 (3.3) | 0.98 (0.90-1.06) | 0.13 |  | | 829 (4.1) | | 904 (5.1) | 1.22 (1.11-1.34) | <0.001 |  |

*P-value represents significance of 2-sided Cochran-Armitage test for linear trend in proportion by year (2004-2016); the a priori level of statistical significance was set at a 2-sided p value<0.05.
