# Supplementary material for: Crude and adjusted comparisons of cesarean delivery rates using the Robson classification: A population-based cohort study in Canada and Sweden, 2004 to 2016
Source: PLoS Med. 2022 Aug 1;19(8):e1004077. doi: 10.1371/journal.pmed.1004077 (PMC9377587; doi:10.1371/journal.pmed.1004077)
Supplement: S20 Table — Comparing the crude and adjusted differences in cesarean delivery rates by country and study period in Robson Groups 1, 2a, and 5. (DOCX) [file pmed.1004077.s022.docx]

S20 Table. Crude and adjusted rate ratios for cesarean delivery in 2008-2010, 2011-2013 and 2014-2016 vs 2004-2007 among women in Robson Groups 1, 2a, and 5 after sequential adjustment* for maternal characteristics, obstetric practice factors and fetal/infant characteristics, Sweden and British Columbia, Canada

| Period (years) | Robson Group 1 | | | |  | Robson Group 2a | | | |  | | Robson Group 5 | | | | | | |  |
| --- | --- | --- | --- | --- | --- | --- | --- | --- | --- | --- | --- | --- | --- | --- | --- | --- | --- | --- | --- |
|  | Sweden | | British Columbia | |  | Sweden | | British Columbia | |  | | Sweden | | | | British Columbia | | | |
|  | RR (95% CI) | P-value^†^ | RR (95% CI) | P-value^†^ |  | RR (95% CI) | P-value^†^ | RR (95% CI) | P-value^†^ | |  | | RR (95% CI) | P-value^†^ | | RR (95% CI) | P-  value^†^ | | |
| Unadjusted |  |  |  |  |  |  |  |  |  | |  | |  |  | |  |  | | |
| 2008-2010 | 1.00 (0.97-1.03) | 0.94 | 1.00 (0.97-1.03) | 0.89 |  | 0.97 (0.94-0.99) | 0.04 | 1.02 (0.99-1.05) | 0.18 | |  | | 1.00 (0.99-1.02) | 0.82 | | 0.98 (0.97-0.99) | 0.04 | | |
| 2011-2013 | 0.96 (0.94-0.99) | 0.04 | 1.07 (1.04-1.10) | <0.001 |  | 0.91 (0.88-0.94) | <0.001 | 1.11 (1.08-1.15) | <0.001 | |  | | 1.02 (1.00-1.03) | 0.04 | | 0.97 (0.97-0.99) | 0.02 | | |
| 2014-2016 | 0.93 (0.91-0.96) | <0.001 | 1.12 (1.09-1.16) | <0.001 |  | 0.84 (0.82-0.87) | <0.001 | 1.18 (1.15-1.21) | <0.001 | |  | | 1.05 (1.03-1.06) | <0.001 | | 0.98 (0.97-0.99) | 0.01 | | |
| Adjusted for maternal characteristics^a^ | | | | |  |  |  |  |  | |  | |  | |  |  | |  | |
| 2008-2010 | 1.01 (0.98-1.03) | 0.94 | 0.99 (0.97-1.02) | 0.63 |  | 0.97 (0.94-0.99) | 0.04 | 1.00 (0.98-1.03) | 0.80 | |  | | 0.99 (0.98-1.01) | 0.30 | | 0.98 (0.97-0.99) | 0.04 | | |
| 2011-2013 | 0.96 (0.93-0.99) | 0.04 | 1.03 (1.00-1.06) | 0.05 |  | 0.91 (0.89-0.94) | <0.001 | 1.07 (1.04-1.10) | <0.001 | |  | | 1.01 (0.99-1.03) | 0.37 | | 0.98 (0.97-0.99) | 0.03 | | |
| 2014-2016 | 0.92 (0.90-0.95) | <0.001 | 1.06 (1.03-1.09) | <0.001 |  | 0.84 (0.82-0.87) | <0.001 | 1.11 (1.08-1.14) | <0.001 | |  | | 1.05 (1.03-1.06) | <0.001 | | 0.98 (0.97-0.99) | 0.03 | | |
| Also adjusted for maternal conditions^b^ | | | | |  |  |  |  |  | |  | |  | |  |  | |  | |
| 2008-2010 | 1.01 (0.98-1.04) | 0.94 | 0.99 (0.96-1.02) | 0.58 |  | 0.97 (0.94-0.99) | 0.04 | 1.00 (0.97-1.03) | 0.90 | |  | | 0.99 (0.98-1.01) | 0.87 | | 0.98 (0.97-0.99) | 0.04 | | |
| 2011-2013 | 0.96 (0.93-0.99) | 0.04 | 1.03 (0.99-1.06) | 0.09 |  | 0.91 (0.89-0.94) | <0.001 | 1.07 (1.04-1.10) | <0.001 | |  | | 1.01 (0.99-1.02) | 0.06 | | 0.97 (0.97-0.98) | 0.02 | | |
| 2014-2016 | 0.93 (0.90-0.95) | <0.001 | 1.06 (1.03-1.09) | <0.001 |  | 0.85 (0.82-0.87) | <0.001 | 1.11 (1.08-1.14) | <0.001 | |  | | 1.04 (1.03-1.06) | <0.001 | | 0.98 (0.97-0.99) | 0.04 | | |
| Also adjusted for obstetric practice factors^c^ | | | | |  |  |  |  |  | |  | |  | |  |  | |  | |
| 2008-2010 | 1.00 (0.97-1.02) | 0.95 | 0.98 (0.96-1.01) | 0.21 |  | 0.97 (0.95-0.99) | 0.04 | 0.99 (0.97-1.03) | 0.99 | |  | | 0.99 (0.98-1.01) | 0.87 | | 0.98 (0.97-0.99) | 0.04 | | |
| 2011-2013 | 0.94 (0.91-0.96) | <0.001 | 1.00 (0.98-1.03) | 0.79 |  | 0.92 (0.89-0.94) | <0.001 | 1.06 (1.03-1.09) | 0.003 | |  | | 1.01 (0.99-1.02) | 0.06 | | 0.97 (0.97-0.98) | 0.02 | | |
| 2014-2016 | 0.89 (0.86-0.91) | <0.001 | 1.01 (0.98-1.03) | 0.74 |  | 0.85 (0.82-0.87) | <0.001 | 1.09 (1.06-1.12) | <0.001 | |  | | 1.04 (1.03-1.06) | <0.001 | | 0.98 (0.97-0.99) | 0.04 | | |
| Also adjusted for fetal/infant characteristics^d^ | | | | |  |  |  |  |  | |  | |  | |  |  | |  | |
| 2008-2010 | 1.00 (0.98-1.03) | 0.95 | 0.95 (0.93-0.97) | 0.001 |  | 0.98 (0.95-1.01) | 0.05 | 0.98 (0.95-1.00) | 0.87 | |  | | 0.99 (0.98-1.01) | 0.12 | | 0.98 (0.97-0.99) | 0.04 | | |
| 2011-2013 | 0.95 (0.92-0.97) | <0.001 | 0.99 (0.97-1.01) | 0.82 |  | 0.93 (0.91-0.96) | <0.001 | 1.06 (1.03-1.09) | 0.001 | |  | | 1.01 (0.99-1.02) | 0.52 | | 0.97 (0.96-0.98) | 0.02 | | |
| 2014-2016 | 0.91 (0.88-0.93) | <0.001 | 1.01 (0.99-1.04) | 0.80 |  | 0.86 (0.84-0.88) | <0.001 | 1.09 (1.07-1.13) | <0.001 | |  | | 1.04 (1.03-1.06) | <0.001 | | 0.97 (0.96-0.98) | 0.03 | | |

The period of 2004-2007 is the reference group. RR, rate ratio; CI, confidence interval.

*Sequential adjustment was carried out by fitting a series of models with additional groups of factors added to each model in the sequence outlined above to quantify the contribution of each group of factors to CD trends over time.

†P-values represent significance of Wald chi-square test; the a priori level of statistical significance was set at a 2-sided p value<0.05.

^a^Maternal characteristics included maternal age, pre-pregnancy body mass index, smoking during pregnancy and parity (only for group 5).

^b^Maternal conditions included preeclampsia/eclampsia, pre-existing diabetes, in-vitro fertilization, and chronic hypertension.

^c^Obstetric practice factors included post-term delivery, and epidural anaesthesia (in groups 1 and 2a only).

^d^Fetal/infant characteristics included position of the fetal head at delivery, infant birth weight, infant head circumference, and congenital anomaly.
