# Supplementary material for: Crude and adjusted comparisons of cesarean delivery rates using the Robson classification: A population-based cohort study in Canada and Sweden, 2004 to 2016
Source: PLoS Med. 2022 Aug 1;19(8):e1004077. doi: 10.1371/journal.pmed.1004077 (PMC9377587; doi:10.1371/journal.pmed.1004077)
Supplement: S21 Table — Distribution of deliveries and cesarean deliveries restricted to women in British Columbia with midwifery-led maternity care. (DOCX) [file pmed.1004077.s023.docx]

S21 Table. Rate of cesarean delivery by Robson classification groups **among women with midwifery care**, British Columbia, Canada, 2004-2016

| Robson  Group | Deliveries  No. | Cesarean deliveries  No. | Cesarean delivery rate (%) | Relative size (%) | Contribution to overall caesarean delivery rate (%) |
| --- | --- | --- | --- | --- | --- |
| 1 | 29070 | 4993 | 17.18 | 35.17 | 6.04 |
| 2 | 7204 | 3213 | 44.60 | 8.71 | 3.89 |
| 2a | 6765 | 2774 | 41.01 | 8.18 | 3.35 |
| 2b | 439 | 439 | 100.00 | 0.53 | 0.53 |
| 3 | 28375 | 408 | 1.44 | 34.32 | 0.49 |
| 4 | 3481 | 405 | 11.63 | 4.21 | 0.49 |
| 4a | 3286 | 210 | 6.39 | 3.97 | 0.25 |
| 4b | 195 | 195 | 100.00 | 0.24 | 024 |
| 5 | 6562 | 3284 | 50.05 | 7.94 | 3.97 |
| 6 | 1903 | 1771 | 93.06 | 2.30 | 2.14 |
| 7 | 796 | 652 | 81.91 | 0.96 | 0.79 |
| 8 | 601 | 303 | 50.42 | 0.73 | 0.37 |
| 9 | 169 | 145 | 85.80 | 0.20 | 0.18 |
| 10 | 3883 | 736 | 18.95 | 4.70 | 0.89 |
| Unknown* | 622 | 501 | 80.55 | 0.75 | 0.61 |
| All groups | 82666 | 16411 | 19.85 | 100.00 | 19.85 |

No., number.

*All remaining records that could not be classified due to missing information on one or more of the following variables: fetal presentation, parity, gestational age, type of labour, or previous cesarean delivery.
