# Supplementary material for: Crude and adjusted comparisons of cesarean delivery rates using the Robson classification: A population-based cohort study in Canada and Sweden, 2004 to 2016
Source: PLoS Med. 2022 Aug 1;19(8):e1004077. doi: 10.1371/journal.pmed.1004077 (PMC9377587; doi:10.1371/journal.pmed.1004077)
Supplement: S22 Table — Comparing Robson group-specific cesarean delivery rates by country restricted to women in British Columbia with midwifery-led maternity care. (DOCX) [file pmed.1004077.s024.docx]

S22 Table. Crude and adjusted rates and rate ratios for cesarean delivery **among women with midwifery care**, British Columbia, Canada, vs Sweden, by Robson group, 2004-2016

| Robson  Group |  | Crude cesarean delivery rate | |  | Cesarean delivery  British Columbia (midwifery care) vs Sweden | | | | | Absolute change in cesarean delivery rate in British Columbia after adjustment^‡^ (%) |
| --- | --- | --- | --- | --- | --- | --- | --- | --- | --- | --- |
|  |  | Sweden | British Columbia (midwifery care) |  | RR (95% CI) | P-value* |  | ARR^†^ (95% CI) | P-value* |  |
| 1 |  | 8.1 | 17.2 |  | 2.12 (2.07-2.18) | <0.001 |  | 1.84 (1.80-1.89)^a,b^ | <0.001 | -2.3 |
| 2 |  | 37.3 | 44.6 |  | 1.20 (1.16-1.23) | <0.001 |  | 1.01 (0.98-1.04)^b^ | 0.12 | -6.9 |
| 3 |  | 1.6 | 1.4 |  | 0.89 (0.80-0.98) | <0.001 |  | 0.95 (0.86-1.04)^a^ | 0.09 | +0.1 |
| 4 |  | 21.5 | 11.6 |  | 0.54 (0.49-0.59) | <0.001 |  | 0.44 (0.40-0.48) | <0.001 | -2.1 |
| 5 |  | 51.6 | 50.0 |  | 0.97 (0.95-0.99) | 0.03 |  | 0.94 (0.93-0.97) | 0.001 | -1.5 |
| 6 |  | 93.8 | 93.1 |  | 0.99 (0.98-1.00) | 0.09 |  | 0.98 (0.96-0.99)^b,c^ | 0.04 | -1.2 |
| 7 |  | 88.5 | 81.9 |  | 0.93 (0.90-0.96) | 0.001 |  | 0.91 (0.89-0.94)^c^ | 0.01 | -1.4 |
| 8 |  | 54.7 | 50.4 |  | 0.92 (0.85-0.99) | 0.01 |  | 0.92 (0.85-0.99)^c^ | 0.04 | -0.1 |
| 9 |  | 99.3 | 85.8 |  | 0.86 (0.81-0.92) | <0.001 |  | 0.87 (0.82-0.92)^c^ | 0.001 | +0.6 |
| 10 |  | 29.4 | 19.0 |  | 0.65 (0.60-0.69) | <0.001 |  | 0.78 (0.73-0.83)^d^ | <0.001 | +3.9 |
| All groups |  | 17.3 | 19.9 |  | 1.15 (1.13-1.16) | <0.001 |  |  |  |  |

RR, rate ratio; CI, confidence interval; ARR, adjusted rate ratio.

*P-values represent significance of Wald chi-square test; the a priori level of statistical significance was set at a 2-sided p value<0.05.

†Adjusted models included maternal age, parity, pre-pregnancy body mass index, smoking during pregnancy, preeclampsia/eclampsia, pre-existing diabetes, in-vitro fertilization, chronic hypertension, post-term delivery, position of the fetal head at delivery, infant birth weight, infant head circumference, congenital anomaly.

‡Absolute change in cesarean delivery rate in British Columbia after adjustment=(crude cesarean delivery rate in Sweden*ARR)‒crude cesarean delivery rate in British Columbia.

^a^Adjusted model also included epidural anaesthesia.

^b^Adjusted model excluded parity.

^c^Adjusted model excluded position of the fetal head at delivery.

^d^Adjusted model excluded post-term delivery.
