# Supplementary material for: Crude and adjusted comparisons of cesarean delivery rates using the Robson classification: A population-based cohort study in Canada and Sweden, 2004 to 2016
Source: PLoS Med. 2022 Aug 1;19(8):e1004077. doi: 10.1371/journal.pmed.1004077 (PMC9377587; doi:10.1371/journal.pmed.1004077)
Supplement: S23 Table — Comparing cesarean delivery rates in British Columbia vs. Sweden. (A) Excluding deliveries with missing values for body mass index and (B) using multiple imputation for missing body mass index values. (DOCX) [file pmed.1004077.s025.docx]

S23 Table. Crude and adjusted rates and rate ratios (RRs) for cesarean delivery, British Columbia, Canada vs Sweden, by Robson group, 2004-2016

| 1. **Excluding deliveries with missing values for body mass index** | | | | | | | | | | |
| --- | --- | --- | --- | --- | --- | --- | --- | --- | --- | --- |
| Robson  Group |  | Crude cesarean delivery rate | |  | Cesarean delivery  British Columbia vs Sweden | | | | | |
|  |  | Sweden | British Columbia |  | RR (95% CI) | P-value* | |  | ARR^†^ (95% CI) | P-value* |
| 1 |  | 8.0 | 21.0 |  | 2.63 (2.59-2.67) | <0.001 | |  | 2.33 (2.30-2.37)^a,b^ | <0.001 |
| 2 |  | 36.7 | 45.6 |  | 1.24 (1.23-1.26) | <0.001 | |  | 1.11 (1.10-1.13)^b^ | <0.001 |
| 3 |  | 1.6 | 2.6 |  | 1.67 (1.59-1.74) | <0.001 | |  | 1.56 (1.49-1.63)^a^ | <0.001 |
| 4 |  | 21.1 | 12.7 |  | 0.60 (0.58-0.62) | <0.001 | |  | 0.49 (0.47-0.50) | <0.001 |
| 5 |  | 51.4 | 78.5 |  | 1.53 (1.51-1.54) | <0.001 | |  | 1.46 (1.45-1.47) | <0.001 |
| 6 |  | 94.6 | 96.7 |  | 1.02 (1.02-1.03) | <0.001 | |  | 1.02 (1.02-1.03)^b,c^ | 0.03 |
| 7 |  | 89.4 | 91.9 |  | 1.03 (1.02-1.04) | <0.001 | |  | 1.03 (1.02-1.04)^c^ | 0.03 |
| 8 |  | 53.5 | 68.4 |  | 1.28 (1.25-1.31) | <0.001 | |  | 1.21 (1.18-1.24)^c^ | <0.001 |
| 9 |  | 99.3 | 94.2 |  | 0.95 (0.94-0.96) | <0.001 | |  | 0.95 (0.94-0.97)^c^ | 0.03 |
| 10 |  | 28.0 | 31.0 |  | 1.11 (1.08-1.13) | <0.001 | |  | 1.15 (1.13-1.18)^d^ | <0.001 |
| 1. **Using multiple imputation for missing body mass index values** | | | | | | | | | | |
| Robson  group |  | Crude cesarean delivery rate | |  | Cesarean delivery  British Columbia vs Sweden | | | | | |
|  |  | Sweden | British Columbia |  | RR (95% CI) | P-value* |  | | ARR^†^ (95% CI) | P-value* |
| 1 |  | 8.1 | 20.4 |  | 2.52 (2.49-2.56) | <0.001 |  | | 2.34 (2.30-2.37)^a,b^ | <0.001 |
| 2 |  | 37.3 | 45.9 |  | 1.23 (1.22-1.25) | <0.001 |  | | 1.12 (1.10-1.13)^b^ | <0.001 |
| 3 |  | 1.6 | 2.6 |  | 1.60 (1.54-1.66) | <0.001 |  | | 1.55 (1.49-1.62)^a^ | <0.001 |
| 4 |  | 21.5 | 13.1 |  | 0.61 (0.59-0.62) | <0.001 |  | | 0.52 (0.51-0.54) | <0.001 |
| 5 |  | 51.6 | 79.7 |  | 1.54 (1.54-1.56) | <0.001 |  | | 1.48 (1.47-1.49) | <0.001 |
| 6 |  | 93.8 | 95.5 |  | 1.02 (1.01-1.02) | <0.001 |  | | 1.01 (1.01-1.02)^b,c^ | 0.03 |
| 7 |  | 88.5 | 90.2 |  | 1.02 (1.01-1.03) | <0.001 |  | | 1.01 (1.00-1.02)^c^ | 0.05 |
| 8 |  | 54.7 | 69.6 |  | 1.27 (1.25-1.30) | <0.001 |  | | 1.21 (1.19-1.23)^c^ | <0.001 |
| 9 |  | 99.3 | 94.7 |  | 0.95 (0.94-0.96) | <0.001 |  | | 0.96 (0.95-0.97)^c^ | 0.03 |
| 10 |  | 29.4 | 30.8 |  | 1.04 (1.03-1.07) | <0.001 |  | | 1.11 (1.09-1.13)^d^ | <0.001 |

CI, confidence interval; ARR, adjusted rate ratio.

*P-values represent significance of Wald chi-square test; the a priori level of statistical significance was set at a 2-sided p value<0.05.

†Adjusted models included maternal age, parity, pre-pregnancy body mass index, smoking during pregnancy, preeclampsia/eclampsia, pre-existing diabetes, in-vitro fertilization, chronic hypertension, post-term delivery, position of the fetal head at delivery, infant birth weight, infant head circumference, congenital anomaly.

^a^Adjusted model also included epidural anaesthesia.

^b^Adjusted model excluded parity.

^c^Adjusted model excluded position of the fetal head at delivery.

^d^Adjusted model excluded post-term delivery.
