# Supplementary material for: Crude and adjusted comparisons of cesarean delivery rates using the Robson classification: A population-based cohort study in Canada and Sweden, 2004 to 2016
Source: PLoS Med. 2022 Aug 1;19(8):e1004077. doi: 10.1371/journal.pmed.1004077 (PMC9377587; doi:10.1371/journal.pmed.1004077)
Supplement: S24 Table — Comparing crude and adjusted temporal changes in the cesarean delivery rate by country. (A) Excluding deliveries with missing values for body mass index, and (B) using multiple imputation for missing body mass index values. (DOCX) [file pmed.1004077.s026.docx]

S24 Table. Crude and sequentially adjusted* rate ratios of cesarean delivery rates in 2014-16 vs 2004-2007 in Robson groups 1, 2a, and 5, Sweden and British Columbia, Canada

| 1. **Excluding deliveries with missing values for body mass index** | | | | | | | | | | | | | | | |
| --- | --- | --- | --- | --- | --- | --- | --- | --- | --- | --- | --- | --- | --- | --- | --- |
| Model adjustment |  | Robson group 1 | | | |  | Robson group 2a | | | |  | Robson group 5 | | | |
|  |  | Sweden | | British Columbia | |  | Sweden | | British Columbia | |  | Sweden | | British Columbia | |
|  |  | RR (95% CI) | P-value^†^ | RR (95% CI) | P-value^†^ |  | RR (95% CI) | P-  value^†^ | RR (95% CI) | P-value^†^ |  | RR (95% CI) | P-value^†^ | RR (95% CI) | P-  value^†^ |
| Crude |  | 0.93  (0.90-0.96) | <0.001 | 1.11  (1.07-1.14) | <0.001 |  | 0.84  (0.81-0.86) | <0.001 | 1.16  (1.12-1.19) | <0.001 |  | 1.05  (1.04-1.07) | <0.001 | 0.99  (0.98-1.01) | 0.89 |
| Maternal characteristics |  | 0.92  (0.89-0.95) | <0.001 | 1.06  (1.03-1.09) | <0.001 |  | 0.83  (0.81-0.86) | <0.001 | 1.07  (1.06-1.13) | <0.001 |  | 1.04  (1.03-1.06) | <0.001 | 0.99  (0.98-1.01) | 0.91 |
| + Maternal conditions |  | 0.92  (0.89-0.95) | <0.001 | 1.06  (1.03-1.09) | <0.001 |  | 0.83  (0.81-0.86) | <0.001 | 1.09  (1.06-1.12) | <0.001 |  | 1.04  (1.03-1.06) | <0.001 | 0.99  (0.98-1.01) | 0.89 |
| + Practice factors |  | 0.88  (0.85-0.91) | <0.001 | 1.02  (0.99-1.05) | 0.77 |  | 0.83  (0.81-0.86) | <0.001 | 1.08  (1.05-1.11) | <0.001 |  | 1.04  (1.03-1.06) | <0.001 | 0.99  (0.98-1.00) | 0.09 |
| + Fetal characteristics |  | 0.89  (0.87-0.92) | <0.001 | 1.02  (0.99-1.04) | 0.86 |  | 0.85  (0.82-0.87) | <0.001 | 1.09  (1.06-1.12) | <0.001 |  | 1.04  (1.03-1.06) | <0.001 | 0.99  (0.97-0.99) | 0.04 |
| 1. **Using multiple imputation for missing body mass index values** | | | | | | | | | | | | | | | |
| Model adjustment |  | Robson group 1 | | | |  | Robson group 2a | | | |  | Robson group 5 | | | |
|  |  | Sweden | | British Columbia | |  | Sweden | | British Columbia | |  | Sweden | | British Columbia | |
|  |  | RR (95% CI) | P-value | RR (95% CI) | P-value |  | RR (95% CI) | P-value | RR (95% CI) | P-value |  | RR (95% CI) | P-value | RR (95% CI) | P-value |
| Crude |  | 0.93  (0.91-0.96) | <0.001 | 1.12  (1.09-1.16) | <0.001 |  | 0.84  (0.82-0.87) | <0.001 | 1.18  (1.15-1.21) | <0.001 |  | 1.05  (1.03-1.06) | <0.001 | 1.01  (0.99-1.02) | 0.89 |
| Maternal characteristics |  | 0.92  (0.89-0.94) | <0.001 | 1.07  (1.04-1.10) | <0.001 |  | 0.84  (0.82-0.86) | <0.001 | 1.12  (1.09-1.15) | <0.001 |  | 1.04  (1.03-1.06) | <0.001 | 1.00  (0.99-1.01) | 0.92 |
| + Maternal conditions |  | 0.92  (0.89-0.94) | <0.001 | 1.07  (1.04-1.10) | <0.001 |  | 0.84  (0.82-0.86) | <0.001 | 1.11  (1.08-1.14) | <0.001 |  | 1.04  (1.03-1.06) | <0.001 | 1.00  (0.99-1.01) | 0.97 |
| + Practice factors |  | 0.89  (0.85-0.91) | <0.001 | 1.01  (0.98-1.03) | 0.92 |  | 0.84  (0.82-0.86) | <0.001 | 1.09  (1.06-1.12) | <0.001 |  | 1.04  (1.03-1.06) | <0.001 | 1.00  (0.99-1.01) | 0.99 |
| + Fetal characteristics |  | 0.90  (0.87-0.92) | <0.001 | 1.01  (0.99-1.04) | 0.89 |  | 0.85  (0.83-0.88) | <0.001 | 1.10  (1.08-1.13) | <0.001 |  | 1.04  (1.03-1.06) | <0.001 | 0.99  (0.98-1.00) | 0.98 |

RR, rate ratio; CI, confidence interval.

*Sequential adjustment was carried out by fitting a series of models with additional groups of factors added to each model in the sequence outlined above to quantify the contribution of each group of factors to CD trends over time.

†P-values represent significance of Wald chi-square test; the a priori level of statistical significance was set at a 2-sided p value<0.05.

^a^Maternal characteristics included maternal age, pre-pregnancy body mass index, smoking during pregnancy and parity (only for group 5).

^b^Maternal conditions included preeclampsia/eclampsia, pre-existing diabetes, in-vitro fertilization, and chronic hypertension.

^c^Obstetric practice factors included post-term delivery, and epidural anaesthesia (in groups 1 and 2a only).

^d^Fetal/infant characteristics included position of the fetal head at delivery, infant birth weight, infant head circumference, and congenital anomaly.
