# Supplementary figures and images for: Crude and adjusted comparisons of cesarean delivery rates using the Robson classification: A population-based cohort study in Canada and Sweden, 2004 to 2016
Source: PLoS Med. 2022 Aug 1;19(8):e1004077. doi: 10.1371/journal.pmed.1004077 (PMC9377587; doi:10.1371/journal.pmed.1004077)

S1 Fig. Rate of preeclampsia/eclampsia by Robson Group, Sweden and British Columbia, Canada, 2004-2016

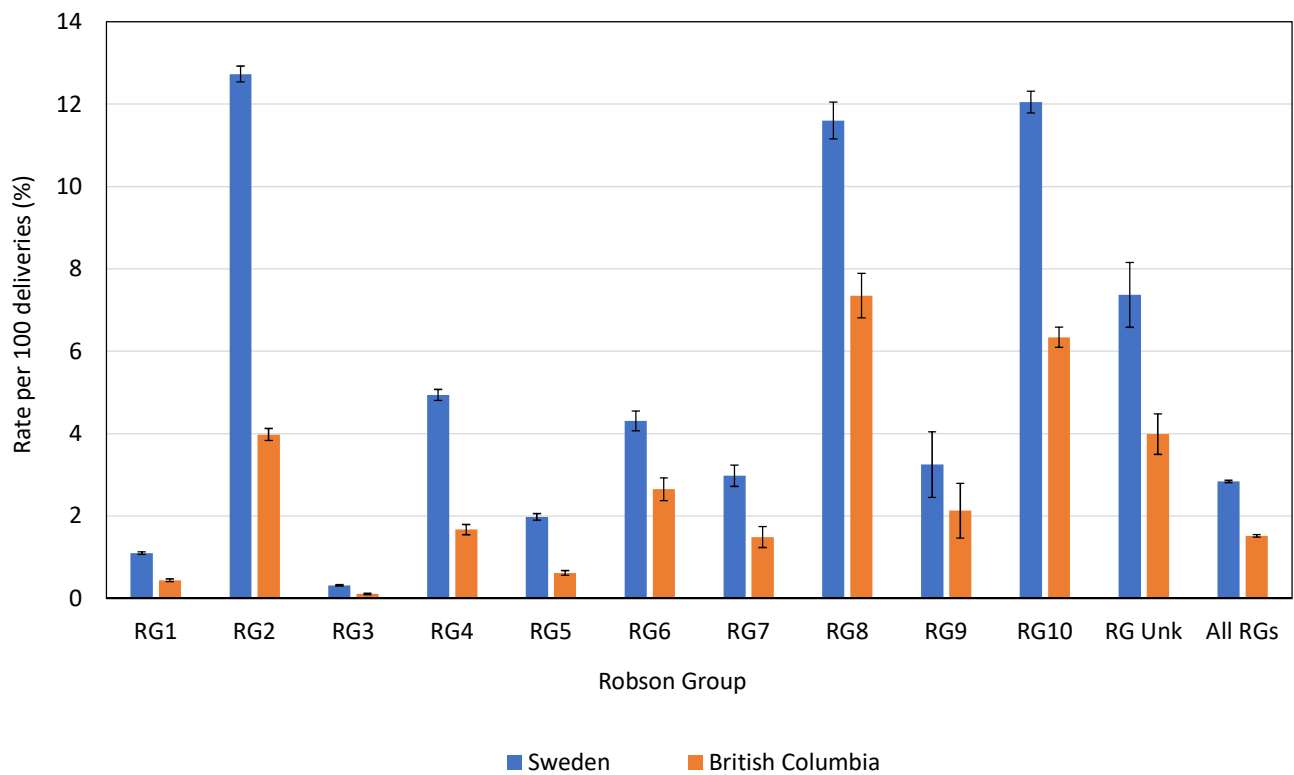

Supplement: S1 Fig — Proportion of women diagnosed with preeclampsia/eclampsia stratified by country and Robson group. Sweden is represented in the blue bars and BC is represented in the orange bars. The error bars indicate the 95% confidence interval. “RG” denotes Robson group, “Unk” denotes unknown. (PDF) [file pmed.1004077.s027.pdf]
