# Supplementary material for: Crude and adjusted comparisons of cesarean delivery rates using the Robson classification: A population-based cohort study in Canada and Sweden, 2004 to 2016
Source: PLoS Med. 2022 Aug 1;19(8):e1004077. doi: 10.1371/journal.pmed.1004077 (PMC9377587; doi:10.1371/journal.pmed.1004077)

S2 Fig.
Temporal trends in maternal characteristics, obstetric practice factors and fetal/infant characteristics among women in **Robson Group 1**, Sweden and British Columbia, 2004-2016

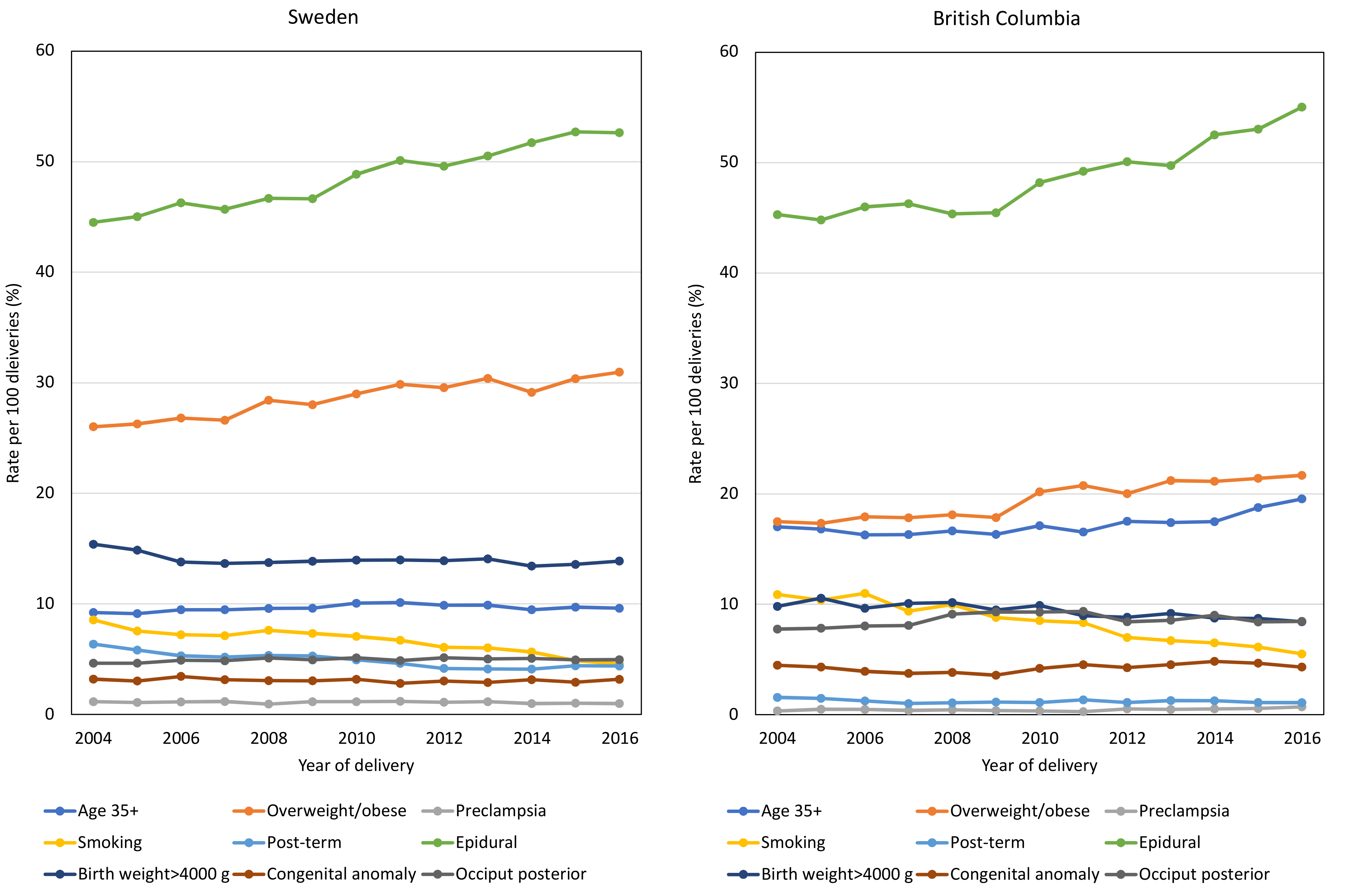

Supplement: S2 Fig — Changes in the frequency of determinants of cesarean delivery over the study period in Robson Group 1. (PDF) [file pmed.1004077.s028.pdf]
