# Supplementary material for: Crude and adjusted comparisons of cesarean delivery rates using the Robson classification: A population-based cohort study in Canada and Sweden, 2004 to 2016
Source: PLoS Med. 2022 Aug 1;19(8):e1004077. doi: 10.1371/journal.pmed.1004077 (PMC9377587; doi:10.1371/journal.pmed.1004077)
Supplement: Attachment — Submitted filename: TGCS a new way of thinking.pdf [file pmed.1004077.s031.pdf]

The 10-Group Classification System—a new way of thinking

Michael S. Robson, MBBS, MRCOG, FRCS, FRCPI

General message of the article

In this edition of the Journal, Hehir and colleagues have applied the 10-Group Classification System (TGCS)[1] to routinely collected US national birth data (2005 through 2014). The authors hypothesize that if hospital and health organizations used the TGCS it would allow standardized comparisons of cesarean delivery (CD) rates by identifying prospectively clinically distinct groups of women that are driving CD rates.[2,3] It would also allow for a more targeted evaluation of quality and the processes associated with CD in these different groups.

Their main clinical finding from this large national database was similar to other studies.[4] Women with a previous CD at ≥37 weeks with a single cephalic fetus (group 5) represent the largest single contributor to the overall CD rate. It is also the group whose contribution continues to increase. This is mainly due to an increase in the size of the group rather than an increase in the CD rate in the group.

The authors also question the possibility of misclassification of women because of the very high contribution of prelabor CD in women with a single cephalic presentation at ≥37 weeks' gestation but without a previous scar (groups 2b and 4b). Finally, they confirm that although groups 6—10 have high CD rates, because of their small size their contribution to the overall CD rate will always be minimal.

From these observations alone, the authors begin to ask themselves questions and quickly identify the need for more detailed but specific information to confirm or refute their hypotheses. The TGCS was on its own never intended to answer all questions. The processes that vary between labor and delivery units such as diagnosis of labor, rupture of membranes, diagnosis and treatment of dystocia, diagnosis of fetal distress, use of epidural analgesia, induction of labor, and many others are all important to understand when interpreting the TGCS results in a particular data set. The authors comment that the TGCS does not include indications for CD and they are correct. An objective classification of indications for CD is also required within the TGCS and a classification that uses the same principles as the TGCS of grouping women using objective criteria has been implemented successfully.[5]

From this article it becomes clear that the TGCS represents a new way of thinking. It is not just a classification of CD or a useful way in focusing on how to reduce CD rates.[6] It provides a common starting point[7] for further analyses for all labor and delivery events and outcome and its principles of simplicity and clarity of thought help to stimulate interest, discussion, and education.

Importantly, it is presented in such a way that all clinicians, whatever their academic or professional background, can identify with it, encouraging long-term commitment and responsibility especially if shared nationally and internationally. A manual has been produced by the World Health Organization (WHO) to help implement and interpret the TGCS.[8]

The scientific merit of the article

The philosophy behind the TGCS and the methodology for implementing the TGCS has been well described elsewhere.[8–10] However, there are a few suggestions that might enhance the message from this article.

It is important that the obstetric terminology used is standardized especially if the results are to be consistently interpreted. The term "nulliparous" rather than "primiparous" should be used as they strictly speaking refer to different groups of women.[11]

It is crucial that the initial TGCS table is presented in a simple, standard, and consistent manner both in the literature and in slide presentations. The recommended table is presented in this editorial as a Figure and has been constructed from the 2014 data taken from Table 2 in the article. It differs from those tables in the article in 2 important aspects.

Firstly, and only for the initial overview of the results, groups 2a and 2b are amalgamated into 1 group as are 4a and 4b. At first sight this seems to be less informative but experienced users of the system will argue the opposite.

Secondly, the contribution to the overall CD rate by each of the groups in column 5 of the table is calculated in absolute percentages of the total population rather than the relative proportion of the number of CDs. This again will give the reader more instant information; otherwise significant trends could be missed. The detailed reasoning behind both of these suggestions is described elsewhere.[8] The raw numbers of the total number of deliveries as well as CDs in the individual groups should always be available. The relative sizes of the

From the National Maternity Hospital, Dublin, Ireland.

Received May 14, 2018; revised May 22, 2018; accepted May 22, 2018.

The author reports no conflict of interest other than his name having been attached to the classification and that he was responsible for constructing it. There are no commercial benefits to the author. The author does receive invitations to scientific congresses to talk to clinicians about the use of the classification in clinical practice and organization of training courses.

Corresponding author: Michael S. Robson, MBBS, MRCOG, FRCS, FRCPI. mrobson@nmh.ie

0002-9378/free
© 2018 Elsevier Inc. All rights reserved.
https://doi.org/10.1016/j.ajog.2018.05.026

Related article, page 105.

FIGURE
Cesarean sections using the 10-group classification system, 2014

Caesarean Sections - the 10 Groups 2014

	2014 US 1164954/3722031 31.3%	Size of group %	C/S rate in gp %	Contr of each gp 31.3 %
**1** Nullip single ceph >=37 wks spon lab	78149/635436	17.1	12.3	2.1
**2** Nullip single ceph >=37wks ind. or CS before lab	177749/398243	10.7	44.6	4.8
**3** Multip (excl prev caesarean sections) single ceph >=37 wks spon lab	50615/1152360	31.0	4.4	1.4
**4** Multip (excl prev caesarean sections) single ceph >=37wks ind. or CS before lab	238184/672271	18.1	35.4	6.4
**5** Previous caesarean section single ceph >= 37 wks	401495/457254	12.3	87.8	10.8
**6** All nulliparous breeches	13357/13813	0.4	96.7	0.4
**7** All multiparous breeches (incl previous caesarean sections)	40906/44265	1.2	92.4	1.1
**8** All multiple pregnancies (incl previous caesarean sections)	67707/98026	2.6	69.1	1.8
**9** All abnormal lies (incl previous caesarean sections)	4500/6678	0.2	67.4	0.1
**10** All single ceph <= 36 wks (incl previous caesarean sections)	92292/243685	6.6	37.9	2.5

Robson. The 10-Group Classification System–a new way of thinking. Am J Obstet Gynecol 2018.

groups should always be ascertained before the CD rates are analyzed.

Finally, this study refers to a large national database (National Vital Statistics System) and according to the results presented, each woman was able to be classified into one of the groups. This is an unusual finding in a large database and normally there is always a small percentage of women that are not classifiable due to a lack of information. It is always important to record the number and percentage of women that could not be classified as this is an important data quality indicator for the whole cohort and also a pointer to where data quality improvements could be made. If all the women were classifiable this would be worth commenting on in the text.

How this article compares to other similar information published

This article will hopefully be the first of many from the United States on the TGCS. The comparison with similar information published elsewhere[12,13] is important to both improve data quality and stimulate questions and discussion.

In comparing results, it is always important to think of the 3 reasons why there may be a difference in either the sizes of the groups or any of the events and outcomes within the groups. All labor and delivery events and outcomes can be classified in the TGCS.

Firstly, consider data quality. This may be data definition issues or data collection issues, both of which affect our ability to obtain good routinely collected data. Secondly, there may be significant differences in epidemiological variables. Lastly, if both of these have been accounted for then the only remaining reason is a difference in practice.

Table 2 from the Hehir et al article containing data from 2014 was reconstructed as previously described and the results interpreted in line with previously published data using the TGCS.

In this national data set the proportion of nulliparous women was lower than other countries at 31.4%.

The size of group 5 at 12.3% is lower than expected and suggests that some women with a previous CD scar could have been misclassified elsewhere.[8]

The total breech population (groups 6 and 7) is lower than expected. In a population with a high preterm delivery rate (6.6% in group 10) to have a total incidence of breech deliveries of 1.6% and, particularly, 0.4% nulliparous breeches is unusually low and suggests that some women with a breech presentation have been misclassified elsewhere.

The size of group 8 at population level is also quite high at 2.6% for population data.

Group 9 is classically very consistent in size usually between 0.2–0.6%. This serves as a data quality indicator but the CD delivery rate should always be 100% because by convention all transverse lies need CD and in the article the CD delivery rate was 67.4%.

The CD rates in groups 2 and 4 are significantly higher than commonly seen. The reason for this is a high incidence of prelabor CDs and misclassification needs to be ruled out.

The ratio of groups 1–2 and 3–4 is an immediate appreciation of the rate of intervention of term, singleton, cephalic pregnancies (without a CD scar) by induction or prelabor CD.[3] The ratio is always less in nulliparous women as compared with multiparous women in the same data set and this is confirmed in this article but in absolute terms will vary in different populations. The most important influencing factor affecting the relative sizes of the groups are the definitions of spontaneous and induced labor and prelabor CD. These groups are mutually exclusive but are difficult to collect accurately. National data from the United Kingdom have shown that routine data quality issues in the onset of labor and delivery and parity are not uncommon issues with up to 24% and 49% of hospitals failing, respectfully.[14] They also note that some primiparous women were being incorrectly recorded as multiparous women, which is difficult to understand. The TGCS is an easy way to directly validate these data because of its structure.

The potential importance of the topic

The background behind this article is the continued debate about CD rates. Although much has been written it is difficult to conclude that much progress has been made or consensus achieved. Whatever about the justifications of different CD rates, we remain without an internationally recognized classification system[15] to scientifically study the effects of rising CD rates. Ultimately in the future it will be this failure rather than the increase in the CD rate itself that will be most critically challenged. Safety and quality are the most important outcomes and no event or outcome, particularly CD, should ever be interpreted in isolation from any other outcome.

Labor and delivery is a complex process. At the present time there are 2 competing but ultimately complimentary philosophies to evidence-based care: randomized controlled trials (RCTs) and perinatal audit. RCTs are considered the highest form of evidence and undoubtedly often are. However, in complex situations such as labor and delivery, perinatal audit in an organized and structured way with adequate training may become more useful.

Interestingly, organizations outside medicine (business or sport) that are also trying to achieve and maintain quality depend more on analyzing their routine data. These organizations are rapidly developing and improving their methods of routine data collection and, in particular, the analysis of their data using sophisticated analytical systems. There is no doubt that there are lessons that could be learned here.

Ultimately the solution will be an electronic patient record that incorporates all aspects of patient care including all clinical documentation, order communication, medications, anesthesia, and theater. It will be a system primarily designed to make the patient journey both easier and safer by improving communication, access to the chart, and legibility. However, it also will provide for the better collection of routine data, both clinical and organizational, with the potential for designing the most cost-effective models of care and establishing an epidemiological database of which we can be proud. In Ireland, a national strategy has already started to implement an integrated national maternity and neonatal electronic record.

The main message of this editorial is that perinatal audit and labor and delivery audit,[16] in particular, is not universally recognized as a scientific entity, specialized area, or even at all useful. It is the poor relation of RCTs. Collection of routine data is resource dependent requiring total organizational commitment. At the moment there are no accepted terminology, classifications, core event and outcome definitions, principles, or training programs.

Once consistent, useful, and validated information is available, multidisciplinary quality assurance programs should be developed in all labor and delivery units.[9,10] The TGCS is an attempt to start this process by providing a common initial pathway that has now been used and published in >50 countries. It has been endorsed by the WHO,[17] International Federation of Gynecology and Obstetrics,[18] and European Board and College of Obstetrics and Gynecology,[19] but will not reach its full potential until it is supported by national professional organizations and routinely used by all labor and delivery units. ■

REFERENCES

1. Robson MS. Classification of cesarean sections. Fetal Matern Med Rev 2001;12:23–39.
2. Brennan D, Robson M, Murphy M, O'Herlihy C. Comparative analysis of international cesarean delivery rates using 10-group classification identifies significant variation in spontaneous labor. Am J Obstet Gynecol 2009;201:308.e1–8.
3. Brennan D, Murphy M, Robson M, O'Herlihy C. The singleton, cephalic nulliparous woman after 36 weeks of gestation: contribution to overall cesarean delivery rates. Obstet Gynecol 2011;117:273–9.

4. Pyykönen A, Gissler M, Løkkegaard E, et al. Cesarean section trends in the Nordic countries—a comparative analysis with the Robson classification. Acta Obstet Gynecol Scand 2017;96:607—16.
5. Campbell S, Murphy M, Keane DP, Robson M. Classification of intrapartum cesarean delivery: a starting point for more detailed analysis. Am J Obstet Gynecol 2017;216:S245—6.
6. Boatin AA, Cullinane F, Torloni MR, Betran AP. Audit and feedback using the Robson classification to reduce cesarean section rates: a systematic review. BJOG 2018;125:36—42.
7. Robson M. The Ten Group Classification System (TGCS)—a common starting point for more detailed analysis. BJOG 2015;122:701.
8. Robson classification: implementation manual. Geneva (Switzerland): World Health Organization; 2017.
9. Robson M, Hartigan L, Murphy M. Methods of achieving and maintaining an appropriate cesarean section rate. Best Pract Res Clin Obstet Gynaecol 2013;27:297—308.
10. Robson M, Murphy M, Byrne F. Quality assurance: the 10-Group Classification System (Robson classification), induction of labor, and cesarean delivery. Int J Gynecol Obstet 2015;131(Suppl):S23—7.
11. Hughes G. Is this primip a nullip? The daily abuse of language in obstetrics. BJOG 2018 Jan 19. https://doi.org/10.1111/1471-0528.15138. [Epub ahead of print].
12. National Maternity Hospital. Annual Clinical Report. Dublin (Ireland): National Maternity Hospital; 2016.
13. Swedish Federation of Obstetrics and Gynecology. Obsterics in Sweden using the Robson 10-groups classification. Available at: https://www.sfog.se/start/10-groups/. Accessed June 7, 2018.
14. London School of Hygiene and Tropical Medicine. Royal College of Obstetricians and Gynecologists. Patterns of maternity care in English NHS trusts 2013/14. March 2016. Chapter 4. Data Quality 35—42.
15. Torloni MR, Betran AP, Souza JP, et al. Classifications for cesarean section: a systematic review. PLoS One 2011;6:e14566.
16. Robson MS. Labor ward audit. In: Creasy RK, ed. Management of labor and delivery. Oxford: Blackwell Science; 1997:559—70.
17. Betran AP, Torlini MR, Zhang JJ, Gulmezoglu AM; for the Working Group on Cesarean Section. WHO statement on cesarean section rates. BJOG 2016;123:667—70.
18. FIGO Working Group on Challenges in Care of Mothers and Infants During Labor and Delivery. FIGO statement. Best practice advice on the 10-Group Classification System for cesarean deliveries. Int J Gynecol Obstet 2016;135:232—3.
19. European Board and College of Obstetrics and Gynecology. EBCOG position statement on cesarean section in Europe. Eur J Obstet Gynecol Reprod Biol 2017;219:129.
