# Supplementary material for: Crude and adjusted comparisons of cesarean delivery rates using the Robson classification: A population-based cohort study in Canada and Sweden, 2004 to 2016
Source: PLoS Med. 2022 Aug 1;19(8):e1004077. doi: 10.1371/journal.pmed.1004077 (PMC9377587; doi:10.1371/journal.pmed.1004077)
Supplement: Attachment — Submitted filename: Known knowns, unknown unknowns a.pdf [file pmed.1004077.s032.pdf]

BJOG An International Journal of Obstetrics and Gynaecology
Royal College of Obstetricians & Gynaecologists

DOI: 10.1111/1471-0528.16679
www.bjog.org

Mini commentary

Known knowns, unknown unknowns and everything in-between – the Ten Group Classification System (TGCS)

Michael S Robson

The National Maternity Hospital, Dublin 2, Ireland

Linked article: This is a mini commentary on J Zeitlin et al. To view this article visit https://doi.org/10.1111/1471-0528.16634

Providing quality evidence-based perinatal care depends on determining best processes and auditing results. Business and sport rely more on the latter, adapting processes accordingly. Routine data collection, a prerequisite to audit, remains neglected in healthcare, but its importance is no better exemplified than by the current Covid epidemic.

Zeitlin (*BJOG* 2021; https://doi.org/10.1111/1471-0528.16634) demonstrates that perinatal audit is not recognised as a speciality. No consistent definitions or classifications are used and there is no formal training programme. There is no commitment at professional or government level to reward quality information. The diversity of sources of information used in the article masks the responsibilities that electronic patient record vendors and individual clinicians have. Clinical information needs to be easily collected and reported on, and documented in a disciplined manner.

It is crucial to appreciate that all data need validation. Known knowns must be actually what they seem and not unknown unknowns (things that we don't know we don't know).

The Ten Group Classification System (TCGS) was originally constructed as a perinatal classification system for analysing all perinatal events, outcomes, satisfaction and complications (short and long term) including anaesthesia, neonatal and pathology. The starting ten groups, its subgroups and certain amalgamated groups are remarkably consistent in size between organisations and any variation is explained most commonly by data quality; only after that, should significant epidemiological variables or differences in clinical practice be considered (Robson. *AJOG* 2018;219:1–4) Furthermore because the groups are relatively homogeneous obstetrically, measuring certain clinical events within the groups such as the use of oxytocin or caesarean section rate (CSR) can further validate the data.

Once the TGCS has been embedded into routine data collection, progress can be made in collecting and analysing the known unknowns (things we know we don't know) (Robson. *BJOG* 2015;122:701). These will be unique in terms of incidence and clinical significance within the TGCS and, once collected within the TGCS, data validation and interpretation becomes easier, more relevant and more rewarding. This has implications for clinical practice, as the TGCS is based on prospectively identifiable groups so lessons can be quickly applied.

The true unknown unknowns (undiscovered things that we don't know we don't know) will hopefully become apparent in time either by chance or intuition after implementation of the TGCS.

In this paper, Zeitlin analyses CSR using the TGCS (*Implementation Manual*. WHO 2017; Licence CC BY-NC-SA 3.0 IGO). For this controversial debate to achieve any consensus we need to confirm what we think we know and then decide what we need to know. It must include other events and outcomes apart from CSR (Kempe. *EJOG* 2019;237:181–8). Currently, this concept has singularly failed and no consensus will be possible until it is resolved.

The most fundamental measure of quality care is knowing your results, including the ability to interpret them. It is time to acknowledge different ways of providing care and, rather than concentrating on standardizing processes, standardize the way we carry out perinatal audit so greater comparison and learning can take place between delivery units.

© 2021 John Wiley & Sons Ltd.

Ultimately, this is the only way we will be able to ensure that appropriate care is being given.

Disclosure of interests

A completed disclosure of interests form is available to view online as supporting information.

Funding

There are no funders to report for this submission.

Data availability statement

Data sharing not applicable to this article as no datasets were generated or analysed during the current study.

Supporting Information

Additional supporting information may be found online in the Supporting Information section at the end of the article. ■

© 2021 John Wiley & Sons Ltd.
