# Supplementary material for: Crude and adjusted comparisons of cesarean delivery rates using the Robson classification: A population-based cohort study in Canada and Sweden, 2004 to 2016
Source: PLoS Med. 2022 Aug 1;19(8):e1004077. doi: 10.1371/journal.pmed.1004077 (PMC9377587; doi:10.1371/journal.pmed.1004077)
Supplement: Attachment — Submitted filename: renamed_e5be2.pdf [file pmed.1004077.s033.pdf]

DOI: 10.1111/1471-0528.13267
www.bjog.org

Mini Commentary

The Ten Group Classification System (TGCS) – a common starting point for more detailed analysis

M Robson

National Maternity Hospital, Dublin, Ireland

Published Online 20 January 2015.

Linked article: This is a mini commentary on Le Ray C et al. To view this article visit http://dx.doi.org/10.1111/1471-0528.13199.

No classification system has been formally introduced to study caesarean sections scientifically together with any ensuing consequences for mother or baby. In the future it will be this failure by clinicians that will be most vulnerable to criticism.

The TGCS is a prospective, standardised structure of clinically relevant groups of women allowing for a more constructive debate on caesarean sections (Robson *Fetal Matern Med Rev* 2001;12:23–39). The TGCS serves as the initial structure within which additional epidemiological variables, processes, perinatal events and outcomes in addition to caesarean sections can be analysed.

Often misunderstood and incompletely interpreted, the TGCS was never meant to be the endpoint of caesarean section audit, merely the starting point. Complicating a simple classification system at the outset with detailed subgroups and multiple variables discourages universal adoption and implementation. On its own, however, the TGCS of caesarean sections still provides a useful and easy tool for analysing the clinical activity and philosophy of care of an individual care group, organisation, region or country.

The TGCS does not explain why caesarean sections are done but it does allow an objective, common starting point to investigate the reasons. Further classification of indications for caesarean sections within the groups is needed (Robson M. *Best Pract Res Clin Obstet Gynaecol* 2013;27:297–308). Any resultant changes in other perinatal outcomes as an effect of altering clinical practice can be monitored easily. The full potential of the TGCS will only be realised when it is adopted as standard practice, enabling clinicians to learn from each other. This may be sooner rather than later and it may be women and governments that request it rather than professional bodies. Its simplicity is such that the information needed to identify the group to which the woman belongs can be given by the woman herself, allowing the system to be both robust and universal. Despite this, many institutions and countries remain unable to publish their results because of poor quality data collection. Indeed one of many unexpected benefits of using the TGCS has been to assess data quality.

This paper published in the *BJOG* is a national study of caesarean section rates over 1 week in three different epochs: 1995, 2003 and 2010. It uses the TGCS and shows a low overall caesarean section rate. It also highlights, as many others have previously, the importance of labour and delivery in the nulliparous women with a single cephalic term pregnancy. These data are useful references for other institutions to compare their own data with. However, more detailed data within each of the groups, their subgroups or amalgamation of some groups are needed to facilitate the debate on caesarean section. In particular, short- and long-term information on fetal but also maternal outcome is required. Efforts should be made to ensure that this data is collected routinely, validated locally and available in national databases.

Disclosure of interests

Nothing to disclose. ■

© 2015 Royal College of Obstetricians and Gynaecologists
