# Supplementary material for: Crude and adjusted comparisons of cesarean delivery rates using the Robson classification: A population-based cohort study in Canada and Sweden, 2004 to 2016
Source: PLoS Med. 2022 Aug 1;19(8):e1004077. doi: 10.1371/journal.pmed.1004077 (PMC9377587; doi:10.1371/journal.pmed.1004077)
Supplement: Attachment — Submitted filename: Response to Review 23may22.docx [file pmed.1004077.s034.docx]

**Requests from the Editors**

1. General comments: Throughout the paper, please adapt reference call-outs to the following style: "... gestational age (<37 vs ≥37 weeks) [8,9]." (noting the absence of spaces within the square brackets).

***Response***: The reference call-outs have been revised throughout the paper.

2. Data availability: The Data Availability Statement (DAS) requires revision. If the data are owned by a third party but not freely available upon request, please state the owner of the data set and contact information for data requests (web or email address). Note that a study author cannot be the contact person for the data.

***Response***: The Data Availability statement has been revised as follows (lines 554-565):

“The Swedish Medical Birth Registry is a national dataset and considered to be public property. However, access to the data is given only to researchers with permission from the Swedish Ethical Review Authority and after approval of the research plan by a Swedish National Board of Health and Welfare data manager. The data request may be sent to The Swedish National Board of Health and Welfare (<https://www.socialstyrelsen.se/>). The Canadian data were acquired from Perinatal Services British Columbia. Access to the Canadian data is available to researchers, health managers and decision-makers via a formal data request process administered by Perinatal Services British Columbia. More information on the data holdings and the request process is available at <http://www.perinatalservicesbc.ca/our-services/data-surveillance/perinatal-data-registry/data-requests>.”

3. Title: Please revise your title according to PLOS Medicine's style. We suggest “Adjusted comparisons of cesarean delivery rates between populations using the Robson classification: A population-based cohort study in Canada and Sweden, 2004-2016” or similar

***Response***: The title has been revised as suggested. It now reads:

“Crude and adjusted comparisons of cesarean delivery rates using the Robson classification: A

population-based cohort study in Canada and Sweden, 2004-2016”.

4. Abstract: Please structure your abstract using the PLOS Medicine headings (Background, Methods and Findings, Conclusions).

***Response***: The abstract now reflects the *PLOS Medicine* headings.

5. Abstract Background:
   a. The final sentence should clearly state the study question.

***Response***: In the revised manuscript, the final sentence of the Abstract Background now clearly articulates the study questions. “The objectives of our study were to identify subgroups of women contributing to differences in the CD rate in Sweden and British Columbia (BC), Canada using the Robson classification, and to estimate the contribution of maternal characteristics, maternal comorbidity, fetal characteristics, and obstetric practice factors to differences in CD rates between countries and over time.” (lines 38-42)

b. Line 36 - ‘cesarean delivery’ is duplicated

***Response***: This error has been corrected.

6. Abstract Methods and Findings:
   a. Please provide brief demographic details of the study population (e.g. fetal sex, maternal age, ethnicity, etc).

***Response***: The following demographic details are included in the revised Abstract (lines 53-55):

“Nulliparous women comprised 44.8% of the study population, while women of advanced maternal age (≥35 years), and overweight/obese women (≥25 kg/m2) constituted 23.5% and 32.4% of the study population, respectively.”

b. Please also specify that data used in the analysis are from January 1, 2004 and December 31, 2016

***Response***: We now specify the dates for which the data was obtained.

Lines 45-46: “Population-based cohort study of deliveries in Sweden (January 1, 2004-December 31, 2016) and BC, Canada (March 1, 2004–April 31, 2017; n=1,951,984).”

c. Please define CD and BC at first use

***Response***: CD and BC are now defined in the Abstract at first use on lines 36 and 39, respectively.

d. Please define Group 2

***Response***: Group 2 is now defined. Lines 60-61: “…and in Group 2 (nullipara, single, cephalic fetus, term gestation with induction of labour or CD prior to labour) the rate of CD was 37.3% in Sweden and 45.9% in BC (RR=1.23, 95% CI 1.22–1.25).”

e. Please include the most important variables that are adjusted for in the analyses.

***Response***: The Methods and Findings section of the Abstract now reads:

“The effect of adjustment for maternal characteristics (e.g., age and body-mass-index), maternal comorbidity (e.g., preeclampsia), fetal characteristics (e.g., position of the fetal head), and obstetric practice factors (e.g., epidural anesthesia) ranged from no effect (e.g., among deliveries with breech presentation; Groups 6, 7) to explaining up to 5.2% of the absolute 8.6% difference in CD rate (Group 2: adjusted CD rate in BC 40.7%, adjusted RR=1.09, 95% CI 1.08–1.12).” (lines 62-66)

f. Please include the actual amounts and/or absolute risk(s) of relevant outcomes, not just RRs.

***Response***: The Abstract now includes the absolute risk(s) as well as the relative estimates on lines (56-62). Differences in CD rates between Sweden and BC varied by Robson Group, for example, in Group 1 (nullipara with a term, single, cephalic fetus with spontaneous labour), the CD rate was 8.1% in Sweden and 20.4% in BC (rate ratio [RR] for BC vs Sweden=2.52, 95% confidence interval [CI] 2.49–2.56) and in Group 2 (nullipara, single, cephalic fetus, term gestation with induction of labour or CD prior to labour) the rate of CD was 37.3% in Sweden and 45.9% in BC (RR=1.23, 95% CI 1.22–1.25).” As well as on lines 65-66: “…explaining up to 5.2% of the absolute difference in CD rate (Group 2: adjusted CD rate in BC 40.7%, adjusted RR=1.09, 95% CI 1.08–1.12)”.

g. In the last sentence of the Abstract Methods and Findings section, please describe 2-3 of the main limitations of the study's methodology.

***Response***: The last sentence of the Abstract Methods and Findings now reads:

“Limitations of the study include a lack of information on intrapartum details, such as duration of labour and oxytocin augmentation as well as maternal, and perinatal outcomes associated with the observed differences in CD rates.” (lines 68-70)

7. Abstract Conclusions: Please begin your Abstract Conclusions with "In this study, we observed ..." or similar, to summarize the main findings from your study, without overstating your conclusions. Please emphasize what is new and address the implications of your study, being careful to avoid assertions of primacy.

***Response***: The Conclusions section of the Abstract now reads: “In this study, we found that maternal, fetal and obstetric practice factors not included in the Robson classification explain a significant proportion of the spatio-temporal difference in cesarean delivery rates in some Robson Groups, and need to be incorporated into explanatory models evaluating cesarean delivery rates in populations.” (lines 73-76).

8. Author Summary: At this stage, we ask that you include a short, non-technical Author Summary of your research to make findings accessible to a wide audience that includes both scientists and non-scientists. The Author Summary should immediately follow the Abstract in your revised manuscript. This text is subject to editorial change and should be distinct from the scientific abstract. Please see our author guidelines for more information: <https://journals.plos.org/plosmedicine/s/revising-your-manuscript#loc-author-summary>. In the final bullet point of ‘What Do These Findings Mean?’, please describe the main limitations of the study in non-technical language.

***Response:*** The Author Summary has been included following the Abstract on pages 6-7.

9. Methods:
   a. Did your study have a prospective protocol or analysis plan? Please state this (either way) early in the Methods section. If a prospective analysis plan (from your funding proposal, IRB or other ethics committee submission, study protocol, or other planning document written before analyzing the data) was used in designing the study, please include the relevant prospectively written document with your revised manuscript as a Supporting Information file to be published alongside your study, and cite it in the Methods section. A legend for this file should be included at the end of your manuscript. If no such document exists, please make sure that the Methods section transparently describes when analyses were planned, and if/when reported analyses differed from those that were planned. Changes in the analysis-- including those made in response to peer review comments-- should be identified as such in the Methods section of the paper, with rationale. If a reported analysis was performed based on an interesting but unanticipated pattern in the data, please be clear that the analysis was data-driven.

***Response***: The prospectively written protocol has been included as a Supporting Information file titled ‘S1 Text’. This has been included in the first paragraph of the Methods section (lines 155-156):

“The study methodology and analysis plan (S1 Text) were developed prior to data manipulation and the actual analysis.” A legend for Supporting Information has been included at the end of the revised manuscript.

b. Thank you for providing a STROBE checklist as Supporting Information. Please add the following statement, or similar, to the Methods: "This study is reported as per the Strengthening the Reporting of Observational Studies in Epidemiology (STROBE) guideline (S1 Checklist)." The STROBE guideline can be found here: <http://www.equator-network.org/reporting-guidelines/strobe/> When completing the checklist, please use section and paragraph numbers, rather than page numbers which will likely no longer correspond to the appropriate sections after copy-editing.

***Response:*** We have included the statement above in lines 154-155 at the beginning of the Methods section. Section and paragraph numbers have been used to signpost the checklist as suggested.

c. Line 308 - Please replace "subject" with participant, patient, individual, or person.

***Response***: The term “subjects” has been replaced with “individuals” on line 366.

10. Results:
    a. Please incorporate table S4 into the main paper as Table 1, to show the baseline characteristics of the study population.

***Response***: Table S4 is now incorporated in the main paper as Table 1. (pages 30-31)

b. Line 258 - Please revise use of ‘significant’ unless statistical analyses are provided to substantiate statistical significance

***Response***: We have removed the term ‘significant’ in this sentence. (line 314)

11. Discussion:

Please rename your ‘Comment’ section to ‘Discussion’, and remove all subheadings e.g. ‘Principal findings’ and ‘Conclusion’

Please present and organize the Discussion as follows: a short, clear summary of the article's findings; what the study adds to existing research and where and why the results may differ from previous research; strengths and limitations of the study; implications and next steps for research, clinical practice, and/or public policy; one-paragraph conclusion.

***Response***: The Discussion section has been restructured as suggested.

12. Tables:

Please define all abbreviations used in the table legend of each table.

***Response***: All abbreviations used in the tables have been defined in each respective table legend.

13. References:

Please ensure that journal name abbreviations match those found in the National Center for Biotechnology Information (NCBI) databases (<http://www.ncbi.nlm.nih.gov/nlmcatalog/journals>), and are appropriately formatted and capitalised. Please also name 6 authors prior to ‘et al’.

Where website addresses are cited, please specify the date of access.

***Response***: The References have been revised as requested.

**Comments from the reviewers:**

**Reviewer #1:** Thanks for the opportunity to review your manuscript. My role is as a statistical reviewer so my comments focus on the study design, data, and analysis presented in the manuscript. I have put general comments first, and followed these with queries relevant to specific section of the manuscript (with a line reference).

This study uses data pooled from national data from Sweden and province level data from BC in Canada (from the birth registration systems). This manuscript evaluates if the standard for classification of deliveries used for comparing caesarean delivery rates can be improved by considering other maternal/fetal/health service characteristics that could explain variation in CDR. The study is large (~2 million deliveries) and aside from a few variables the data appears fairly complete. There are some queries below but the manuscript is written clearly and addresses an interesting question.

**Comment 1**:My background is not in working in perinatal epidemiology, and I was curious about how much of the overall rates in CD appear to come from a few specific Robson groups. Does the higher rates reflect a genuine healthcare need for these mothers, or is it from failure of the healthcare services to avoid an unnecessary CD? (this is question much more motivated by my own interest rather than a need to change the manuscript)

**Response**: This is the “million dollar question” and the reason we have yet to find the answer is due to a combination of ideologic and methodologic challenges outlined below.

There is considerable debate surrounding what constitute genuine indications for CD. For some indications, there is absolute agreement on necessity, for example complete placenta previa or placenta accreta [1]. Yet, many indications are highly debated, such as a previous CD [2-4], advanced maternal age [5], and obesity [6]. And perhaps the most contested indication of all is maternal preference, often labelled CD *without* indication [7-9]. While this may seem intuitively to fall in the “unnecessary” category, there are strong arguments that centre on issues of women’s autonomy and the legacy of paternalistic practice in obstetrics that assert that it is individual women, with informed consent, who should determine what is considered necessary vs unnecessary based on personal valuation of risks/benefits [10-12].

One way to address this subjectivity is to produce evidence that maternal and/or perinatal outcomes favour CD compared with alternative modes of delivery for specific indications. Randomized controlled trials (RCTs) have been carried out for specific indications, such as breech fetal presentation [13] and multiple gestation (twins) [14]; however, randomizing women to planned CD or planned vaginal delivery is sometimes deemed infeasible or unethical [15]. Furthermore, observational studies aiming to estimate these relationships using individual-level data are hindered by confounding by indication, since even within groups restricted to one indication, the severity of indication is difficult to quantify [16].

In light of these methodologic challenges, ecologic designs present an opportunity to study CD rates without the feasibility and ethical issues that preclude experimentation or biases such as confounding by indication [17]. Ecologic studies can quantify the relationship between CD rates and maternal and neonatal outcomes in an effort to get closer to an evidence-based estimate of the optimal rate of CD. Such studies have shown that the threshold for the inverse ecologic relationship between CD rate and mortality outcomes (maternal, neonatal and infant mortality) appears to be between 9%-16% [18]. Rates of CD above this threshold are not associated with further reductions in mortality. The major limitations of these findings in answering the question regarding optimal CD rates are: a) these studies propose an optimal CD rate for the entire obstetric population, without accounting for the distribution of risk factors and b) only mortality has been studied; mortality is rare in high-income countries and rates of severe morbidity are more relevant and informative measures of maternal care quality. The associations between CD rates and relevant morbidity outcomes have yet to be evaluated before we may draw more definite conclusions or appropriate CD rates on a population level [19].

In summary, the answer to your question is not straightforward. Defining a genuine need or indication for cesarean delivery is difficult, with several controversies regarding what is considered a legitimate indication for CD. On the population-level, there is little consensus regarding appropriate rates of CD.

References

1. Tita ATN. When is primary cesarean appropriate: maternal and obstetrical indications. Semin Perinatol 2012;36:324-7.
2. Smith GC, Pell JP, Cameron AD, Dobbie R. Risk of perinatal death associated with labor after previous cesarean delivery in uncomplicated term pregnancies. JAMA 2002;287:2684-90.
3. Landon MB, Hauth JC, Leveno KJ, Spong CY, Leindecker S, Varner MW, et al. Maternal and perinatal outcomes associated with a trial of labor after prior cesarean delivery. NEJM 2004;351:2581-9.
4. Young CB, Liu S, Muraca GM, Sabr Y, Pressey T, Liston RM, et al. Mode of delivery following a previous cesarean and associated maternal and neonatal morbidity CMAJ 2018;190:E556-64.
5. Jain V. Elective caesarean section for the mature nullipara?. Journal of obstetrics and gynaecology Canada: J Obstet Gynaecol Can 2012;34:517.
6. Wispelwey BP, Sheiner E. Cesarean delivery in obese women: a comprehensive review. J Matern Fetal Neonatal Med 2013;26:547-51.
7. Morrison J, MacKenzie IZ. Cesarean section on demand. Semin Perinatol 2003;27:20–33.
8. Jain V. Making sense of rising caesarean section rates: caesarean on demand is obstetric dilemma. BMJ 2004;329:1240.
9. Ecker J. Elective cesarean delivery on maternal request. JAMA 2013;309:1930-6.
10. Bergeron V. The ethics of cesarean section on maternal request: a feminist critique of the American College of Obstetricians and Gynecologists' position on patient‐choice surgery. Bioethics 2007;21:478-87.
11. Williams HO. The ethical debate of maternal choice and autonomy in cesarean delivery. Clin Perinatol 2008;35:455-62.
12. Burcher, P. The Changing Ethical Landscape of Cesarean Delivery on Maternal Request. In: Lonchyna, VA, Kelley, P, Angelos, P. (eds) Difficult Decisions in Surgical Ethics. 2022;Springer, Cham.
13. Hannah ME, Hannah WJ, Hewson SA, et al. Planned caesarean section versus planned vaginal birth for breech presentation at term: A randomized multicenter trial. Lancet 2000;356:1375-83.
14. Barrett JF, Hannah ME, Hutton EK, Willan AR, Allen AC, Armson BA, et al. A randomized trial of planned cesarean or vaginal delivery for twin pregnancy. NEJM 2013;369:1295-305.
15. Baylis F, Halperin SA. Research involving pregnant women: trials and tribulations. Clinical Investigation 2012;2:139-46.
16. Joseph KS, Mehrabadi A, Lisonkova S. Confounding by indication and related concepts. Curr Epidemiol Rep 2014;1:1-8.
17. Wen SW, Kramer MS. Uses of ecologic studies in the assessment of intended treatment effects. J Clin Epidemiol 1999;52:7-12.
18. Betran AP, Torloni MR, Zhang J, Ye J, Mikolajczyk R, Deneux-Tharaux C, et al. What is the optimal rate of caesarean section at population level? A systematic review of ecologic studies. Reprod Health 2015;12:1-0.
19. World Health Organization. WHO Statement on caesarean section rates. 2015 (WHO/RHR/15.02).

**Comment 2**: One thing I think needs some rewording is the description of shift in RR after including additional covariates in the models. Typically ICC (and changes in) would be used a metric of variation and shift in unexplained variation, but to my knowledge this cannot be done with the modified Poisson regression models used. I think the approach of examining how the estimated RRs shift with additional adjustment is reasonable - but I think would be better simply described as absolute shift in estimated rate ratio to the specific Robson group/countries.

***Response***: We have revised our methods to simplify the comparison of crude and adjusted estimates. These changes are reflected in the Methods and Results sections, as well as in Table 3 (previously Table 2).

On lines 216-219 in the Methods section: “The difference between CD rates in the two countries that was explained by differences in risk factors was assessed by comparing the crude RR and adjusted RR (ARR) in each Robson group and on the absolute scale, by comparing the crude and adjusted CD rate in BC vs Sweden.”

On lines 321-325 in the Results: “Adjustment explained 14% of the increase in CD in BC vs Sweden in Robson Group 2 (RR 1.23, 95% CI 1.22–1.25; ARR 1.09, 95% CI 1.08–1.12) and 10.0% of the increase in CD in BC vs Sweden in Robson Groups 5 (RR=1.58, 95% CI 1.57–1.59; ARR=1.48, 95% CI 1.47–1.50), which corresponded to a 5% absolute reduction in the adjusted CD rate in BC in both groups.”

Lastly, in Table 3 on page 33.

**Comment 3**: L55. I am not familiar with the 'sequential' terminology - is this an extension of the Generalised Linear Model?

***Response***: Sequential regression is an approach taken to control for covariates whereby one can examine the change in the parameter of interest with sequential addition of the predictors of interest to the model. A strength of sequential modelling of groups of risk factors according to the timing of occurrence (before pregnancy, during pregnancy but before labor and delivery, or during labor and delivery) is that it can reduce problems in interpreting the results of multivariable modelling of potential causal intermediates [1-5].

We have included the following text in the Methods to describe the rationale behind using this sequential regression modelling approach (lines 238-242): “Secondly, since changes in maternal characteristics (e.g., increases in advanced maternal age) and maternal comorbidity (e.g., diabetes) can lead to changes in obstetric practice factors (e.g., increases in epidural use), we used sequential log-binomial models to identify the effect of each factor and each group of factors on the relationship between year or period and CD.”

References

1. Kramer MS, Platt R, Yang H, et al. Secular Trends in Preterm Birth: A Hospital-Based Cohort Study. JAMA 1998;280:1849–54.
2. Joseph KS, Young DC, Dodds L, O'Connell CM, Allen VM, Chandra S, et al. Changes in maternal characteristics and obstetric practice and recent increases in primary cesarean delivery. Obstet Gynecol 2003;102:791-800.
3. Räisänen S, Gissler M, Sankilampi U, Saari J, Kramer MR, Heinonen S. Contribution of socioeconomic status to the risk of small for gestational age infants–a population-based study of 1,390,165 singleton live births in Finland. Int J Equity Health 2013;12:1-8.
4. Kramer MS, Berg C, Abenhaim H, Dahhou M, Rouleau J, Mehrabadi A, et al. Incidence, risk factors, and temporal trends in severe postpartum hemorrhage. Am J Obstet Gynecol 2013;209:449-e1.
5. Ladfors LV, Muraca GM, Zetterqvist J, Butwick AJ, Stephansson O. Postpartum haemorrhage trends in Sweden using the Robson ten group classification system: a population‐based cohort study. BJOG 2022;129:562-71.

**Comment 4**: L140. Is this extension of the Robson classification routinely used?

***Response***: Yes, this is a commonly used modification of the Robson classification. In fact, this modification is detailed in the classification implementation manual authored by the World Health Organisation (WHO) [1]. Listed below are a few of many examples of the use of this modified classification in the extant literature [2-7]. We have included a citation to the WHO Implementation Manual to reference this modified classification. The manuscript now reads: “Data were grouped into Robson categories and modified from the 10-group system to a 12-group system, used commonly [9] to differentiate women in Groups 2 and 4 between those who had an intrapartum CD after induction (defined as 2a and 4a) and those who had a CD before labour onset (2b and 4b; Table 1).” (lines 176-179)

References

1. World Health Organization. Robson Classification: Implementation Manual. Geneva; 2017. Licence: CCBY-NC-SA3.0IGO.
2. Farine D, Shepherd D, Robson M, Gagnon R, Hudon L, Basso M, et al. Classification of caesarean sections in Canada: the modified Robson criteria. J Obstet Gynaecol Can 2012;34:976-9.
3. Betran AP, Vindevoghel N, Souza JP, Guelmezoglu AM, Torloni MR. A systematic review of the Robson classification for caesarean section: what works, doesn't work and how to improve it. PLoS ONE 2014;9:e97769.
4. Vogel JP, Betrán AP, Vindevoghel N, Souza JP, Torloni MR, Zhang J, et al. Use of the Robson classification to assess caesarean section trends in 21 countries: a secondary analysis of two WHO multi-country surveys. Lancet Global Health 2015;3:e260-70.
5. Gu J, Karmakar-Hore S, Hogan ME, Azzam HM, Barrett JF, Brown A, et al. Examining cesarean section rates in Canada using the modified Robson classification. J Obstet Gynaecol Can 2020;42:757-65.
6. Mayne L, Liu C, Tanaka K, Amoako A. Caesarean section rates: applying the modified ten-group Robson classification in an Australian tertiary hospital. J Obstet Gynecol. 2022;42(1):61-6.
7. Ladfors LV, Muraca GM, Zetterqvist J, Butwick AJ, Stephansson O. Postpartum haemorrhage trends in Sweden using the Robson ten group classification system: a population‐based cohort study. BJOG 2022;129:562-71.

**Comment 5**: L155. Are these comorbidity categories examples of the ones used, or the complete set?

***Response***: The complete set. We have clarified this by revising the sentence to now read: “We also adjusted for maternal comorbidity, which included chronic hypertension, pre-pregnancy diabetes, in-vitro fertilization and pre-eclampsia/eclampsia.” (lines 197-199)

**Comment 6**: L166. I have used GEE procedures with the modified Poisson regression to get estimates of rate ratios/relative risk (i.e. the robust standard error), in these cases each row/observation of data was independent so the independent working structure matched the data. If there are multiple deliveries per mother in the data, is an independent working structure realistic, i.e. compared to an exchangeable/CS structure?

***Response***: Thank you for picking up this typographical error, we indeed used an exchangeable correlation structure in our GEE methods. We have corrected this in the revised manuscript, which now reads: “These analyses were performed on individual pregnancies, which made it possible for a woman to contribute more than one pregnancy in the Robson Groups that included multiparous women. Therefore, we used generalized estimating equations (with the mother’s identification as a cluster and assuming an exchangeable correlation structure), with adjustment for the possible correlation in outcome that could be introduced by subsequent births by the same mother.”(line 211-216)

**Comment 7**:

a) L171. This does assume that there is no correlation of missing BMI status with other covariates. This is assessed later on (seems to be fine), I would describe this approach as 'missing as indicator' in the methods.

***Response***: The use of a missing indicator for BMI is now described as suggested on line 219: “Missing BMI values were modelled using the missing-indicator method”, and on lines 404-406:

“Further, 13.8% of women (7.9% in Sweden and 28.3% in BC) in our study had missing values for pre-pregnancy BMI and we included these women in our main models using a missing indicator approach.”

b) I would also specifically say you completed a complete-case analysis with respect to the other variables.

***Response***: We now explicitly state that we conducted a complete-case analysis with respect to the other variable on lines 219-222. “Records with missing values for other variables were excluded as they did not exceed 3% of the total study population; thus, we conducted a complete-case analysis with respect to these variables (Figure 1).”

c) I wasn't clear how many from each country were excluded on this basis - it would be helpful to have this detail in a flow diagram describing how many deliveries were initially included and then what remained after any exclusions for missing data.

***Response***: Table 1 in the revised manuscript now makes it clear how many deliveries were excluded due to missing information in each country. S5-S16 Tables show this distribution for each variable among each Robson Group.

As suggested, a flow diagram has been included (Figure 1 in revised manuscript) to illustrate the number of deliveries excluded due to missing data and the number included in the complete-case analysis.

d) Was there variation in rates of missingness (in BMI and the other variables) over time?

***Response***: The rate of missingness in BMI did decrease over the study period in both countries. In Sweden, the proportion of deliveries with a missing BMI value was 13.2% in 2004, and this decreased to 6.2 by 2016. In BC, these equivalent values were 30.4% in 2004 and 22.28% in 2016. Nonetheless, analyses restricted to individuals with no missing information on BMI and models with imputed BMI values yielded almost identical results for comparisons of CD rates between countries and within countries over time. All other variables did not materially vary in missingness over the study period.

**Comment 8:** L208. How did you decide on the selected number of imputations?

***Response*:** We aimed to use 15 imputation cycles to satisfy the simplified rule of thumb that the number of imputations should be similar to the percentage of cases that are incomplete [1,2]. While replicating sets of imputations and monitoring whether the estimates were stable between sets [3], we found that 10 imputations were sufficient for the estimates to stabilize.

References

1. Bodner TE. What improves with increased missing data imputations? Structural Equation Modeling: A Multidisciplinary Journal 2008;15:651-75.
2. White IR, Royston P, Wood AM. “Multiple imputation using chained equations: Issues and guidance for practice. Stat Med 2011;30:377-99.
3. Stuart EA, Azur M, Frangakis C, Leaf P. Multiple imputation with large data sets: a case study of the Children's Mental Health Initiative. Am J Epidemiol 2009;169:1133-9.

**Comment 9**: L286. Just checking the estimate and 95% CI here: 1.01 (0.99-1.04). This looks a bit odd - maybe just from rounding?

***Response***: Yes, this is correct, thank you for checking.

**Comment 10**: Appendix - Table S23. Why did some of the adjusted models exclude particular variables? Because of small cell sizes?

***Response***: Some of the adjusted models exclude particular variables because they are not relevant to all Robson groups. For example, we excluded the variable for parity among groups that included only nulliparous women. Another example is excluding the variable for occiput anterior/posterior fetal presentation during delivery among Robson groups with breech fetal presentation.

To clarify these exclusions, we have elaborated on these exclusions in the legend of Table 3 (previously Table 2). It now reads:

“^b^Adjusted model excluded parity due to group restriction to nulliparas.

^c^Adjusted model excluded position of the fetal head at delivery due to group restriction to non-cephalic fetal presentation or multiple gestation.

^d^Adjusted model excluded post-term delivery due to group restriction to preterm deliveries.” (page 33)

**Reviewer #2:** Thanks for opportunity to review this paper.

This is a novel and well-conducted analysis of retrospective birth data in 2 countries. The analysis addresses a well-known thorny issue in Robson-based analyses of CS usage. The methods are well-described.

**Comment 1**: Did the authors explore the possibility of differences in the validity/reliability of the 2 datasets, particularly for Robson variables, which might have contributed to inter-country differences?

***Response***: The nationwide Swedish Medical Birth Register includes prospectively recorded information from prenatal, delivery, and neonatal care on more than 98% of deliveries in Sweden and the validity of most variables has been assessed as being high [1]. A validation study [2] in 2014 compared pregnancy and delivery data in medical records with corresponding data in the Medical Birth Register. Data was re-abstracted from the medical records of women who delivered in nine Swedish hospitals across the country. The results showed high rates of agreement between the Medical Birth Register and medical records for most variables. For example, with respect to variables used in the Robson classification, 97% agreement for parity, 91% for plurality, 95% for mode of delivery, and 99% for gestational age. More than half of the variables studied demonstrated identical information in both data sources to a level of 95% or more.

The British Columbia Perinatal Database Registry (BCPDR) contains comprehensive, population-based perinatal data from 99% of births in the province. The quality of the data in this database are continually assessed by means of audits and consistency checks, and information in the database has been validated [3-5]. It is used routinely for health care planning, surveillance and research. A provincial chart re-abstraction study published in 2015 assessed the validity of information in the BCPDR, in which 1,084 maternal and 1,142 newborn charts were abstracted [3]. Many important clinical and population health variables in the BCPDR had excellent validity. Specifically focusing on variables used in the Robson classification, the intraclass correlation coefficient (ICC) for gestational age and previous CD were both 0.99. The sensitivity and specificity for plurality were both 100%. Onset of labour variables had sensitivity and specificity estimates of 97.9% (95% CI 96.4%-98.8%) and 94.4% (95% CI 91.2%-96.7%), respectively for spontaneous onset, and 93.9% (95% CI 90.2%-96.5%) and 98.7% (95% CI 97.7%-99.3%), respectively for induced labour. Lastly, the variable for fetal presentation at delivery had a sensitivity and specificity of 91.2% (95% CI 82.8%-96.4%) and 99.5% (95% CI 98.8%-99.8%), respectively.

References

1. Källen B, Källen K. The Swedish Medical Birth Register—a summary of content and quality. The Swedish Centre for Epidemiology. The National Board of Health and Welfare, Stockholm, Sweden; 2003.
2. Petersson K, Persson M, Lindkvist M, Hammarström M, Nilses C, Haglund I, et al. Internal validity of the Swedish Maternal Health Care Register. BMC Health Serv Res 2014;14:364.
3. Frosst G, Hutcheon J, Joseph KS, Kinniburgh B, Johnson C, Lee L. Validating the British Columbia Perinatal Data Registry: a chart re-abstraction study. BMC Pregnancy Childbirth 2015;15:123.
4. Frosst G. Validity of pre-pregnancy body mass index (BMI) information derived from a population-based perinatal database. Healthy Mothers and Healthy Babies: New Research and Best Practice Conference 2014. Vancouver, British Columbia.
5. Joseph KS, Fahey J. Validation of perinatal data in the Discharge Abstract Database of the Canadian Institute for Health Information. Chronic Dis Can 2009; 29: 96–100.

We also assessed the Standard Robson Classification Report Table (Table 2 in the revised manuscript) using standardized criteria [1] to assess the quality of data, type of population, and CD rates. This assessment is included in the Table below. There are two sets of benchmarks included in the assessment, the first was established by Dr. Michael Robson based on his international experience applying the classification system, and the second was established by the WHO Multi-country Survey on Maternal and Newborn Health (WHO MCS) [2,3]. Using data from this survey, a reference population was created that was restricted to the facilities with low CD rates and low intrapartum perinatal mortality as they were assumed to have few unnecessary CDs and good maternal/perinatal care [2,3]. The “reference population” included 42,637 women from 22 countries.

We now include this assessment in our methods section on lines 183-185: “We also assessed the data quality, population distributions, and Robson Group-specific CD rates against WHO-recommended benchmarks using standardized criteria [1]” and report the results on lines 275-276 of the Results: “The standard assessment of the data quality, obstetric population distribution, and Robson Group-specific CD rates is included in S1 Table.”

The table below is now included in the Supporting Information of the revised manuscript as S1 Table.

**S1 Table. Steps to assess quality of data, type of population, and cesarean delivery rates [1]**

| **Step** | **Interpretation by Robson [1]** | **Swedish population** | **BC population** | **WHO MCS reference population [2,3]** | **Interpretation** |  |
| --- | --- | --- | --- | --- | --- | --- |
| Data quality | | | | | | |
| Look at the size of Group 9 (singletons in transverse/ oblique lie) | It should be less than 1%. | 0.1% | 0.3% | 0.4% | As expected. |  |
| Look at the CD rate of Group 9 | It should be 100%. | 99.3 | 94.7 | 88.6% | Minor misclassification. |  |
| Type of population | | | | | | |
| Look at the size of Groups 1 + 2 (nulliparous women ≥37 weeks gestation singleton cephalic) | This usually represents 35-42% of the obstetric population. | 39.2% | 39.2% | 38.1% | As expected. |  |
| Look at the size of Groups 3 + 4 (multiparous women ≥37 weeks gestation singleton cephalic, without previous CD) | This usually represents about 30% of women. | 43.0% | 34.3% | 46.5% | As expected. Higher in Sweden due to a high proportion of multiparas without a previous CD. |  |
| Look at the size of Group 5 (multiparous women ≥37 weeks gestation singleton cephalic with previous CD) | It is related to the overall CD rate. The size of Group 5 is usually about half of the total CD rate. In settings with low overall CD rates, it is usually under 10%. | 8.6% | 12.8% | 7.2% | As expected. Size of Group 5 in BC similar to those reported in national US and Canadian analyses [4,5]. |  |
| Look at the size of Groups 6 + 7 (breeches in nulliparous & multiparous women) | It should be 3-4%. | 3.2% | 3.9% | 2.7% | As expected. |  |
| Look at the size of Groups 8 (multiple gestations) | It should be 1.5 -2%. | 1.4% | 1.6% | 0.9% | As expected. |  |
| Look at the size of Group 10 (preterm cephalic singletons) | It should be less than 5% in most normal risk settings. | 4.2% | 6.8% | 4.2% | Higher rates in BC suggest higher rates of provider-initiated pre-labour CD. Similar to Group size reported in US and Canadian national analyses [4,5]. |  |
| Look at the ratio of the size of Group 1 vs Group 2 (nullipara term cephalic singletons spontaneous labour/nullipara term cephalic singletons induced or pre-labour CD) | It is usually 2:1 or higher | 3.8:1 | 2.2:1 | 3.3:1 | As expected. |  |
| Look at the ratio of the size of Group 3 vs Group 4 (multipara without previous CD, term cephalic singletons spontaneous labour/multipara without previous CD, term cephalic singletons induced or pre-labour CD) | It is always higher than the ratio of Group 1/Group 2 in the same population, i.e., larger than 2:1. | 5.1:1 | 3.7:1 | 6.3:1 | As expected. This is very reliable finding in confirming data quality and culture of the organization [9]. |  |
| Look at the ratio of the size of Group 6 vs Group 7 (nullipara breech /multipara breech) | It is usually a 2:1 because breeches are more frequent in nulliparous women than in multiparous women. | 1.7:1.0 | 1.4:1.0 | 0.8:1 | As expected. |  |
| Assessing CD rates | | | | | | |
| Look at the CD rate for Group 1 | Rates under 10% are achievable | 8.1% | 20.4% | 9.8% | High rates of CD in BC are consistent with previous reports using different data sources [2]. Decomposing this high rate is one of the foci of the present analysis. |  |
| Look at the CD rate for Group 2 | Approximately 20-35% | 37.3% | 45.9% | 39.9% | Since Group 2b is relatively small, the high rates of CD in Group 2 in both Sweden and BC are a result of the high CD rate in Group 2a. This may indicate poor success rates for induction or poor choice of women to induce and consequently a high rate of CD in Group 2a. High rates of CD in BC are consistent with previous reports using different data sources [2]. |  |
| Look at the CD rate for Group 3 | Normally, no higher than 3.0%. | 1.6% | 2.6% | 3.0% | As expected. |  |
| Look at the CD rate for Group 4 | It rarely should be higher than 15% | 21.5% | 13.1% | 23.7% | CD rate is high in Sweden since the size of Group 4b (pre-labour CD) is large. May indicate a data issue whereby women with a previous CD are included (when they should be in Group 5). May also be due to high rates of CD on maternal request due to previously traumatic or prolonged labour. |  |
| Look at the CD rate for Group 5 | Rates of 50-60% are considered appropriate provided you have good maternal and perinatal outcome. | 51.6% | 81.3% | 74.4% | As expected. Higher rates of CD in BC consistent with rates in North America where a policy of scheduling pre-labour CD for women with a previous scar without attempting a trial of labour is common [4,5]. |  |
| Look at the CD rate for Group 8 | It is usually around 60%. | 54.7% | 69.6% | 57.7% | As expected. Higher rates in BC expected due to higher number of women with a previous CD. Similar to national estimates in the US and Canada [4,5]. |  |
| Look at the CD rate for Group 10 | In most populations it is usually around 30% | 29.4% | 30.8% | 25.1% | As expected. |  |
| Look at the relative contribution of Groups 1, 2 and 5 to the overall CD rate | These three groups combined normally contribute to 2/3 (66%) of all CD performed in most hospitals. | 57.8% | 69.0% | 63.7% | As expected. Higher relative contribution in BC due to higher rates of CD in both Groups 1 and 2. These higher rates in previous years are responsible for the larger size of Group 5 in BC. This larger size plus the and higher rates of CD in BC in Group 5 drive the higher contribution of these three groups compared with Sweden (and the MCS population). |  |
| Look at the relative contribution of Group 5 to the overall CD rate |  | 25.7% | 33.4% | 28.9% | Appropriate. Higher in BC since CD rates in Groups 1 and 2 have been high in previous years. |  |

WHO MCS, World Health Organization Multi-Country Survey; BC, British Columbia; CD, cesarean delivery.

MCS reference population was the population of the MCS with relatively low CD rates and, at the same time, with good outcomes of labour and childbirth.

References

1. World Health Organization. Robson Classification: Implementation Manual. Geneva; 2017. License: CCBY-NC-SA3.0IGO.
2. Souza JP, Betrán AP, Dumont A, de Muncio B, Gibbs Pickens C, Deneux-Tharaux C, et al. A global reference for caesarean section rates (C-Model): a multi-country cross-sectional study. BJOG 2016;123:427-36
3. Souza JP, Gülmezoglu AM, Vogel J, Carroli G, Lumbiganon P, Qureshi Z, et al. Moving beyond essential interventions for reduction of maternal mortality (the WHO Multi-country Survey on Maternal and Newborn Health): a cross-sectional study. Lancet. 2013;18:1747-55.
4. Hehir MP, Ananth CV, Siddiq Z, Flood K, Friedman AM, D’Alton ME. Cesarean delivery in the United States 2005 through 2014: a population-based analysis using the Robson 10-Group Classification System. Am J Obstet Gynecol 2018;219:105.e1–11.
5. Gu J, Karmakar-Hore S, Hogan ME, Azzam HM, Barrett JF, Brown A, et al. Examining cesarean section rates in Canada using the modified Robson classification. J Obstet Gynaecol Can 2020;42:757-65.

**Comment 2**: Line 158 - The consideration of 2 obstetric practice-related factors (Epidural anesthesia and post-term delivery) seemed somewhat brief to me and a potential major limitation. A number of practice or care-setting-related variables have been shown to be associated with CS rates, including but not limited to level of care (secondary vs tertiary), private vs public, antenatal care provision/provider, presence/absence of labour companion, continuity of care provider, VBAC policies, mandatory second opinion and others. I note that presence/absence of midwife-led care is dealt with through sensitivity analysis. I appreciate several of these are probably not be available in the individual level data, but it is necessary for the authors to explain the rationale for selection of only these 2 variables, and why others were not included or considered.

***Response***: The Reviewer is correct that there are several variables that we were unable to measure due to data constraints. We chose the two obstetric practice factors, epidural anesthesia and post-term delivery because: a) we hypothesized they would have substantial variability between the two countries and over time; and b) this information was reliably captured in both the Swedish Medical Birth Register and the British Columbia Perinatal Database Registry. This is now stated explicitly in the Methods (lines 201-203): “We chose these two obstetric practice factors because a) we hypothesized they would have substantial variability between the two countries and over time; and b) this information was reliably captured in both the MBR and the BCPDR.”

**Comment 3:**

a) 203 - The issue of BMI driving CS rates is one of significant debate. Some clarification on the timing of early pregnancy BMI measurement would be useful.

***Response:*** Clarification on the timing of early-pregnancy BMI measurement is now included in the methods (lines 195-197): “In Sweden and BC, maternal BMI in early pregnancy was calculated from self-reported height and weight or provider assessment at the first antenatal visit, which typically occurs within the first 14 weeks of gestation [22,23]”

b) Also, 28% seems quite high for a missing rate and I did wonder if these are missing at random or not, and whether multiple imputation is appropriate in this context.

***Response***: We assumed that the data are missing-at-random (MAR), for the variables included in the imputation model. BMI is likely missing at random, as it appears that some obstetric practices document height and weight and others do not. While this assumption is empirically unverifiable, this is a reasonable assumption [1,2]. Since MAR assumes that the observed missingness in a given dataset is dependent solely on the observed data, the MAR assumption becomes more plausible when more characteristics (and their relationships with other variables) are included in the imputation model [3].

Multiple imputation allowed for maximum use of the available data, and we included a number of demographic and clinical variables in the imputation model (i.e., maternal age, parity, smoking, pre-existing diabetes, preeclampsia, chronic hypertension, in-vitro fertilization, gestational age, infant birth weight, congenital anomaly) to make the MAR assumption more plausible. As our findings from the imputation models were consistent with the complete case analysis findings, implying that bias due to missing data is unlikely. We have included the following information in the Discussion to further contextualize the limitations due to BMI missingness: “Since we do not know if the BMI distribution was different among those with missing vs. available BMI information, it is challenging to speculate on the direction and magnitude of bias introduced by missing data for BMI [34]. Confirmation of our findings in a cohort with complete BMI data would be valuable.” (lines 408-411)

References

1. Harel O, Zhou XH. Multiple imputation: review of theory, implementation and software. Stat Med 2007;26:3057-77.
2. Greenland S, Finkle WD. A critical look at methods for handling missing covariates in epidemiologic regression analyses. Am J Epidemiol 1995;142:1255-64.
3. Collins LM, Schafer JL, Kam CK. A comparison of inclusive and restrictive strategies in modern missing-data procedures. Psychol Methods 2001;6:330-51.

**Reviewer #3:** This paper reports the findings of an innovative approach to contribute to the understanding of the CD epidemiology that is traditionally done using the well recognized and globally validated Robson classification. The authors add maternal, fetal and obstetrical practice factors to the Robson classifications in Sweden and BC.

The paper is well written, well established methodology taken into account models of obstetrical care, and reports on a huge sample size over a 12 years period of time, analyzing population based data sets. The findings are important, significant and show the weight of mainly maternal factors adding value to the Robson classification for clinical practice.

**Comment 1**: One question however remains: the constant lower and stable CD rates in Sweden as compared to the higher and increasing rates in BC can not be fully explained by differences in maternal or obstet practice factors. Did the authors have the opportunity to compare the CD rates in BC between midwifery and ob/gyn led models? or financial/ cost factors in the health care models of both sites?

***Response***: We performed a sensitivity analysis aimed at addressing differences in maternity care models in each country. In this analysis we also compared deliveries in Sweden with those in BC restricted to women with midwifery care. The results are included in Tables S21 and S22. In summary, the main differences in the rates of CD in this restricted group compared with all deliveries in BC was a lower rate of CD in women with a previous CD (Group 5), women with multiple pregnancies (Group 8), and those who delivered preterm (Group 10; Table S20). Among these women as well, adjustment for maternal, fetal and obstetric practice factors attenuated the difference in CD rates in BC vs Sweden in Group 1, 2, 4, and 10 but not in Groups 5 and 8.

Unfortunately, an analysis of costs associated with CD was beyond the scope of this analysis as we did not have financial information. Any future studies of the economic impacts of CDs in each sub-group of the obstetric population will need to incorporate information on maternal and perinatal outcomes, ideally in the short- and long-term.

**Reviewer #4:** This is a very interesting paper of almost 2 million deliveries between 2004 and 2016 in Sweden and BC Canada. It significantly adds to the literature. As the authors discuss, there has been a significant amount of work published on the Robson Ten Group Classification system, but the effect of controlling for maternal, obstetric and fetal factors the way the authors do in this work is novel.

**Comment 1**: I have one comment in relation to lines 218 - 220

"The rate of CD was higher in BC compared with Sweden in all Robson Groups except in Group 4 (parous women with a single, cephalic fetus at term gestation with an induction or CD prior to labour) and among deliveries with a transverse/oblique-presenting fetus (Group 9)" Group 9 is often used as a quality control mechanism for the data and the CS rate in this group should be 100%. Therefore, it may be reflective of the quality of the data collection rather than an actual difference itself.

***Response:*** We assessed the Standard Robson Classification Report Table (Table 2 in the revised manuscript) using standardized criteria [1] to assess the quality of data, type of population, and CD rates. The first and second steps in this assessment pertain to Group 9 since, as mentioned above, it is often used as a data quality measure. Firstly, the size of Group 9 is expected to be less than 1% of the obstetric population. In our study this criterion is satisfied as Group 9 represents 0.1% of deliveries in Sweden and 0.3% in British Columbia. Secondly, the CD rate should be 100%. In our study, the CD rate in Group 9 was 99.3% in Sweden and 94.7% in British Columbia. This proportion of misclassification is somewhat expected as it is far less than that reported is previous national analyses in the United States and Canada [2,3]. The complete assessment is included in the revised manuscript as S1 Table.

Regarding the high rate of CD in Group 4 in Sweden, we included the following statement in the discussion: “The relatively large size of Group 4b (multiparous women with pre-labour CD) in Sweden may indicate misclassification of women with a previous CD (i.e., they should have been assigned to Group 5); alternatively, this may indicate higher rates of CD on maternal request due to a variety of reasons, such as previously traumatic or prolonged labour.” (lines 412-415)

References

1. World Health Organization. Robson Classification: Implementation Manual. Geneva; 2017. Licence: CCBY-NC-SA3.0IGO.
2. Hehir MP, Ananth CV, Siddiq Z, Flood K, Friedman AM, D’Alton ME. Cesarean delivery in the United States 2005 through 2014: a population-based analysis using the Robson 10-Group Classification System. Am J Obstet Gynecol 2018;219:105.e1–11.
3. Gu J, Karmakar-Hore S, Hogan ME, Azzam HM, Barrett JF, Brown A, et al. Examining cesarean section rates in Canada using the modified Robson classification. J Obstet Gynaecol Can 2020;42:757-65.

**Comment 2**: Notwithstanding this minor point, I believe that this work is very worthy of publication and gives a very clear insight into the significant spatio-temporal differences in certain Robson groups.

***Response***: Thank you.

**Reviewer #5:** Thank you for the opportunity to review your paper and i appreciate all the hard work that has gone into it. I would like to make the following points.

**Comment 1**: The TGCS has never been intended to be anything more than a starting point and a structure within which all other epidemiological variables, differences in practice and events and outcomes are analysed. I am not sure that the readers of the paper will understand that when you discuss it in the paper.

***Response***: To ensure that readers are clear on the original aim of the Robson Classification system, we have included the following text in the Discussion: “Our findings reinforce that, although often misinterpreted, the Robson classification was not meant to be an endpoint of CD comparison, but rather a starting point or initial framework within which determinants and outcomes of CD can be analysed [10].” (lines 427-430)

Reference

10. Robson M. The Ten Group Classification System (TGCS)–a common starting point for more detailed analysis. BJOG 2015; 122: 701.

**Comment 2**: I would always start by using the standard TGCS table first. There are a number of reasons why this is strongly advised

***Response***: Table 2 is now formatted to adhere to the standard Robson Classification Report Table format.

**Comment 3**: I get the impression from reading it that you have used data that is available rather than comment on the unavailability/quality of important information that should be there and if not that is the surely the big problem? So the TGCS structure is accurately collected (less then 1% of women could not be classified) but no real information on perinatal (in particular neonatal) outcomes or labour and delivery practice. Even BMI was not available in 28 % of women in BC.

There was no information on oxytocin rates, indications for CS or indications for pre-labour CS

***Response***: We have commented on our choice of obstetric practice factors on lines 201-203: “We chose these two obstetric practice factors because a) we hypothesized they would have substantial variability between the two countries and over time; and b) this information was reliably captured in both the MBR and the BCPDR.” We further discuss the unavailability of relevant determinants of CD, including oxytocin rates, on lines 415-417: “Finally, we were not able to adjust for important intrapartum details such as duration of labour, oxytocin augmentation, and fetal surveillance, because our data sources lacked such information.” and the quality the information included in our data sources is discussed on lines 403-404 and S1 Table. We acknowledge the limitations of our analysis due to missing values for BMI on lines 404-406 and carry out analysis using the missing-indicator approach, complete-case analysis, and multiple imputation with no material difference in results. Please see the response to Reviewer 2, Comment 1 for a detailed response regarding accuracy of these data.

The relationships between CD rates and maternal and perinatal outcomes was beyond the scope of this analysis.

**Comment 4**: I feel that there is a simple message from your TGCS data and that is that there are significant differences in CS rates between Sweden and BC. There are also more conclusions/hypotheses that you could have made from the raw TGCS table but you did not and i would be happy to help with that. With a good classification system there are only 3 reasons why there is a difference in either the sizes of the groups or the incidence of events within the groups. They are data quality, significant epidemiological variables and lastly differences in practice. By far the most common reason in perinatal data is data quality (including definitions)

***Response***: We assessed the Standard Robson Classification Report Table (Table 2 in the revised manuscript) using standardized criteria [1] to assess the quality of data, type of population, and CD rates. This assessment is included in the Table above (response to Reviewer 2, Comment 1) and in the revised manuscript as S1 Table. There are two sets of benchmarks included in the assessment, the first was established by Dr. Michael Robson based on his international experience applying the classification system, and the second was established by the WHO Multi-country Survey on Maternal and Newborn Health (WHO MCS) [2,3].

References

1. World Health Organization. Robson Classification: Implementation Manual. Geneva; 2017. Licence: CCBY-NC-SA3.0IGO.

2. Souza JP, Betrán AP, Dumont A, de Muncio B, Gibbs Pickens C, Deneux-Tharaux C, et al. A global reference for caesarean section rates (C-Model): a multi-country cross-sectional study. BJOG 2016;123:427-36

3. Souza JP, Gülmezoglu AM, Vogel J, Carroli G, Lumbiganon P, Qureshi Z, et al. Moving beyond essential interventions for reduction of maternal mortality (the WHO Multi-country Survey on Maternal and Newborn Health): a cross-sectional study. Lancet. 2013;18:1747-55.

**Comment 5**: While i absolutely agree with you that that the TGCS is only a structure and to make further conclusions in validating the difference between CS rates you need more information I am not sure with the limited data that you have presented that we are any further forward. The real point that your paper shows is how poor we all are in collecting routine perinatal data either because of a lack of resources or/and discipline together with a complete lack and agreement of further classifications. For example indications for CSs and inductions. Also your comment 369-371 supports this referring to lack of information on oxytocin, length of labour and fetal surveillance.

***Response***: The study inferences are limited but they do advance our understanding of factors that should be considered when comparing spatio-temporal differences in CD rates in some Robson Groups. We have included the following sentence to address the need for collecting quality, routine perinatal data: “These results also highlight the importance of comprehensive and accurate perinatal data collection to ensure public health initiatives regarding CD rates are suitably prioritized and evidence-informed.” (lines 430-432)

**Comment 7**: As far as the temporal and spatial differences in CS rates it is clear in the standard description of TGCS methodology that before considering the differences in CS rates you must examine any changes in the sizes of the groups first over the same period of time. You made no comment on that and even changed the design of the TGCS table by placing the column indicating the sizes of the group after the column showing the CS rates in each of the groups. This is confusing to readers who use the TGCS when each new paper invents their own way of designing the TGCS table. These comments are meant to be constructive and i think you should rethink what the main conclusion of what all your hard work has really shown especially the deficiencies. I would be happy to help in any way i can.

***Response***: We have shown the changes in Robson group sizes over the study period in the Swedish and Canadian cohorts in Figure 1, Panel B (Groups 1-5) and have amended Table 2 to reflect the format of the standard Robson classification reporting table. The column indicating the size of each group now appears before the column indicating the CD rates in each group.

We also have added an assessment of the Standard Robson Classification Report Table (Table 2 in the revised manuscript) using standardized criteria [1] to assess the quality of data, type of population, and CD rates. This assessment compares Robson Group sizes, ratios, relative contributions to the CD rate and CD rates to two sets of benchmarks: 1) by Dr. Michael Robson based on his international experience applying the classification system and 2) by the WHO Multi-country Survey on Maternal and Newborn Health (WHO MCS) [2,3].

References

1. World Health Organization. Robson Classification: Implementation Manual. Geneva; 2017. Licence: CCBY-NC-SA3.0IGO.

2. Souza JP, Betrán AP, Dumont A, de Muncio B, Gibbs Pickens C, Deneux-Tharaux C, et al. A global reference for caesarean section rates (C-Model): a multi-country cross-sectional study. BJOG 2016;123:427-36

3. Souza JP, Gülmezoglu AM, Vogel J, Carroli G, Lumbiganon P, Qureshi Z, et al. Moving beyond essential interventions for reduction of maternal mortality (the WHO Multi-country Survey on Maternal and Newborn Health): a cross-sectional study. Lancet. 2013;18:1747-55.
