# Supplementary material for: Crude and adjusted comparisons of cesarean delivery rates using the Robson classification: A population-based cohort study in Canada and Sweden, 2004 to 2016
Source: PLoS Med. 2022 Aug 1;19(8):e1004077. doi: 10.1371/journal.pmed.1004077 (PMC9377587; doi:10.1371/journal.pmed.1004077)
Supplement: Attachment — Submitted filename: R2. Response to Reviewers 28jun22.docx [file pmed.1004077.s035.docx]

**Requests from the Editors**

**Comment 1.** Response to reviewers: Please completely address the remaining comments of Reviewer 1 and Reviewer 5. Please carefully consider and respond to the comments provided by Reviewer 5 below.

***Response***: We have responded below to each of the comments from Reviewer 1 and Reviewer 5.

**Comment 2.** Abstract: Methods and Findings: Line 45: Please revise the sentence for grammar.

***Response***: This sentence has been revised and now reads: “We conducted a population-based cohort study of deliveries in Sweden (January 1, 2004-December 31, 2016; n=1,392,779) and BC, Canada (March 1, 2004-April 31, 2017; n=559,205).” (lines 46-47)

**Comment 3.** Abstract: Methods and Findings: Line 45-46: Please separately mention numbers of deliveries for Sweden and BC.

***Response***: The revised abstract now separately mentions the number of deliveries in Sweden and British Columbia. “……population-based cohort study of deliveries in Sweden (January 1, 2004-December 31, 2016; n=1,392,779) and BC, Canada (March 1, 2004–April 31, 2017; n=559,205).” (lines 46-47)

**Comment 4.** Abstract: Line 54: Please use “women with overweight/obesity” in this sentence.

***Response***: Line 55 has been revised as requested.

**Comment 5.** Line 55-56: Please also provide the p for trend for changes over time in each country.

***Response***: The p for trend for the changes in cesarean delivery rates over time in each country are now included in lines 56-58. “The CD rate in Sweden was stable at approximately 17.0% from 2004 to 2016 (P for trend=0.10), while the CD rate increased in BC from 29.4% to 33.9% (P for trend<0.001).”

**Comment 6.** Abstract: Line 75-76: We suggest revising to “These findings suggest that incorporating these factors into explanatory models may be useful for evaluating cesarean delivery rates in populations.” or similar.

***Response***: We have revised lines 75-76 in the abstract to read: “These findings suggest that incorporating these factors into explanatory models using the Robson classification may be useful for ensuring that public health initiatives regarding CD rates are evidence-informed.” (lines 76-78)

**Comment 7.** Abstract: Conclusions: Please provide 1 sentence of additional interpretation of the findings (e.g. what is the significance of the fact that adjustment for these factors explains different proportions of variation in cesarean delivery between Robson groups? What is the significance that temporal changes in rates can be explained by adjustment for some groups/for Canada?).

***Response***: We included the following sentence in the Abstract conclusion to further interpret our findings: “These findings suggest that incorporating these factors into explanatory models using the Robson classification may be useful for ensuring that public health initiatives regarding CD rates are evidence-informed.” (lines 76-78)

**Comment 8.** Author summary: Line 98-99: Please clarify this, as the results do not indicate changes in CD rates over time in Sweden.

***Response***: Although there was no significant change in the CD rate in Sweden in the overall obstetric population, there were significant changes in the CD rate over time in some Robson Groups. For example, in Sweden in Group 2a, the rate of CD decreased from 28.6% to 24.3% (P for trend<0.001) and in Group 5 the CD rate increased from 49.4% to 52.8% (P for trend<0.001; Table S3).

We have clarified this point in lines 95-97: “Adjustment for maternal, fetal and obstetric practice factors explained a substantial fraction of the temporal change in CD rates among some Robson groups in Canada but had little impact on temporal changes in CD rates among Robson groups in Sweden.”

**Comment 9.** Author summary: Line 101-102: Please be sure that the author summary points do not reproduce what is written in the Abstract.

***Response***: We have removed any author summary points that reproduce what is written in the Abstract.

**Comment 10.** Methods: Please specify the significance level used (e.g., P<0.05, two-sided) and the statistical tests used to derive p values reported.

***Response***: Lines 258-261 now include this information: “The a priori level of statistical significance was set at a 2-sided p value<0.05 for all analyses. The Cochran-Armitage test was used to test for significance of linear trend and the Wald chi-square test was used to test significance of differences in CD rates in the crude and adjusted regression models.”

In addition, the statistical test used to derive p values and the significance level has been specified below each Table and Supplementary Information Table containing p-values.

**Comment 11**. Results: Please provide both 95% CIs and p values for all applicable results reported in the revised Results.

***Response***: We have included both 95% CIs and p values for all applicable results as requested.

**Comment 12.** Discussion: Line 374: Please clarify if this is 5.2% of the 8.6% difference in CD rate for group 2 between Canada and BC.

***Response***: We have clarified this sentence in lines 374 to 378. “The effect of controlling for maternal, fetal and obstetric factors on the spatial comparisons differed by Robson Group and ranged from no effect (e.g., among deliveries with breech presentation; Groups 6 and 7) to explaining up to 5.2% of the absolute 8.6% difference in CD rates in BC vs Sweden in Group 2 (nulliparous women with a term, cephalic fetus with induced labour or a CD without labour).”

**Comment 13.** Discussion: Line 380: “...but had little impact on temporal changes in CD rates in Sweden.” This is somewhat confusing as the results did not indicate that there were significant temporal changes in CD rates in Sweden. Please clarify.

***Response***: Although there was no significant change in the CD rate in Sweden in the overall obstetric population, there were significant changes in the CD rate over time in some Robson Groups (e.g., Robson Groups 2a and 5).

We have revised lines 381 to 384 to clarify this point: “Adjustment for maternal, fetal and obstetric practice factors explained a substantial fraction of the temporal change in CD rates in BC in Robson Groups 1 and 2a but had little impact on the temporal changes in CD rates observed among specific Robson Groups in Sweden (such as Groups 2a and 5).”

**Comment 14.** Discussion: Line 381: Please clarify if “industrialized settings” might be more accurately described as high income settings, or similar.

***Response***: We have changed “industrialized settings” to “high-income settings” on Line 385.

**Comment 15.** References: Please include punctuation after journal title abbreviations. Please remove the registered trademark symbol from reference 30. Please use the "Vancouver" style for reference formatting, and see our website for other reference guidelines <https://journals.plos.org/plosmedicine/s/submission-guidelines#loc-references>

***Response***: We have corrected the references as requested.

**Comment 16.** Tables and Supporting Information tables: Please quantify the presentation of results with both 95% CIs and p values where applicable. When reporting p values, please use 2 decimal places for p = 0.01 or larger, and 3 decimal places if smaller. Please report as p<0.001 where applicable. Please check that all abbreviations are defined in legends (e.g. CD).

***Response***: We have included both 95% CIs and p values in the Tables and Supporting Information Tables using the recommended conventions. We have included all abbreviations in the legends.

**Comment 17**. Supporting Information Tables and Figures: Please provide a legend for each table/figure.

***Response***: A legend for each Supporting Information Table and Figure has been provided.

**Comment 18**. S1 Table: Please use the "Vancouver" style for reference formatting, and see our website for other reference guidelines <https://journals.plos.org/plosmedicine/s/submission-guidelines#loc-references>

***Response***: The references for Table S1 have been corrected.

**Comment 19.** S20 Table: Please clarify the title of the table, as it appears that all years (2008-2016) are being compared to 2004-2007. Please also include p values.

***Response***: The title of S20 Table has been amended to “S20 Table. Crude and adjusted rate ratios (RR) for cesarean delivery in 2008-2010, 2011-2013 and 2014-2016 vs 2004-2007 among women in Robson Groups 1, 2a, and 5 after sequential adjustment for maternal characteristics, obstetric practice factors and fetal/infant characteristics, Sweden and British Columbia, Canada”.

P-values have been included.

**Comments from the Reviewers**

**REVIEWER #1**: Thanks for the revised manuscript and responses to my original queries. Your explanation to my question about variation in CD rates was extremely enlightening and I can appreciate why this is not an easy question to answer.

The rest of the changes to the manuscript cover my initial questions (apart from a few minor points below) and I think you have given a fair assessment of the limitations with the missing BMI values. Apart the BMI the amount of missing data is a fairly small fraction and it would take a very unusual missingness mechanism to affect the main results of the study.

**Comment 1**: If using the 'sequential' term throughout the manuscript I would provide the explanation about what exactly you did (i.e. fitting a series of models with more covariates that could mediate the relationships of interest) to the methods so it's clear for readers. There are some statistical estimation methods that use this term and so best to make sure there's no confusion.

***Response***: We have included the following sentence for clarification in the Methods (lines 239-241). “The sequential approach was carried out by fitting a series of models with additional groups of factors added to each model in the sequence outlined above to quantify the contribution of each group of factors to CD trends over time.”

**Comment 2**: For Table S3 could you just add an brief explanatory note about where there was no p-value for some of the time-series (i.e. 2b, where it stayed the same across time)?

***Response***: We have included a note below Tables S3 and S4. “^‡^Groups 2b and 4b are restricted to women with a cesarean delivery."

**Comment 3**: For Tables S5-S16, I would suggest the term 'unbalanced' instead of 'significant' as the convention is for 'significant' to be used with null hypothesis statistical testing.

***Response***: We have revised the text beneath Tables S5-S16 to read: “*Standardized difference values > 0.1 are considered indicative of an imbalance between groups.”

**REVIEWER #2**

**Comment 1:** Happy with these responses. I am impressed at the thoroughness of the response to reviewer letter, many thanks.

***Response***: Thank you.

**REVIEWER #5**

**Comment 1:** I do not disagree with your conclusion that you need other data in addition to the TGCS to explain differences in the caesarean section. Everybody would agree with that even before your study

I do however disagree that the criteria you have selected prove that or indeed we should encourage clinicians to collect those criteria before more relevant intrapartum data is collected. (i find it extraordinary that you cannot get information on indications, oxytocin rates or fetal outcome)

***Response***: We agree that the maternal, obstetric practice, and fetal/infant factors included in our analyses are not exhaustive and may not include important factors that are not represented in the Robson classification. In fact, we highlight in the Abstract (line 66) , Results (line 350), and Discussion (lines 375, 381-384) that in some populations, consideration of these factors had no effect on the CD rate. To further clarify this point, we have included the following sentence in the Discussion: “The maternal, obstetric practice, and fetal/infant characteristics included in our analyses were not exhaustive, and it is possible that the inclusion of other factors not represented in the Robson classification scheme (e.g., duration of labour, indication for CD) could have even larger impacts on CD rates.” (lines 442-445)

We do not suggest that the characteristics we included in our models should take priority over other determinants of CD and recommend comprehensive perinatal data collection on lines 440-442:

“These results also highlight the importance of comprehensive and accurate perinatal data collection to ensure public health initiatives regarding CD rates are suitably evidence-informed and prioritized.”

Unfortunately, our data sources do not contain reliable information on indication for cesarean delivery or rates of labour augmentation with oxytocin. We have expanded our limitations to explicitly include these and other weaknesses: “Finally, we were not able to account for important intrapartum details such as indication for CD, duration of labour, details regarding oxytocin augmentation and fetal surveillance because our data sources lacked such information.” (lines 419-422)

The analysis of fetal outcome is beyond the scope of this study. This study is already extremely dense, and the addition of another objective would detract from the current study objectives. Our group is working on an analysis of these data with relation to perinatal outcomes in a separate project. We fully agree that this is a much-needed next step and acknowledge this on lines 434-437 “While our study explained spatio-temporal variations in CD rates, it did not address the perinatal and maternal morbidity and mortality that was caused or prevented by the observed differences in CD rates. Future studies are required to evaluate the differences in CD rates in each Robson group within the context of maternal, fetal and infant morbidity and mortality.”

**Comment 2:** As a clinician i would have liked to like to see in your paper the TGCS as you have presented and within each of the groups a detailed analysis of relevant events and outcomes including epidemiological data. I also would expect to see in a paper about differences in cs rates a comparison of the indications and also other fetal and maternal outcomes. That way i would be able to compare my own data with yours. There should also be some description of the clinical processes and how they differ in the two regions.

***Response***: To enable the comparison of our results with other clinical settings, we have included several details of the frequency and distribution of all the relevant maternal, clinical and fetal/infant factors available in our data sources (Table 1). We have provided these detailed data disaggregated by Robson Group and country (Tables S5-S16), as well as over time in (Figures S2-S4) to facilitate an assessment of generalizability. In our analysis of the quality of our data and the types of populations we studied (Table S1) we provided details about clinical features in each population and references to provide insight into the cesarean delivery rates observed in our study. Unfortunately, our two data sources do not contain reliable and comparable information on indications for cesarean delivery or details regarding labour augmentation with oxytocin.

As mentioned above, the inclusion of perinatal outcomes was beyond the scope of this study. We are analyzing these data with regard to perinatal outcomes in a separate research project, which will include a focus on the similarities and differences in clinical processes in the two populations.

**Comment 3:** I also advise caution using 2a as a separate group without 2b. in your dataset it may make no difference but in datasets where group 2b is relatively bigger you may draw inappropriate conclusions. It is not a Robson group and i would be happy to explain the reasons why that may be a problem.

***Response***: The size of Group 2b is small in both Sweden and BC (relative size 1.1% and 1.2%, respectively; Table 2) and remained at these levels throughout the study period (Figure 2). These findings are described in Table S1 where we indicate: “Since Group 2b is relatively small, the high rates of CD in Group 2 in both Sweden and British Columbia are a result of the high CD rate in Group 2a. This may indicate poor success rates for induction or poor selection of women to induce and consequently a high rate of CD in Group 2a. High rates of CD in British Columbia are consistent with previous reports using different data sources [5].” (pg 2 of Supplementary Information Tables, row 4)

We describe Group 2 (as a whole) in the Robson Classification Report Table (Table 2), as well as subgroups 2a and 2b, to allow readers to understand: a) the original 10-group distribution, and b) how differences in induction rates and pre-labour CD contribute to the rates of CD in nulliparous women without a previous CD and the overall CD rates in different populations. In addition, our assessment of crude and adjusted differences in the CD rate between British Columbia and Sweden was conducted in Group 2 as whole (Table 3).

The revised manuscript contains the following paragraph in the Discussion to address this important issue on lines 423-433.

“We quantified the crude and adjusted temporal trend in CD in a commonly-used subdivision of Robson Group 2 restricted to women who had an induction (Group 2a) to increase the homogeneity of this group before assessing the contribution of maternal, fetal, and obstetric practice characteristics on temporal trends in CD. However, it is important to note that the analysis of any subdivision alone may be misleading without a consideration of the other subdivisions that compose the complete group (Group 2b in this case). In our analyses, the exclusion of Group 2b is unlikely to have had a substantial effect on temporal changes in the CD rate in Group 2 since the relative size of Group 2b was small (1.1% and 1.2% in Sweden and BC, respectively) and remained small throughout the study period. The relative size and high rate of CD in Group 2a are the reasons for this group’s position as one of the three largest contributors to the overall CD rate in both Sweden and BC.”

**Comment 4:** I really do believe that your paper shows the limitations of routine perinatal information at the present time and that should be the correct message from your paper nothing else

***Response***: We agree that the limitations of routine perinatal information are an important finding of this work. However, the central findings of this study are that Robson-specific CD rates vary widely in Sweden and Canada, and that without consideration of determinants of CD not included in the Robson classification, CD rate comparisons between populations using the Robson classification may be misleading. Although the Reviewer suggests above that these conclusions are common knowledge, this classification system is frequently used without such an understanding. Additionally, our study furthers this understanding by quantifying the individual and collective impact of several maternal, obstetric practice and fetal/infant factors not included in the Robson classification on spatio-temporal differences in CD among populations using detailed, validated, population-based birth registries from two countries and over a 13-year period.
