# Supplementary material for: Crude and adjusted comparisons of cesarean delivery rates using the Robson classification: A population-based cohort study in Canada and Sweden, 2004 to 2016
Source: PLoS Med. 2022 Aug 1;19(8):e1004077. doi: 10.1371/journal.pmed.1004077 (PMC9377587; doi:10.1371/journal.pmed.1004077)
Supplement: Attachment — Submitted filename: R3. Response to Editors_13jul22.docx [file pmed.1004077.s036.docx]

**Response to requests from Editors:**

1. Title Page: Please remove the funding and conflict of interest disclosure statements from the main text, and please be sure all information is submitted completely and accurately in the appropriate sections of the manuscript submission system.

**Response**: The funding and conflict of interest disclosure statements have bee removed from the title page. The information has been submitted accurately in the appropriate fields in the manuscript submission system.

2. Abstract: Methods and Findings: Line 59-63: Please also provide p values in addition to the 95% CIs reported for the main results comparing BC to Sweden.

**Response**: P-values have been added to the main results in addition to the 95% CIs on lines 54, 56 and 61.

3. Methods: Line 153: We suggest “data extraction” rather than “data manipulation” or similar.

**Response**: This sentence has been changed as suggested (line 140).

4. Methods: Line 183-184: Please revise to “Temporal trends in the relative contribution of each Robson Group to the overall CD rate were also described.” if this was intended.

**Response**: This correction is reflected in the revised manuscript on line 171.

5. Results: Line 269: Please revise to “...while women of advanced maternal age (≥35 years) and women with overweight or obesity (≥25 kg/m2) constituted 23.5% and 32.4% of the study population…” or similar.

**Response**: This correction is reflected in the revised manuscript on line 256.

6. References: For reference 11, please change the journal to PLoS One. Please check that all references use the "Vancouver" style for reference formatting, and see our website for other reference guidelines: <https://journals.plos.org/plosmedicine/s/submission-guidelines#locreferences>

**Response**: Reference 11 has been corrected and the Vancouver style for reference formatting has been used.

7. Page 29: Please remove the author contribution, declaration of interests, and data sharing sections from the main text. Please be sure all information is entered completely and accurately into the relevant sections of the manuscript submission system.

**Response:** The author contribution, declaration of interests and data sharing sections have been removed from the main text and all sections are included in the appropriate fields in the manuscript submission system.

8. S20 Table and S24 Table: Please clarify “sequential adjustment” in the table legends along the lines of Reviewer 1’s point: ““The sequential approach was carried out by fitting a series of models with additional groups of factors added to each model in the sequence outlined above to quantify the contribution of each group of factors to CD trends over time.”

**Response:** We have included the above information in the legends of Table 4, S20 Table and S24 Table.

9. Figure 4: Please revise the y axis scales consistent across all panels, beginning with zero. If not feasible to do this, please show a break in the axis / include a note in the legend to call attention to the fact that the y axis scales differ for the presentations of Robson Groups 1, 2a, and 5.

**Response:** Applying the same y axis scales across all panels would obscure some of the findings in the individual Robson group. As a result, we have revised Figure 4 to begin with zero and show a break in the axis. We have also included a note in the legend to call attention to the scale differences between panels. “Note that the y axis scales differ for the presentations of Robson Groups 1, 2a, and 5.” (lines 571-572)

10. STROBE Checklist: For item 22, please indicate the section as Funding or similar.

**Response**: The relevant sections have been added to item 22 in the revised S1 Checklist.

11. S1 Figure: Please include a legend. Please be sure to define what the error bars represent, and the abbreviation RG.

**Response:** The legend for S1 Figure is included on lines 689-692 with error bars and abbreviations defined.

12. S2 Table: Please clarify if these are crude or adjusted OR. If applicable, please present both adjusted and unadjusted results. In the legend, please mention the factors adjusted for, if adjusted analyses were done.

**Response:** The adjusted results have been added to S2 Table and the legend has been revised as suggested.
